# Reversible dysregulation of renal circadian rhythm in lupus nephritis

**DOI:** 10.1186/s10020-021-00361-9

**Published:** 2021-09-06

**Authors:** Rakesh Mishra, Ramalingam Bethunaickan, Celine C. Berthier, Zhengzi Yi, Joshua J. Strohl, Patricio T. Huerta, Weijia Zhang, Anne Davidson

**Affiliations:** 1 Institute for Molecular Medicine, Feinstein Institutes for Medical Research, 350 Community Drive, Manhasset, NY 11030 USA; 2 Department of Internal Medicine, Nephrology, University of Michigan, Ann Arbor, MI 48109 USA; 3 Department of Medicine, Mount Sinai Medical Center, One Gustave L. Levy Place, P.O. Box 1243, New York, NY 10029 USA

**Keywords:** SLE, Nephritis, Kidney, Circadian

## Abstract

**Background:**

We have found disruption of expression of major transcriptional regulators of circadian rhythm in the kidneys of several mouse models of lupus nephritis. Here we define the consequence of this disturbance with respect to circadian gene expression and renal homeostatic function in a mouse model of lupus nephritis.

**Methods:**

Molecular profiling of kidneys from 47 young and 41 nephritic female NZB/W F1 mice was performed at 4 hourly intervals over a 24 h period. Disruption of major circadian transcriptional regulators was confirmed by qPCR. Molecular data was normalized and analyzed for rhythmicity using RAIN analysis. Serum aldosterone and glucose and urine sodium and potassium were measured at 4 hourly intervals in pre-nephritic and nephritic mice and blood pressure was measured every 4 h. Analyses were repeated after induction of complete remission of nephritis using combination cyclophosphamide and costimulatory blockade.

**Results:**

We show a profound alteration of renal circadian rhythms in mice with lupus nephritis affecting multiple renal pathways. Using Cosinor analysis we identified consequent alterations of renal homeostasis and metabolism as well as blood pressure dipper status. This circadian dysregulation was partially reversed by remission induction therapy.

**Conclusions:**

Our studies indicate the role of inflammation in causing the circadian disruption and suggest that screening for loss of normal blood pressure dipping should be incorporated into LN management. The data also suggest a potential role for circadian agonists in the treatment of lupus nephritis.

**Supplementary Information:**

The online version contains supplementary material available at 10.1186/s10020-021-00361-9.

## Introduction

Circadian rhythm is a universal phenomenon by which organisms anticipate and respond to environmental changes by regulating sleep and feeding patterns, blood pressure, metabolism, detoxification and response to pathogens. Regulation of circadian behavioral rhythms is orchestrated by a “central clock,” located in the hypothalamus (Honma 2018; Mohawk et al. 2012). Core clock genes and machinery are also present in peripheral tissues (Mohawk et al. 2012) and up to 10% of cellular transcripts in any given organ oscillate in a circadian manner (Lamia et al. 2008; Panda et al. 2002).

At a molecular level, circadian rhythms are controlled by a set of core clock genes that regulate their own expression through a series of feedback loops. Clock and Bmal1 are master transcription factors that form heterodimers to induce the expression of *Per* and *Cry* genes. Per and Cry proteins translocate to the nucleus to repress *Clock /Bmal1* transcription; thus, the master clock genes inhibit their own transcription in a periodic fashion. Bmal1 and Clock also regulate the nuclear receptor genes *Ror* that activates and *Rev-erbα* (*Nrd1*) that represses *Bmal1* transcription. These core genes control the expression of many other genes which drive cascades of rhythmic gene expression (Stow and Gumz 2011; Reddy and O'Neill 2010; Levi and Schibler 2007; Kyriacou and Hastings 2010; Feng and Lazar 2012; Bechtold et al. 2010).

Renal circadian regulation influences renal blood flow, glomerular filtration rate and electrolyte excretion (Stow and Gumz 2011; Saifur Rohman et al. 2005; Firsov and Bonny 2018; Douma and Gumz 2018; Johnston and Pollock 2018; Carriazo et al. 2020). Dysfunction of renal excretory rhythms can alter renal sodium reabsorption; this is associated with loss of the normal nocturnal dip in the blood pressure which is a risk factor for cardiovascular disease and a significant predictor of chronic kidney disease (CKD) (Agarwal and Light 2009; Bankir et al. 2008; Burnier et al. 2007). There is also a link between clock output genes and drug detoxification in both the liver and kidneys with implications for delivery of therapeutic drugs (Gachon et al. 2006; Bowles et al. 2018).

In the course of molecular profiling studies of kidney mRNA from three mouse models of lupus nephritis (LN) using samples collected in the mornings we found altered expression of transcriptional regulators of circadian rhythm in nephritic kidneys (Bethunaickan et al. 2013). Here we ask whether this abnormality disrupts expression of diurnally regulated renal cellular transcripts and whether there are functional consequences with respect to homeostatic renal excretory rhythms.

## Materials and methods

### Mice

Mice were housed in our vivarium and maintained under conditions of 12 h light/dark periods and followed for the onset of proteinuria as previously described (Ramanujam et al. 2006; Schiffer et al. 2008). Groups of young and nephritic mice were moved to the reverse light cycle room for serum and urine collections and were acclimated for 2 weeks (Munn et al. 2011) prior to any experiments. We harvested perfused whole kidneys from 12 week old and nephritic 30–45 week old NZB/W (2–3 weeks after the onset of 300 mg/dl proteinuria and blood urea nitrogen < 25 mg/dl) mice at 4 h intervals over 24 h. To control for the general effects of age on renal circadian rhythms, kidneys were obtained from four 12-week-old and 36-week-old C57BL/6 mice at Zeitgeber time (ZT) 0 and 12 (Additional file 1: Table S1). To further control for age in the correct strain, nephritic mice aged 36–50 weeks were treated with triple CTLA4Ig, anti-CD40L and cyclophosphamide therapy from 48 to 72 h after first onset of fixed proteinuria > 300 mg/dl as previously described (Schiffer et al. 2003). Proteinuria was measured weekly and mice that maintained remission (proteinuria ≤ 30 mg/dl) were harvested after 8 weeks either at ZT0 or ZT12. For timed urine collections, mice were housed in their own cages on urine hydrophobic sand (Braintree, MA) (Hoffman et al. 2018) with gel food and gel water for up to 12 h. Urine was collected without disturbing the mice and pooled over several days for each timed collection.

### Blood pressure

Blood pressure was measured longitudinally in groups of young and nephritic mice using a CODA monitor (Kent Scientific, Torrington, CT) according to manufacturers’ instructions. After a 3 day period of acclimation to the apparatus, the same operator gently placed the mice in restrainers and warmed them to 35 °C for 15 min prior to measuring blood pressure. 10 cycles were measured at each time after 5 cycles of acclimation. Measurements in the dark cycle were made in a room with a red light so that the mice were not exposed to ambient light. Blood pressure measurements were performed no more than twice per individual mouse in each 24 h period and were carried out over 3–5 days.

### Measurement of mouse motor activity

Each animal was individually housed in a square-base plain chamber (40 cm side, 40 cm high) containing bedding, similar to that of the home cage as well as food and water. The chamber was located in a dedicated, quiet space without confounding external stimuli. A 50-W orange-red light bulb illuminated the chamber from above. A miniature near infrared video camera (CV502-MB, Marshall Electronics) was mounted above the chamber and connected to a 4-channel high definition video capture card (DeckLink Duo 2, Blackmagic Design). After a period of acclimatization, video was recorded at 30 frames per second with a resolution of 1920 × 1080 pixels with video tracking software (Wirecast 7 Pro, Telestream) and saved to disk in.mp4 file format. Captured videos were then analyzed using a behavioral tracking software (Ethovision XT 11.5, Noldus), which tracked the activity level of the mouse on a frame by frame basis. Data was expressed as the percentage of time that the mice were active per 30 min interval.

### Microarray and RNA sequencing

Total RNA was isolated from perfused mouse kidneys using TRIzol reagent (Ambion, Life Technologies). Quality of the RNA was verified on a NanoRNA chip (Agilent, Santa Clara, CA). 42 samples were run on microarray, 46 samples were run on RNASeq and 15 samples, all at ZT times 0 and 12 were run on both platforms (See Additional file 1: Table S1).

Microarray experiments using the Illumina MouseRef-8 v2.0 Expression BeadChip were performed on total RNA following standard protocols provided by the manufacturer (Illumina Inc. San Diego, CA). Whole-Genome Gene Expression Direct Hybridization assay was applied to RNA samples starting with 100 ng of total RNA for hybridization to the chips. The chips were scanned by HiScanner (Illumina Inc) and the background-subtracted intensity data was extracted by GenomeStudio Informatics System. The genes with low intensity (lowest 30% quantile of intensity values of all genes in all samples) in > 2/3 of the samples were excluded and the intensities of the remaining genes were log2 transformed and normalized at an equal global median value. Data has been submitted to GEO with the following accession number: GSE131894.

Paired-end sequencing with 150 bp read length was carried out on 73 RNA samples on HiSeq 4000 (Illumina Inc.) sequencer. The libraries were generated according to manufacturers’ protocol: Briefly, mRNA was isolated from 2 µg of total RNA using oligo-dT magnetic beads and fragmented at high temperature. A cDNA library was then prepared from the fragmented mRNA by reverse transcription, second strand synthesis and ligation of specific adapters. Next generation sequencing was performed on Illumina HiSeq 4000 (Illumina Inc.) with single-ended 150 read cycles. Image analysis and bases calling was conducted in real-time by the Illumina analysis pipeline.

Good quality reads were aligned to several human reference databases including mouse genome (build 10 mm), exon, splicing junction segment and contamination database including ribosome and mitochondrial sequences using Burrows Wheeler alignment algorithm (Li and Durbin 2009). After filtering, the reads that were uniquely aligned to the exon and splicing-junction segments with a maximum of 2 mismatches for each transcript were then counted for each corresponding transcript. The genes with a read count of less than 100 across all samples were excluded. The read counts of remaining genes were log2 transformed and normalized at an equal global median value.

Both microarray analysis and RNASeq are validated methods for transcriptomic profiling with good concordance; RNASeq has a wider dynamic range and may identify more differentially expressed genes (Zhao et al. 2014; Rao et al. 2018). Before merging microarray and RNA sequencing data for downstream circadian rhythm analysis, the microarray and RNA sequencing data were each subjected to median center normalization. The data of the common genes for both microarray and RNA sequencing were merged and further normalized by quantile normalization approach. Finally, batch correction was performed between microarray and RNA sequencing data using ComBat (Johnson et al. 2007) to eliminate technical variation. These normalization steps minimized the technical variations as evident for the 15 samples with both microarray and RNA sequencing data (Additional file 1: Figure S1A, B), or all the samples (Additional file 1: Figure S1C, D) and provided more robust statistical significance (Pariollaud et al. 2018). Spearman’s correlation coefficients for the 15 samples that were run on both platforms are shown in Additional file 1: Figure S1E.

To identify patterns of circadian rhythm from 6-time intervals over a 24 h period, ANOVA test was first performed to identify differentially expressed genes across intervals at an adjusted *p* value of ≤ 0.05 (2319 genes in young mice and 860 genes in nephritic mice). The RAIN test was furthered performed to identify the genes with a circadian rhythm pattern (2262 genes in young mice and 755 genes in nephritic mice—Additional file 1: Figure S2A) (Thaben and Westermark 2014). The enriched biological functions of the genes with circadian rhythm pattern at different time intervals were determined using Reduce and Visualize Gene Ontology (REVIGO) algorithm (Supek et al. 2011). Inflammatory genes and genes that reflect injury of stromal cells (Bethunaickan et al. 2014; Berthier et al. 1950) did not manifest a circadian pattern of expression and differences between young and nephritic mice and between nephritic and remission mice confirmed the presence of renal injury and inflammation in the nephritic mice and the induction of remission (Additional file 1: Figure S2B, C).

### ELISA for Bmal1 and Per2

Snap frozen kidney was homogenized in RIPA buffer containing 1X protease inhibitor cocktail (Roche, Indianapolis, IN). After centrifugation at 19,000 *g* for 10 min at 4 °C, the supernatant was collected and the protein concentration was determined using a BCA protein assay. Fine adjustment of protein concentration was made based on the concentration of β-actin as determined by ELISA using 1:500 anti-β actin (Anti-beta Actin antibody ab8227, Abcam Cambridge, MA) in PBS to coat the plates and 1:1000 (HRP-conjugated beta Actin antibody, Proteintech, Rosemont, IL) as secondary antibody. Plates (Falcon Labware, Lincoln Park, NJ) were coated with antibodies to Bmal1 (NBP1-28802, Novus Biologics Centennial, CO) diluted at 1:500 or Per2 at 1:1000 (bs-3927R, Bioss Antibodies, Woburn, MA) in PBS overnight at 4 °C. After blocking with PBS/3% BSA, the plates were incubated with serial dilutions of kidney lysate starting at 1:3 dilution in PBS/1% BSA for 1 h at 37 °C, followed by incubation with either 1:100 anti-Bmal1 HRP (sc-365645, Santa Cruz Biotechnology, Dallas, TX) or anti-Per2 polyclonal antibody diluted at 1:5000 (AB5428P, Millipore, Burlington, MA) in PBS/1% BSA and then a 1:1 substrate solution of hydrogen peroxide and tetramethylbenzidine (R&D Systems, Minneapolis, MN) to generate the colorimetric reaction. The plates were quenched and immediately read on the plate reader at 405 nm. A liver lysate from a BMALtg mouse was run in serial dilution on each plate as a quantitation control. To confirm the validity of the ELISA, lysate from a BMAL1 deficient mouse (a gift from Dr. Garret FitzGerald, University of Pennsylvania) was used as a negative control for Bmal1. No background activity was observed in the BMAL1 deficient lysate. Because BMAL deficient mice have high levels of Per2, this lysate was also used as the positive control for titration of Per2 levels.

### Serum and urine analytes

Urine Na and K levels were measured by Jackson Laboratories (Bar Harbor, ME). Serum and urine glucose were measured using a glucometer (ThermoFisher Scientific, Parsippany-Troy Hills, NJ). To correct for variations in urine dilution between samples, ratios of Na/creatinine and K/creatinine were calculated (Lee et al. 2013; Dyer et al. 1998; Mann et al. 2012; Koo et al. 2015). Creatinine was measured using a chromagenic assay (ThermoFisher Scientific). Serum and urine aldosterone were measured using a commercial inhibition ELISA assay (ENZO Life Sciences, Farmingdale, NY, USA) according to the manufacturer's instructions. Urine pH was measured using a pH Micro Electrode (Fisher Scientific).

### Quantitative PCR

5ug of RNA was reverse-transcribed with SuperScript II reverse transcriptase (Invitrogen Life Technologies, Carlsbad, CA). Amplification was performed in triplicate using SYBR Green PCR Master Mix (Roche Diagnostics, Tucson, AZ) and specific primers in a LightCycler480 (Roche Diagnostics). Dissociation curve analysis confirmed amplification of one specific product per primer pair. PCR primers were as follows: Avpr1a Forward primer 5’ gggataccaatttcgtttgg 3’; Reverse primer 5’ aagccagtaacgccgtgat 3’; Bmal1 Set 1: Forward primer 5’ gccccaccgacctactct 3’; Reverse primer 5’ tgtctgtgtccatactttcttgg 3’; Set 2: Forward primer 5’ ccaagaaagtatggacacagacaaa 3’; Reverse primer 5’ gcattcttgatccttccttggt 3’; Clock Forward primer 5’ ccagtcagttggtccatcatt 3’; Clock Reverse primer 5’ tggctcctaactgagctgaaa 3’; Per1 Forward primer 5’ gcttcgtggacttgacacct 3’; Per1 Reverse primer 5’ tgctttagatcggcagtggt 3’; Per2 Set 1: Forward primer 5’ gcttcgtggacttgacacct 3’; Reverse primer 5’ tgctttagatcggcagtggt 3’; Per2 Set 2: Forward primer 5’ cagcacgctggcaaccttgaagtat 3’; Per2 Reverse primer 5’ cagggctggctctcactggacatta 3’; Per3: Forward primer 5’ cataccaggtgcccgaga 3’; Per3 Reverse primer 5’ gctgctgttccatgctctg 3’; Rev-erbα Forward primer 5’ cagcatgatcaggtcaatctgt 3’; Rev-erbα Reverse primer 5’ agcaaatcgtaccattaaaacctc 3’; Avpr2: Forward primer 5’ ctggtgtctaccacgtctgc3’; Avpr2 Reverse primer 5’ ggtctcggtcatccagtagc 3’.

### Statistics

Comparisons between groups were performed using 2-tailed Mann Whitney analysis for 2 group comparisons or Kruskal–Wallis one-way analysis of variance for ≥ 3 group comparisons. Circadian rhythmicity was evaluated using Cosinor analysis (Moškon 2020) https://cosinor.online/app/cosinorOutput.php. A *p* value less than 0.05 was considered statistically significant.

### Ethical approval information

The study was approved by the Feinstein Institute IACUC (Protocol 2015-031).

## Results

### Nephritic mice have a profound disturbance of diurnal variation of multiple renal genes

**Fig. 1:**
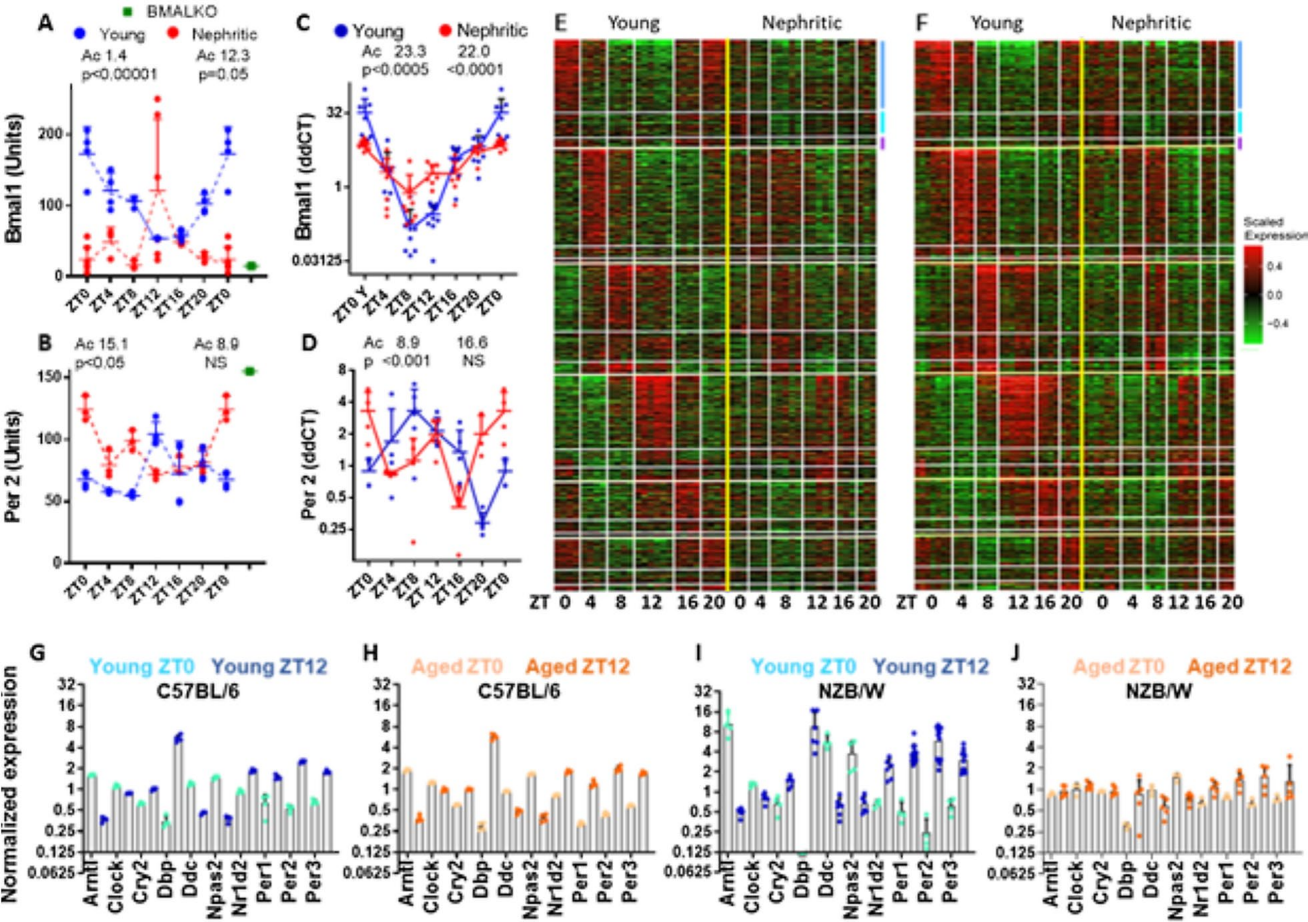
**A**, **B** Bmal1 and Per2 protein expression measured in renal lysates by ELISA (shown in dashed lines); **C**,** D** Bmal1 and Per2 expression measured in whole renal mRNA by qPCR (shown in solid lines)—each symbol represents one mouse;); *Ac * time of acrophase, *ZT* Zeitgeiber time where ZT0 is the time of lights on. **E** Heatmap of all genes passing the RAIN test for circadian rhythm in young and nephritic kidneys. Complete results from the microarray analysis are on the left and from the RNASeq analysis are on the right. For each time interval, indicated by Zeitgeiber time (ZT0 = lights on, ZT12 = lights off), genes regulated in young mice are shown in the upper panel (blue bar), those regulated in nephritic mice are shown in the middle panel (aqua bar) and those regulated in common between the two groups are shown in the lower panel (purple bar). **F**–**I** RNASeq data of circadian transcriptional regulators in young (light and dark blue—**F**, **H**) and old (light and dark orange—**G**, **I**) C57BL/6 mice at ZT0 (light colors) and ZT12 (dark colors) **F**, **G** compared with young (light and dark blue) and nephritic (light and dark orange) NZB/W kidneys **H**,** I**—each symbol represents one mouse

Diurnal expression of Bmal1and Per2 followed the normal pattern (Gachon et al. 2006; Zhang et al. 2014) in young NZB/W mice but was dysregulated in nephritic mice (Fig. 1A–D). Expression of other transcriptional regulators including *Per1, Per3, Clock* and, to a lesser extent, *Rev-erbα* (*Nr1d1*) and *Cry1* was also perturbed in the nephritic mice (Additional file 1: Figure S3A, B). Most circadian genes identified in young mice manifested a dampened pattern in the nephritic mice (Fig. 1E, F). Only 364 of 2653 diurnally regulated genes were shared between young and nephritic mice; of these 140 peaked at the same time and another 158 within 4 h with the rest being 8 or more hours off-cycle. 391 genes were expressed in a circadian fashion only in nephritic mice, but their circadian variation was generally of low amplitude (Additional file 1: Figure S3A, B).

To determine whether renal circadian disturbance is a general feature of aging, samples from 8 and 36- week-old C57BL/6 mice obtained at Zeitgeiber times (ZT) 0 and 12 were included in the RNASeq analysis. Expression of a panel of circadian regulatory genes whose diurnal variation was dampened in nephritic NZB/W mice was undisturbed in aged C57BL/6 mice (Fig. 1F–I).

To determine whether renal circadian disturbance is associated with disturbed sleep–wake cycles we observed mice over a 24 h period. Sleep–wake cycles followed a normal pattern in both young and nephritic NZB/W mice, however the nephritic mice were modestly more active during the light period and displayed diminished activity during the dark period compared with young mice (Additional file 1: Figure S3C, D).

### Pathways affected by circadian dysfunction in nephritic mice

**Fig. 2:**
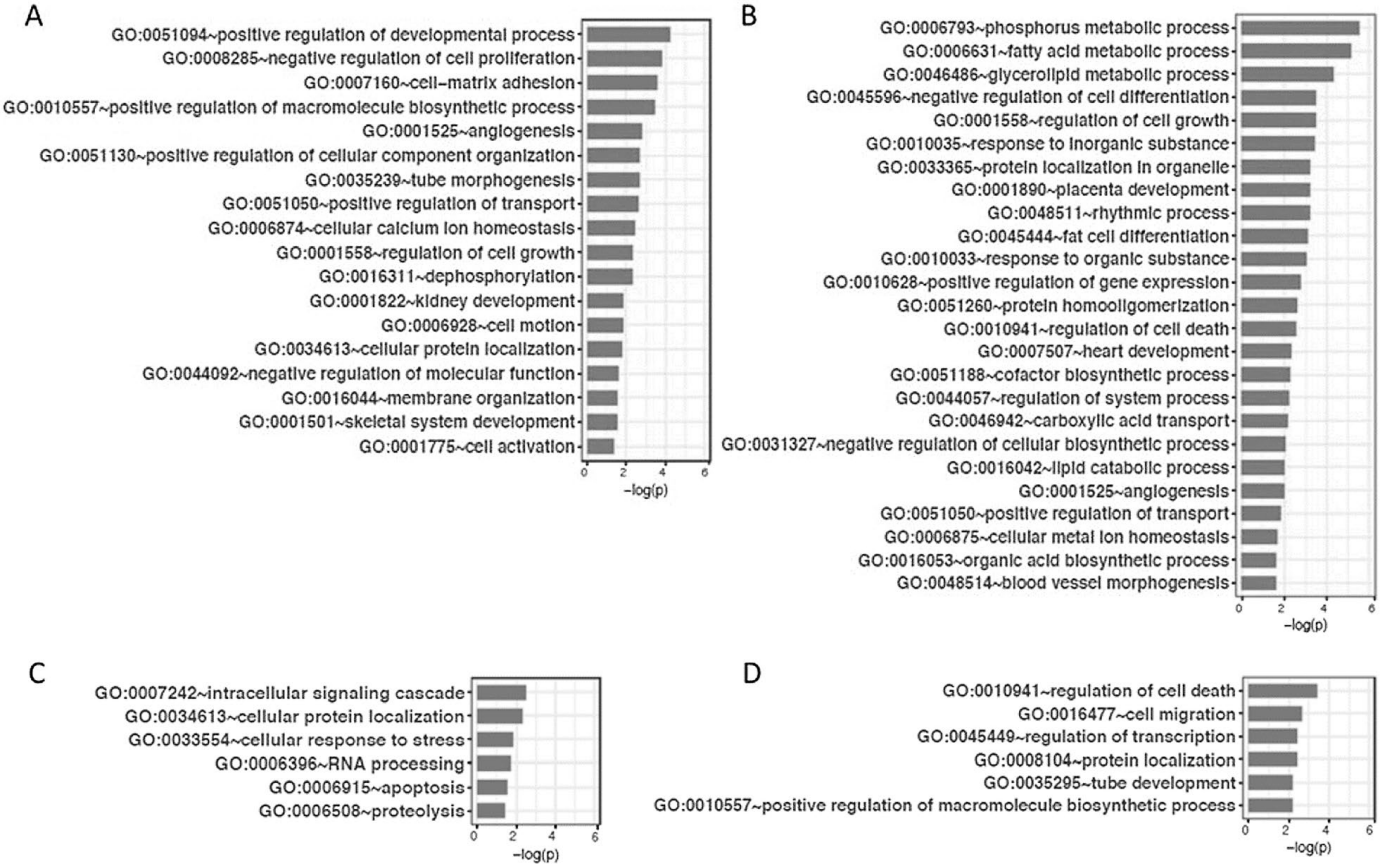
Renal functions that are regulated in a circadian fashion in NZB/W kidneys. **A** Young mice ZT0; **B** Young mice ZT12; **C** Nephritic mice ZT0; **D** Nephritic mice ZT12

To determine which functional pathways were perturbed in the kidneys of nephritic mice, Enriched Gene Functions were discovered at each time interval using REVIGO program (Fig. 2, Additional file 1: Figures S4A–F). In young mice, organ morphogenesis and cell growth predominated towards the end of the active phase at ZT20 and ZT0, whereas metabolic and cell transport processes predominated towards the end of the rest period at ZT8 and ZT12. By contrast, functional pathways in nephritic mice were dominated by processes related to protein modification and catabolism and responses to DNA damage.

**Fig. 3:**
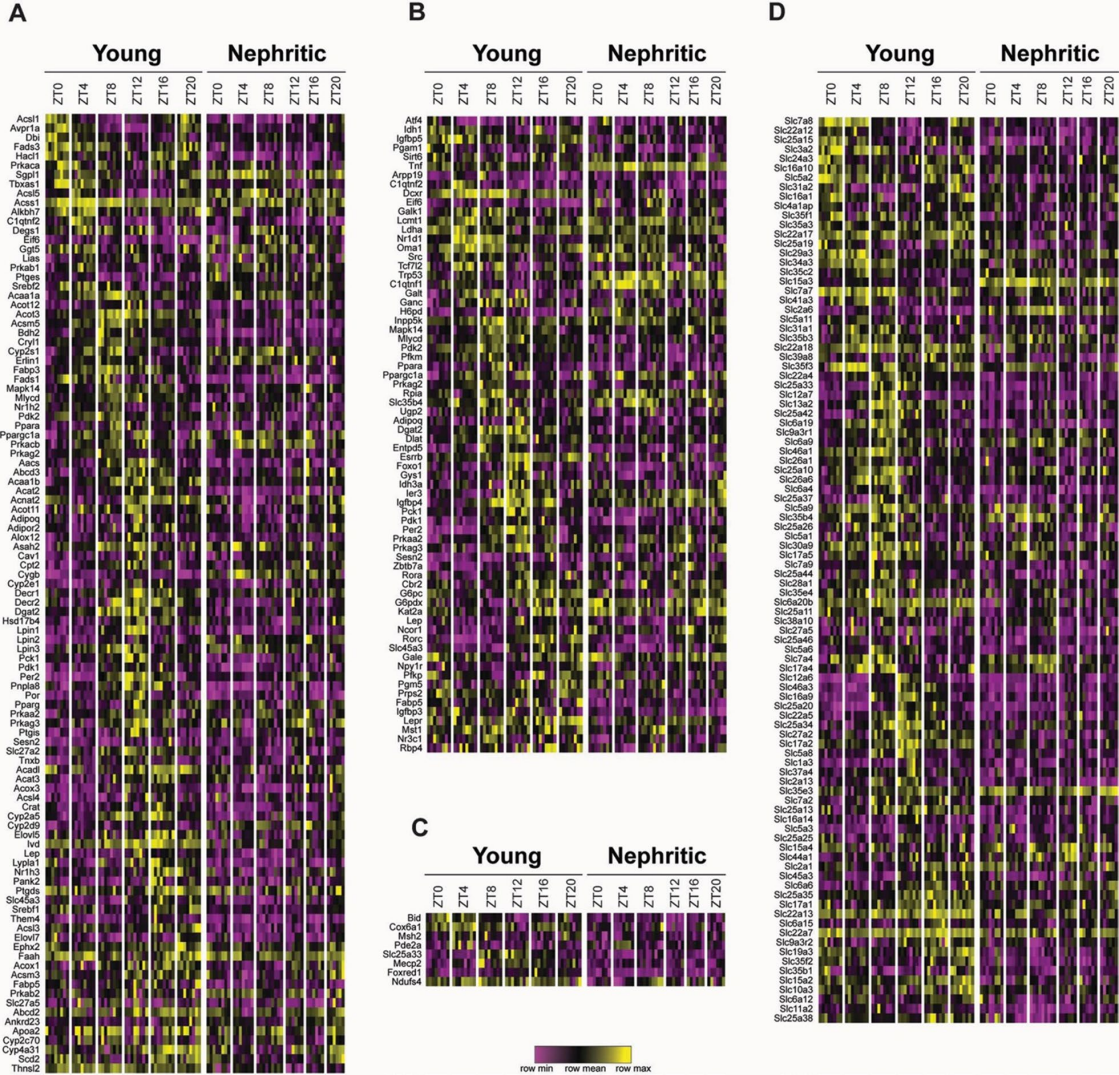
Heatmaps of genes involved in metabolic pathways in young and nephritic mice. **A** Fatty acid metabolism; **B** glycolysis; **C** Mitochondrial oxidation and respiration; **D** Slc family of transporters. Samples are the same as those shown in Additional file 1: Figure S2

Since abnormal metabolic processes are associated with CKD, we performed more detailed analysis of genes involved in metabolic functions (Additional file 1: Table S2). Many genes involved in fatty acid metabolism, particularly fatty acid synthesis and beta oxidation followed diurnal rhythms in young mice with peak expression at ZT8 and ZT12. By contrast, circadian oscillation of these genes was flattened in the nephritic mice (Fig. 3A). Heightened rhythmicity of the transcription factor *Srebp1* is associated with an increase in fatty acid synthesis in mice with fatty liver (Guan et al. 2018). By contrast, rhythmic expression of *Srebp1* was dampened in the kidneys of nephritic compared with young mice. In addition, overall expression levels of genes involved in fatty acid metabolism were lower in the nephritic mice than the young mice. These findings are consistent with the observed decrease in tubular fatty acid metabolism in mice with CKD (Kang et al. 2015).

Normal renal proximal tubule cells have low glycolytic activity which can increase as a consequence of renal tubular insult (Lan et al. 2016). The kidney is also responsible for ≈ 20% of total body gluconeogenesis. We noted robust circadian regulation of three enzymes involved in gluconeogenesis (*Idh1, G6pc* and *Pck1*) in young but not nephritic kidneys (Fig. 3B). By contrast, nephritic kidneys acquired an increase in expression and rhythmicity of both *G6pdx* and *H6pd*, enzymes that catalyze the first step of the pentose phosphate pathway (Fig. 3B). Only few genes involved in mitochondrial respiration were regulated in a circadian fashion even in young kidneys (Fig. 3C).

The Slc superfamily of solute carriers is a large family of membrane proteins that regulate ion fluxes and facilitate amino acid, sugar and nucleotide transport in and out of cells and cellular organelles. 85 Slc family members displayed diurnal variations in the young mice compared with 21 in the nephritic mice, of which only 10 overlapped between the two groups (Fig. 3D, Additional file 1: Table S3).

### Effect of circadian dysfunction on renal homeostatic functions

**Fig. 4:**
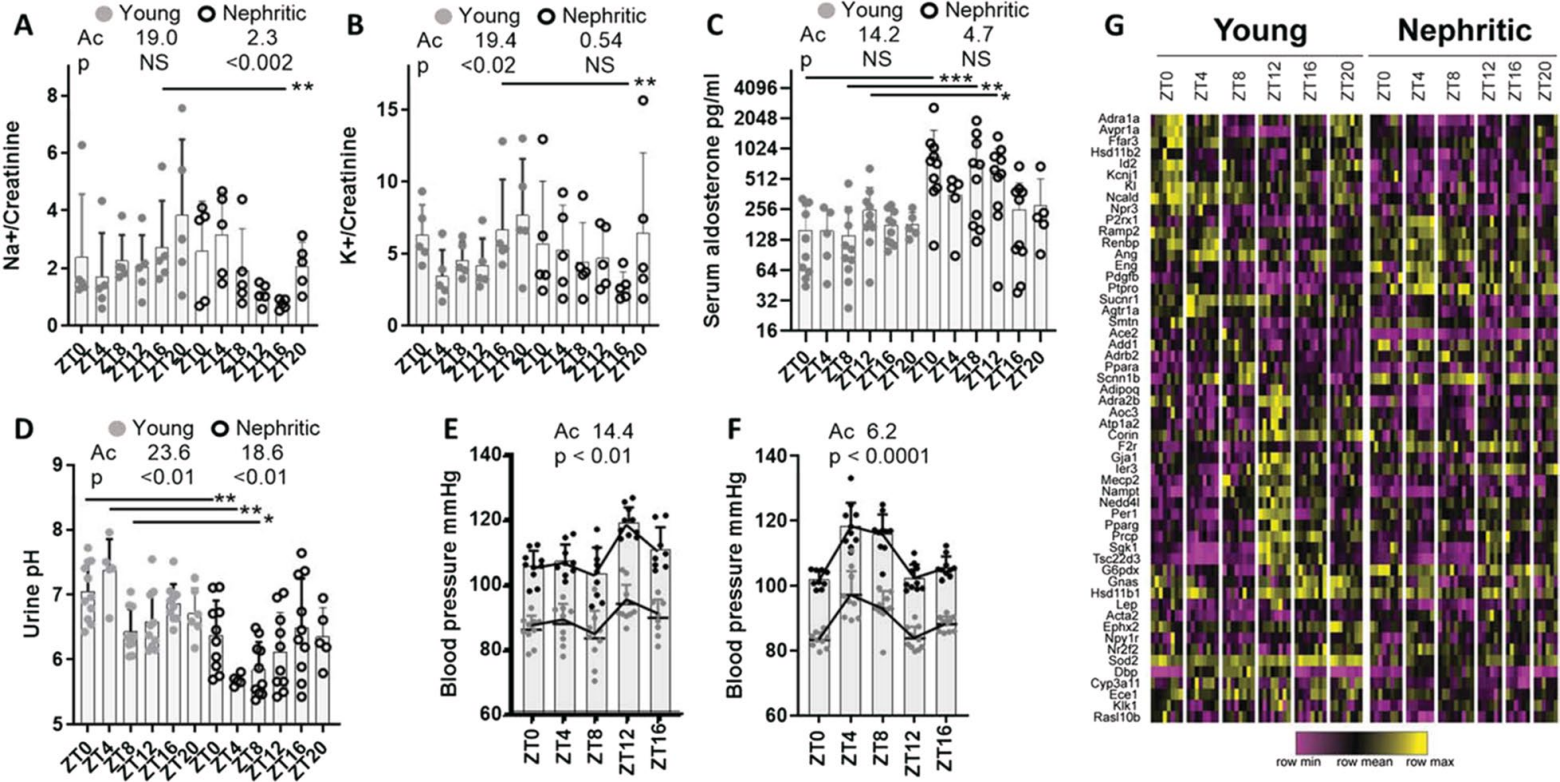
Dysregulated circadian regulation of renal salt excretion and blood pressure in nephritic mice **A** Urine Na + /Creatinine ratio; **B** Urine K + /Creatinine ratio Urine pH; **C** Serum aldosterone; **D** Urine pH; **E, F**: systolic (black) and diastolic (grey) blood pressure in young (**E**) and nephritic (**F**) mice shows reverse dipping pattern in nephritic mice;—each symbol represents one mouse. **G** Heatmap of genes that contribute to regulation of blood pressure. Samples are the same as those shown in Additional file 1: Figure S2. Rhythmicity was evaluated by Cosinor analysis (Ac = acrophase)

Diurnal variations of sodium and potassium excretion are regulated by oscillations of renal tubule transporters involved in water and ion transport (Douma and Gumz 2018; Gumz 2016). In young mice Na + and K + excretion was highest at ZT16 and ZT20 as expected (Nikolaeva et al. 2012). However, in nephritic mice the increase in excretion of both ions at the end of the active period did not occur (Fig. 4A, B). Similarly, the normal circadian variation in urine pH was dysregulated in nephritic mice and the urine pH was lower during the rest period than in young mice (Fig. 4C). There was no difference in urine creatinine between young and nephritic mice to account for these differences (Additional file 1: Figure S5).

Global deficiencies of some circadian transcriptional regulators are associated with a hypotensive and/or non-dipping blood pressure phenotype whereas others are associated with a hyperaldosterone state (Richards et al. 2013; Solocinski et al. 2017). Young mice displayed normal regulation of their blood pressure. By contrast, the nephritic mice displayed a reverse dipping pattern (Fig. 4D, E). Serum levels of aldosterone were highest at ZT12 in young mice as previously described (Nikolaeva et al. 2012). Nephritic mice, however, displayed high serum levels of aldosterone, especially during the day (Fig. 4F). This was associated with an increase in expression and circadian dysregulation of the β subunit of the epithelial sodium transporter (*Scnn1b)* in nephritic mice (Fig. 4G). Other genes involved in blood pressure control also demonstrated abnormal circadian regulation in nephritic mice. Expression of Angiotensin receptor 1a (*Agtr1*) and *Vegfc* was decreased throughout the day with a flattened rhythm, whereas expression of endothelin 1 (*Et1*), *Pdgfb*, *Adrb2, Cygb*, and *PtPro* was increased. Flattened rhythm of *Avpr1a, Cyp11b, Sgk1, Aqp3* and *Tsc22d3* (Gilz) was also observed in nephritic mice (Fig. 4G). These data in sum, suggest that in addition to the salt retention induced by the hyperaldosterone state, the disturbance of blood pressure dipping in the nephritic mice could be influenced by changes in expression of multiple renal genes that regulate blood pressure.

Glucose reabsorption in renal tubules is crucial for glucose homeostasis. We found robust circadian rhythm of the major proximal tubule glucose co-transporter *Slc5a2* (Sglt2) in young mice with a peak at ZT8 that was dampened in the nephritic mice. By contrast, there was an increase in expression of *Slc2a6* (Glut6) in the nephritic mice. Complete deficiency of Sglt2 has been associated with glycosuria (Ansary et al. 2019). Young mice fed ad libitum had a normal dip in serum blood glucose levels during the active period that was dampened in the nephritic mice. Nevertheless, serum and urine glucose levels remained within the physiologic range in both groups. (Additional file 1: Figure S6).

Several other transporter families, including amino acid transporters, L-carnitine transporters and mitochondrial transporters manifested diurnal regulation in young but not in nephritic mice (Fig. 3C, Additional file 1: Table S3). Of interest, *Slc25a33* and *Slc25a42* that provide substrates for mitochondrial oxidative phosphorylation and fatty acid oxidation respectively (Palmieri 2014), had a robust circadian regulation in young mice that was dampened in the nephritic mice.

### Induction of disease remission reverses circadian dysfunction

**Fig. 5:**
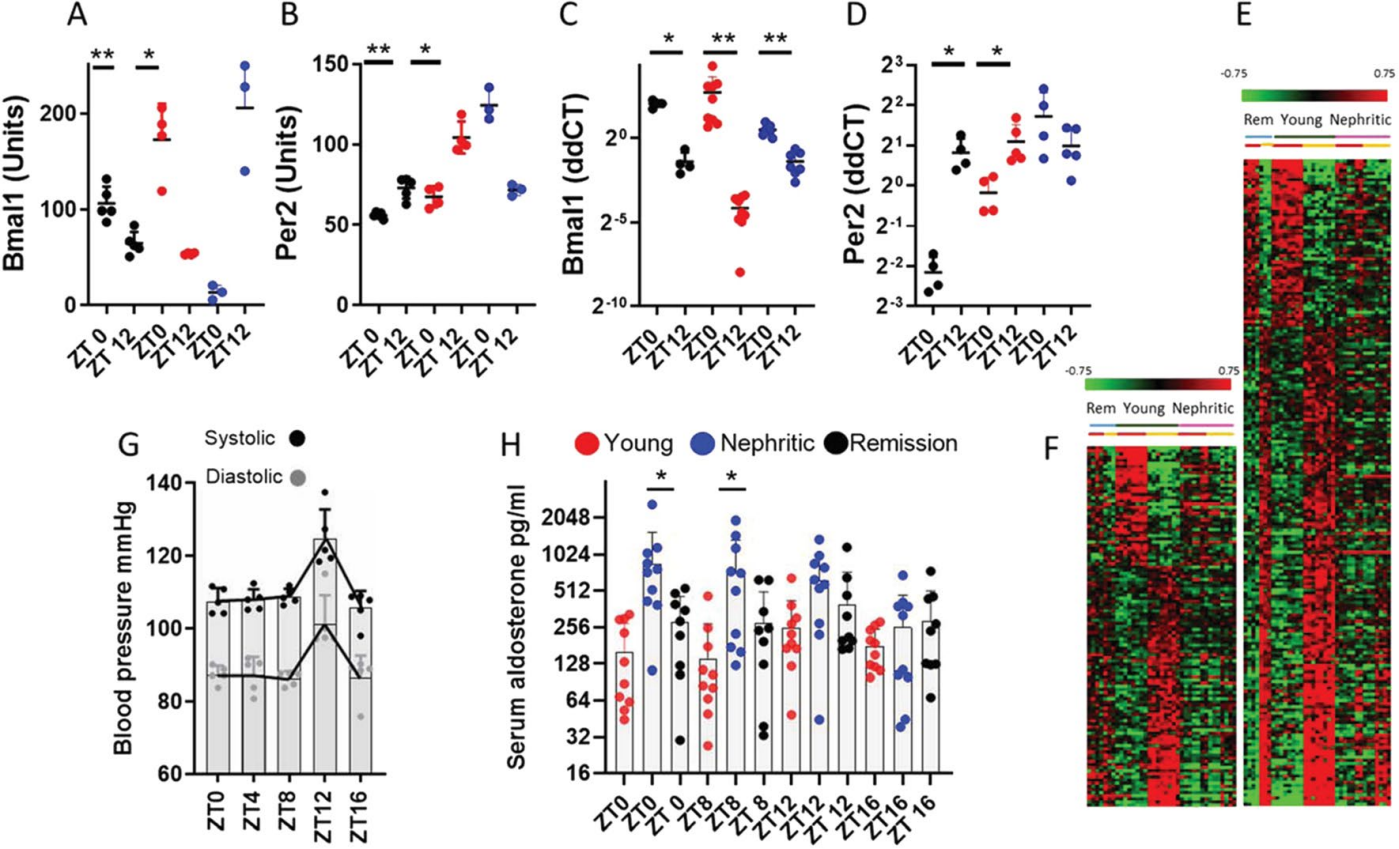
Partial correction of circadian dysfunction by remission induction therapy. **A, B** Bmal1 and Per2 protein expression measured in renal lysates by ELISA; **C**, **D** Bmal1 and Per2 expression measured in whole renal mRNA by qPCR; **E**, **F** Heatmap of 320 genes that differed between ZT0 (red bars) and ZT12 (Orange bars) in young but not nephritic kidneys showing correction of 205 genes (**E**) and failure to correct 115 genes (**F**). **G** Blood pressure in remission mice shows normalization of dipping pattern; **H** Correction of serum aldosterone in remission mice. *p < 0.05. Red symbols: young mice; Blue symbols: nephritic mice; Black symbols: remission mice—each symbol represents one mouse

To determine whether the chronodisruption of renal functions in nephritic NZB/W mice is reversible, we administered a combination of cyclophosphamide, CTLA4Ig and anti-CD40L to nephritic mice that developed fixed proteinuria of > 300 mg/dl. We have extensively reported that this combination induces robust clinical, histologic and molecular disease remission in ≈ 80% of nephritic NZB/W mice (Schiffer et al. 1950b; Bethunaickan et al. 2014). ELISA analysis of renal lysates from mice that maintained clinical remission (proteinuria ≤ 30 mg/dl) 8 weeks after induction with this regimen indicated restoration of the normal pattern of Bmal1 and Per2 expression at ZT0 and 12 (Fig. 5A–D). To determine whether this was associated with correction of diurnal variation of genes involved in renal homeostatic functions, we identified a panel of 320 diurnally regulated genes that had ≥ 25% difference in expression between times ZT0 and ZT12 in young mice (at an adjusted p value of < 0.05) and ≥ 25% difference in the ZT0:ZT12 ratio between young and nephritic mice. Of these genes, 148 showed at least 50% correction in the remission kidneys (Fig. 5E), and 172 did not correct (Fig. 5F, Additional file 1: Tables S4 and S5, Figure S7). Two weeks after the mice achieved remission of proteinuria, there was a reversal of the blood pressure abnormality in 5/5 mice, with normalization of the dipping pattern (Fig. 5G) and correction of the hyperaldosterone state (Fig. 5H).

## Discussion

Circadian dysregulation in CKD includes disturbed sleep, failure of blood pressure dipping and renal electrolyte abnormalities and it has significant health consequences, including an increase in cardiovascular risk. Little is currently known about the role of renal intrinsic dysregulation of circadian rhythm in causing these physiologic disruptions (Firsov and Bonny 2018; Carriazo et al. 2020). We report here a general dampening of renal circadian rhythm in lupus nephritis kidneys that can be partially reversed with remission inducing immune therapy. Inflammatory mediators such as TNF, IL1β and endotoxin suppress circadian genes in multiple cell types (Haimovich et al. 2009; Guo et al. 2015; Cavadini et al. 2007; Curtis and Fagundes 2015) and induce degradation of Rev-erbα in tissues (Pariollaud et al. 2018). TGFβ may also influence renal wound healing, and epithelial cell proliferation via the regulation of master clock regulators Per1 and Dec2 by Smad3 (Sato et al. 2019). In inflammatory bowel disease, expression of major clock transcriptional regulators is dampened in affected tissue; in this model, an agonist of the circadian regulator Rev-erbα, attenuated the disease (Wang 2018). Similarly, both Rev-erbα and Per2 agonists prevented severe injury of the heart in rodent models of cardiac ischemia reperfusion (Stujanna et al. 2017; Pourcet et al. 2018). Finally, there is increasing recognition that disturbance of peripheral organ clocks may cause feedback disruption to central clock functions (Carriazo et al. 2020; Myung et al. 2019). These findings, together with our new observations in the lupus kidney support the notion that local circadian disruption is a general feature of inflammation that contributes to organ dysfunction and that reversal of this process could be of therapeutic benefit.

Our study reveals that mice with active lupus nephritis have a reversal of the normal blood pressure dipping pattern. Global deficiency of *Bmal1, Clock* or *Per1* confers a hypotensive phenotype and/or changes in blood pressure dipping status, whereas deficiency of *Cry1/2* confers increased salt sensitivity and a non-dipping pattern (Doi et al. 2010); in the case of *Cry1/2*, there is also a hyperaldosterone state (Richards et al. 2013; Solocinski et al. 2017). Reverse dipping in humans is an extreme phenotype that occurs in patients with hypertension, type 2 diabetes mellitus and chronic kidney disease and is associated with cardiac dysfunction (Cuspidi et al. 2017; Tadic et al. 2020a, b). We show here that there are complex effects of nephritis on circadian regulation of renal genes that regulate blood pressure, with a hyperaldosterone state and increased expression of genes that regulate vasoconstriction. Other factors, including the effects of inflammation on systemic vascular function (Dinh et al. 2014; Savoia and Schiffrin 2006) could also contribute to the blood pressure abnormalities found in nephritic mice. Importantly, the reverse dipping blood pressure pattern in aged nephritic mice could be normalized by remission induction.

We also show circadian dysregulation of genes involved in renal metabolism. Renal tubular cells are dependent on fatty acid oxidation for their energy supply. We noted a significant dysregulation of diurnal variation of fatty acid transporters and of molecules involved in mitochondrial fatty acid oxidation and fatty acid synthesis. A defect in fatty acid metabolism in both mouse and human CKD and in nephritic NZB/W mice has previously been associated with a decrease in expression of the master transcriptional regulator Pparγc1α that regulates mitochondrial biogenesis and fatty acid oxidation (Kang et al. 2015). Our data suggests a more extensive defect in fatty acid synthesis and oxidation that is a major metabolic abnormality in the nephritic kidneys.

We also detected a decrease in diurnal variation of several genes involved in gluconeogenesis that reversed upon remission induction and an increase in the two rate limiting enzymes for the pentose phosphate pathway in the nephritic mice. A reduction in gluconeogenesis and an increase in renal hexokinase activity is induced by LPS in rodent kidneys perhaps reflecting an increased need for NADPH in the oxidatively stressed kidney (Smith et al. 2014) and/or provision of precursors for nucleotide synthesis that are important for cell growth (Lunt and Vander Heiden 2011). An induction of the pentose phosphate pathway has been observed in human inflammatory renal disease including LN and is correlated with inflammation (Grayson et al. 2018).

Another major role of kidney tubule transporters is to prevent the loss of amino acids in the urine (Makrides et al. 2014). We show here that there is a robust circadian regulation of transporters of the Slc6, Slc7/3 and Slc15 families in young NZB/W mice that is abrogated in the nephritic mice. Disturbances in regulation of amino acid transporters could contribute to tubular proteinuria in nephritic mice. We also observed disturbances in circadian regulation of organic anion/cation tubular transporters that could influence the renal toxicities of xenobiotics and other renally excreted drugs (Zhao et al. 2020).

One caveat of our studies is that it is difficult to control for the effects of age in NZB/W mice in which disease onset is stochastic and renal inflammation precedes the onset of proteinuria. Renal circadian dysregulation was not detected in aged non-autoimmune C57BL/6 mice. To further control for the effects of age we used kidneys from aged NZB/W mice from which collections were timed from complete responders after remission induction. We found that remission induction in aged NZB/W mice corrected the diurnal variation of more than 50% of the dysregulated genes. The effects of this treatment on blood pressure were striking, with concordance between complete remission of proteinuria and correction of the abnormal blood pressure dipper phenotype. This finding is of particular human relevance since nocturnal hypertension is an under-appreciated feature of active lupus nephritis that correlates with disease activity and interstitial damage and may be missed by day-time blood pressure measurements (Canpolat et al. 2013; Sabio et al. 2015). Ambulatory blood pressure monitoring (ABPM) is needed to detect such changes. Abnormal nocturnal blood pressure dipping is a major predictor of major adverse coronary events and its recognition and correction by timing of anti-hypertensive treatment attenuates cardiovascular morbidity and mortality as well as progression of CKD (Hermida et al. 2020). These findings suggest a need for ABPM in patients with LN and appropriate correction of abnormal blood pressure patterns. Further studies will be needed to understand the relevance of our findings to human LN and to determine whether reversal of nocturnal hypertension is a predictor for good renal outcome.

## Conclusions

Overall, our study reveals an underappreciated effect of renal inflammation on renal circadian rhythms in LN kidneys in NZB/W mice with multiple adverse effects on renal homeostasis and metabolism as well as blood pressure dipper status. Although we only used one LN mouse strain, similar disturbance of circadian master transcriptional regulators was observed in 2 other LN strains (Bethunaickan et al. 2013). Dampening of circadian rhythm in inflamed organs has recently been reported and agonists of the negative transcriptional regulators Rev-erbα and Per2 have broad anti-inflammatory functions and metabolic effects in vivo*,* especially when used before the onset of inflammation (Wang 2018). It is not yet clear whether this is due to modulation of local or systemic circadian circuits and the effects of such an approach in the setting of chronic inflammation have not yet been studied. We found modest disruption of renal diurnal rhythm of *Rev-erbα* and marked dysregulation of both *Per1* and *Per2* in the nephritic kidneys suggesting that these could be potential targets for circadian modulating therapy in lupus nephritis.

## Supplementary Information

**Additional file 1.** Additional tables and figures.

## Data Availability

Data has been submitted to GEO with the following Accession Number: GSE131894.

## Abbreviations

ZT: Zeitgeber time (ZT0 is lights on and ZT12 is lights off)
CKD: Chronic kidney disease
Slc: Solute carrier family
REVIGO: Reduce and visualize gene ontology
ABPM: Ambulatory blood pressure monitoring
