# Supplementary material for: Reversible dysregulation of renal circadian rhythm in lupus nephritis
Source: Mol Med. 2021 Sep 6;27:99. doi: 10.1186/s10020-021-00361-9 (PMC8419890; doi:10.1186/s10020-021-00361-9)
Supplement: Supplementary file 1 — Additional file 1. Additional tables and figures. [file 10020_2021_361_MOESM1_ESM.pdf]

SUPPLEMENTARY FIGURES AND TABLES

Supplementary Figure 1: A. Clustering of 15 samples with both microarray and RNA sequencing data before normalization; **B.** Clustering of 15 samples with both microarray and RNA sequencing data after median centering, quantile normalization and batch correction. The normalized data were segregated by the samples instead of experimental approaches; **C.** Clustering of all samples before normalization; **D.** Clustering of all samples with microarray and/or sequencing data after median centering, quantile normalization and batch correction shows clear separation between young and nephritic mice and between NZB/W and C57BL/6 strains. **E.** Correlation of microarray with RNASeq data for the 15 samples that were analyzed on both platforms.

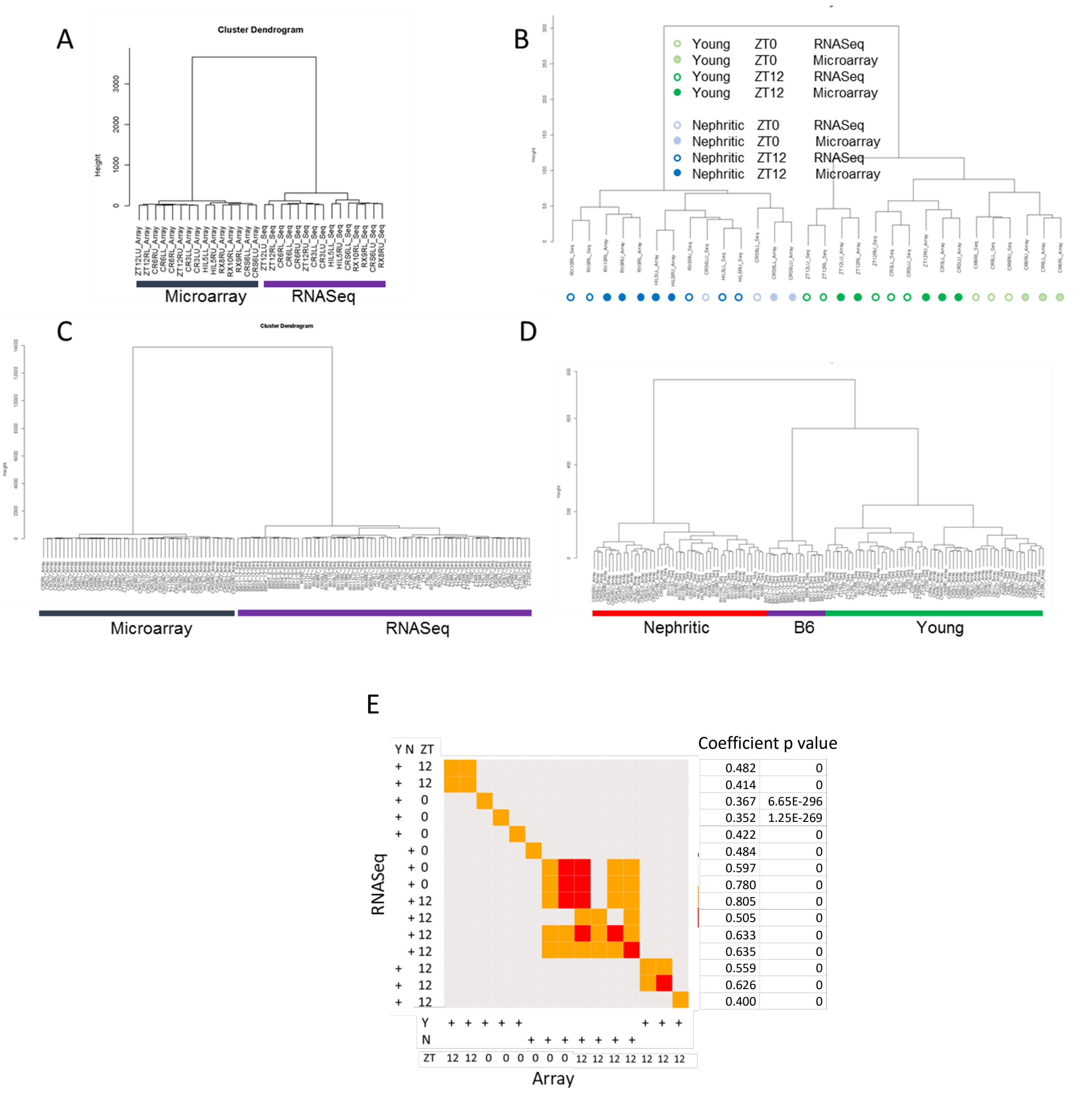

Supplementary Figure 2: **A:** Heat maps of genes that passed the RAIN test for circadian rhythm from young (upper panel) and nephritic (lower panel) NZB/W kidneys show the time of peak expression of each set of genes. Normalized data are shown and where samples were repeated only the sample analyzed by microarray analysis is shown. **B, C**: Expression of representative inflammatory genes (B) and genes reflecting injury of renal stromal cells (C) in young (grey symbols) and nephritic mice (black circles) and aged mice with induced remission (grey squares) confirm the clinical status of the mice. Statistics performed using Kruskal Wallis ANOVA followed by adjustment for multiple comparisons using Dunn's test. * p<0.05: ** p<0.01, *** p<0.001, **** p<0.0001.

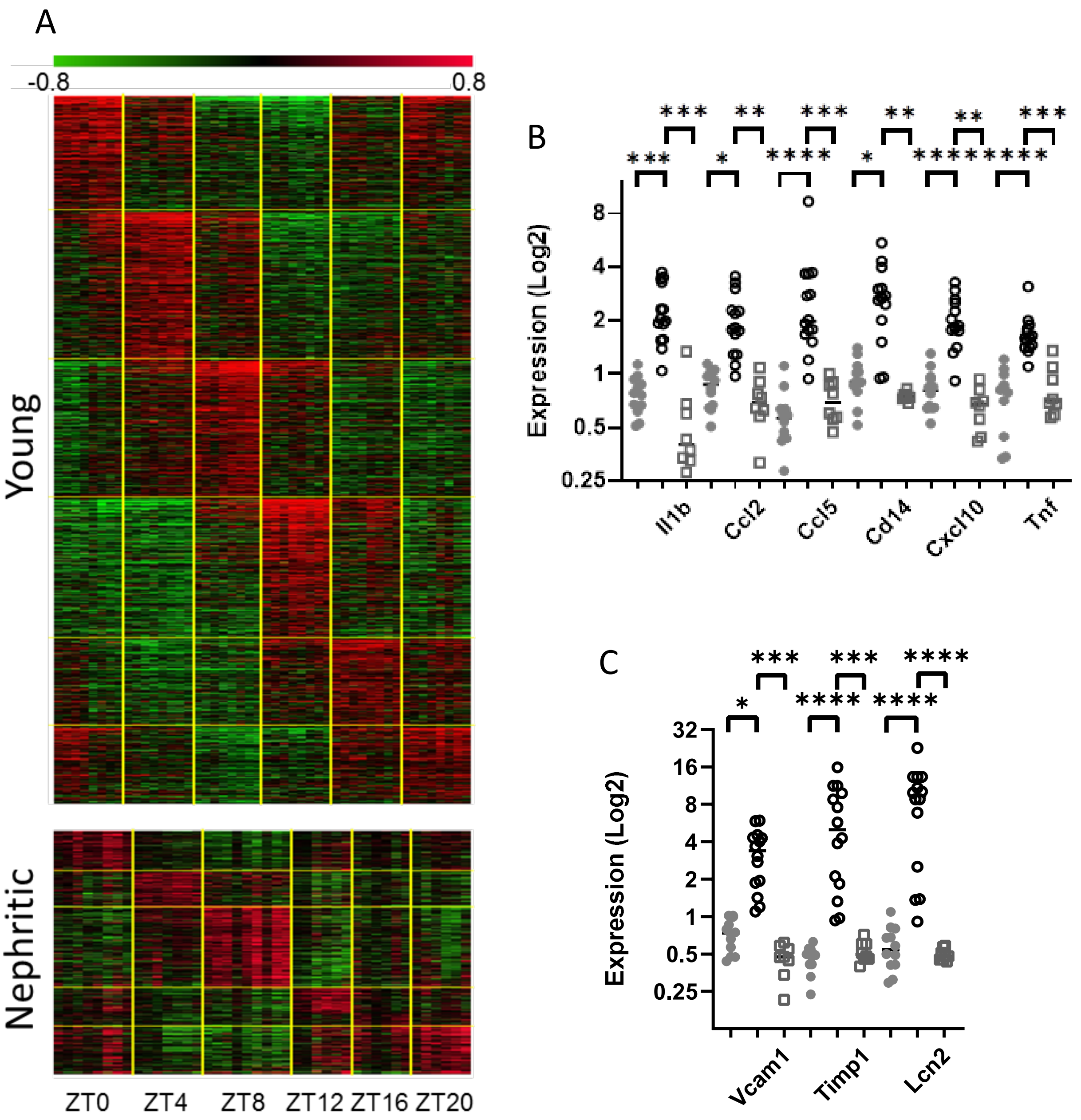

Supplementary Figure 3: A. PCR confirmation of a subset of genes, including several master transcriptional regulators of circadian rhythm, from young (black) and nephritic (grey) NZB/W kidneys. **B.** Heatmap of circadian genes in young and nephritic mice. Samples are the same as those shown in Figure 1. Each symbol represents an individual mouse. **C.** Sleep-wake cycles in young and nephritic mice observed over a 24 hour period. Data is displayed as the proportion of the time that the mice were active in each 10 minute-interval where 1=100%. D: Analysis of percent active time during the light and dark cycles. Statistics performed using Kruskal Wallis ANOVA followed by adjustment for multiple comparisons using Dunn's test. **** p<0.0001.

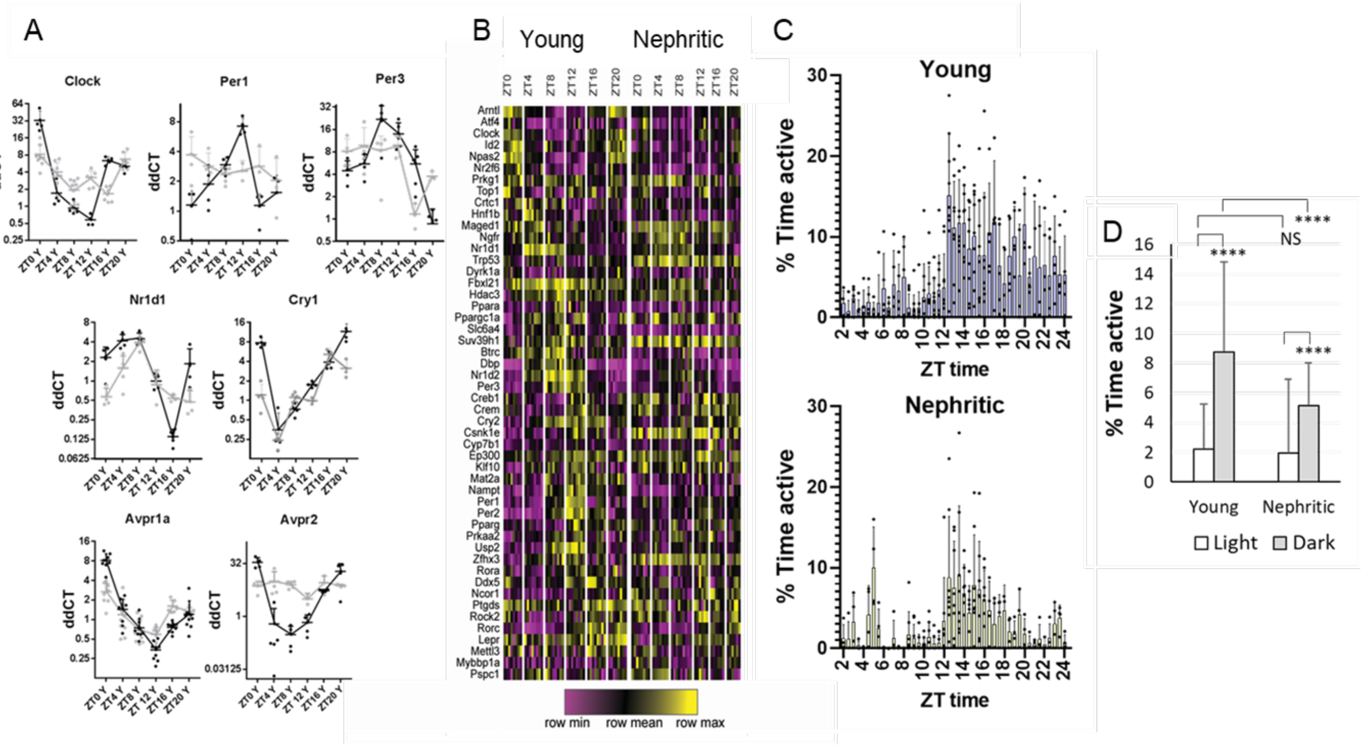

Supplementary Figure 4: REVIGO analyses of Genes passing the RAIN test for circadian rhythm in young (upper box in each panel) and nephritic (lower box in each panel) NZB/W kidneys at ZT0 (**A**), ZT4 (**B**), ZT8 (**C**), ZT12 (**D**), ZT16 (**E**) and ZT20 (**F**).

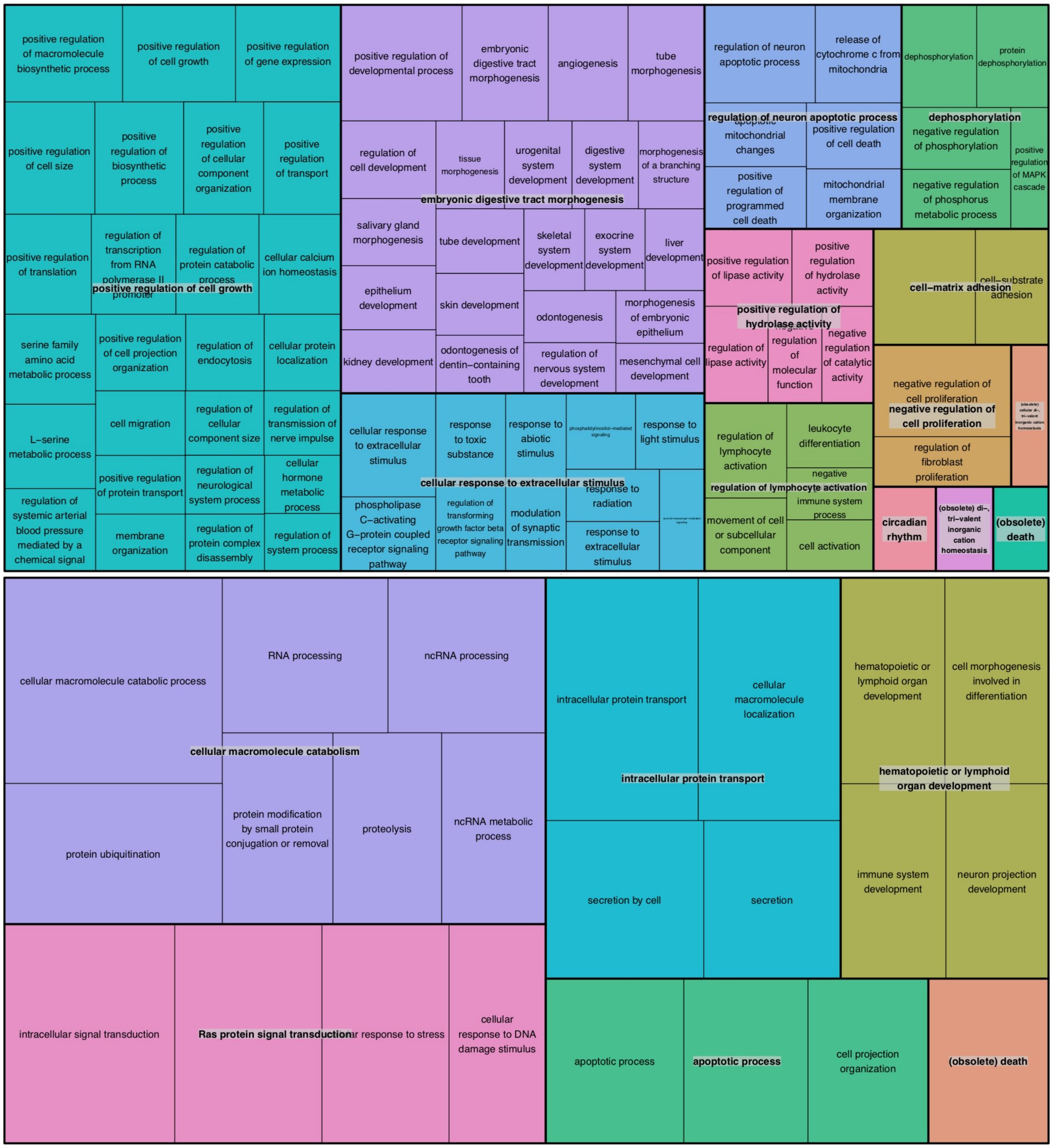

Supplementary Figure 4A: ZT0

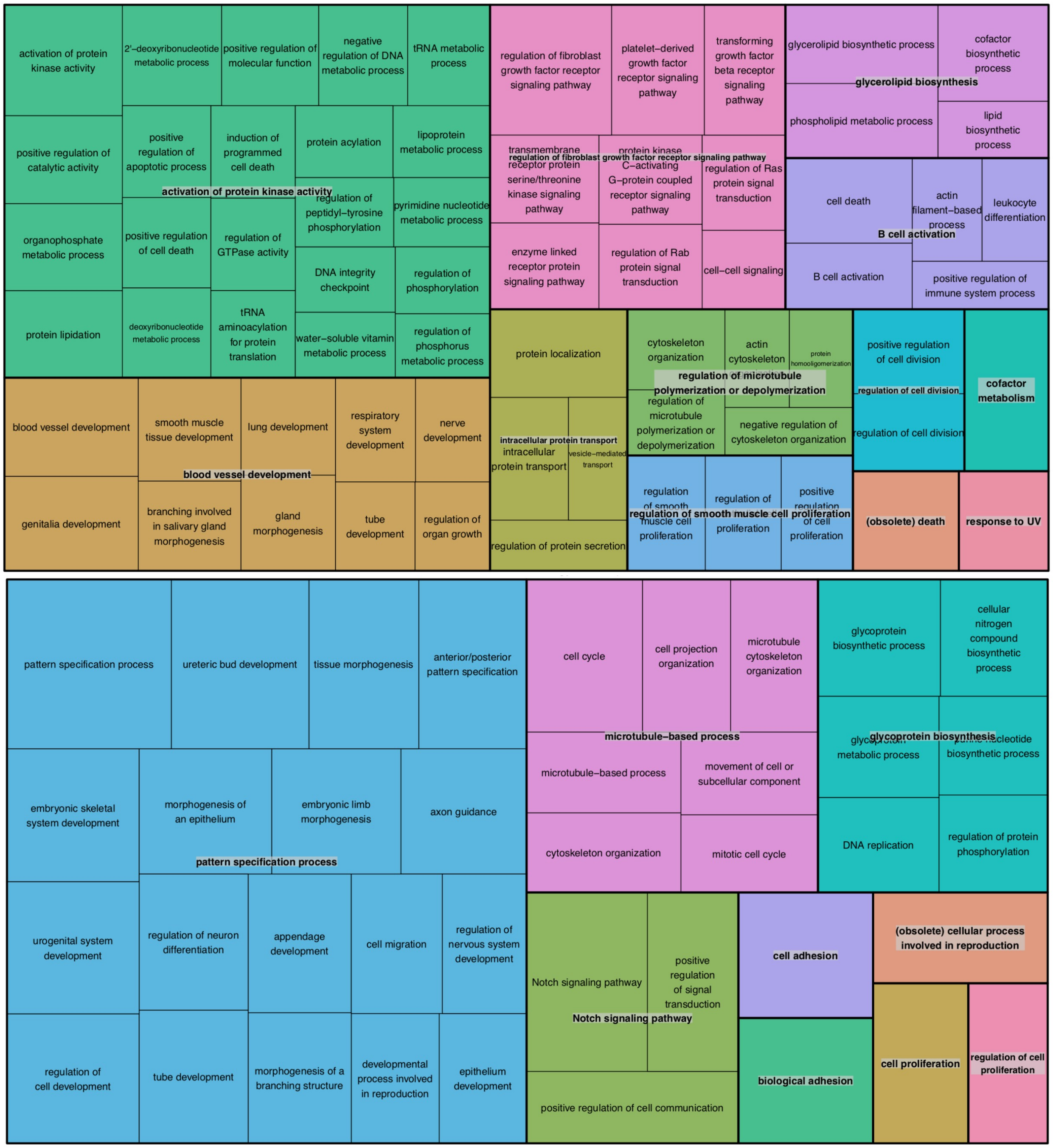

Supplementary Figure 4B: ZT4

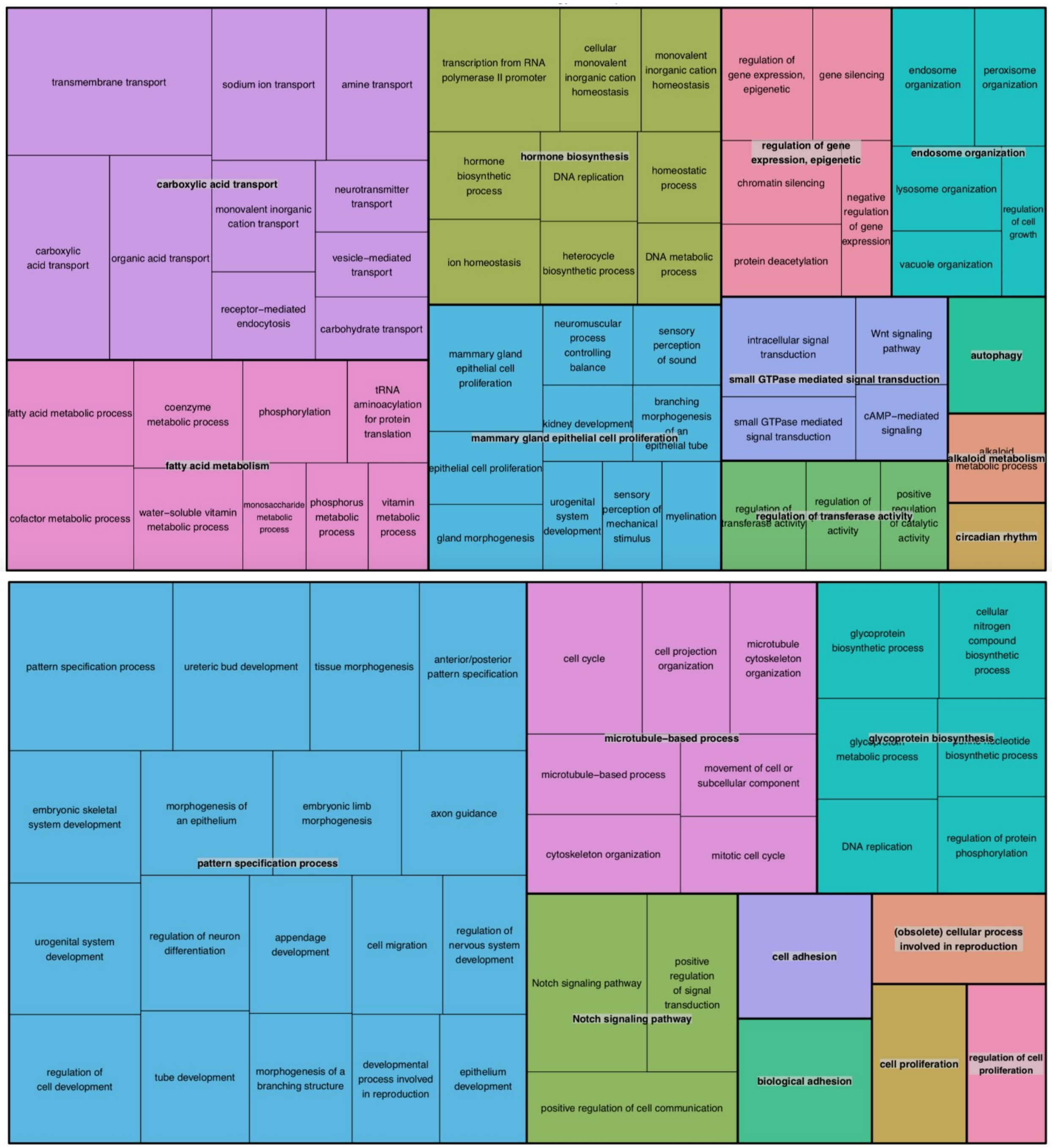

Supplementary Figure 4C: ZT8

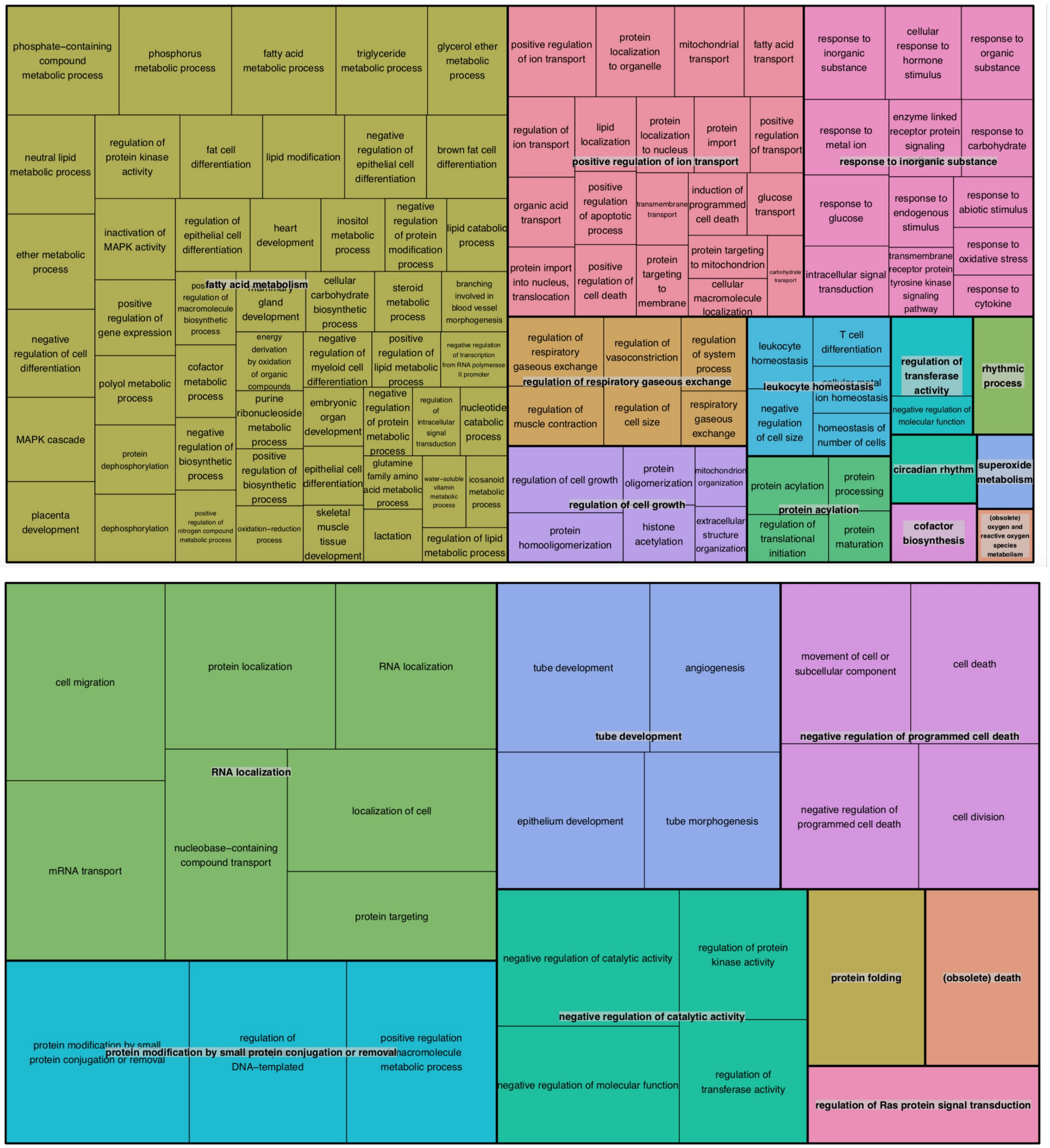

Supplementary Figure 4D: ZT12

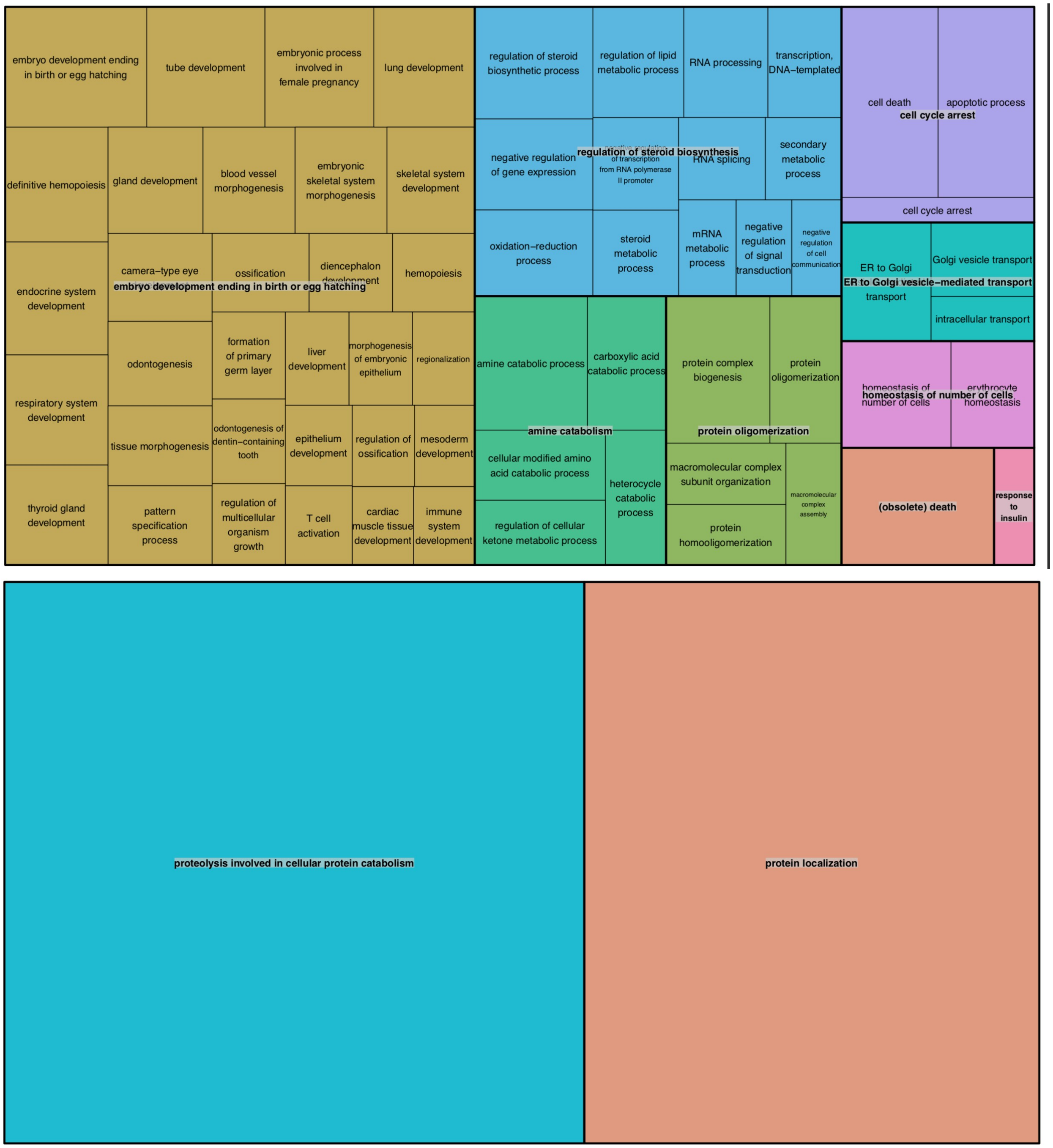

Supplementary Figure 4E: ZT16

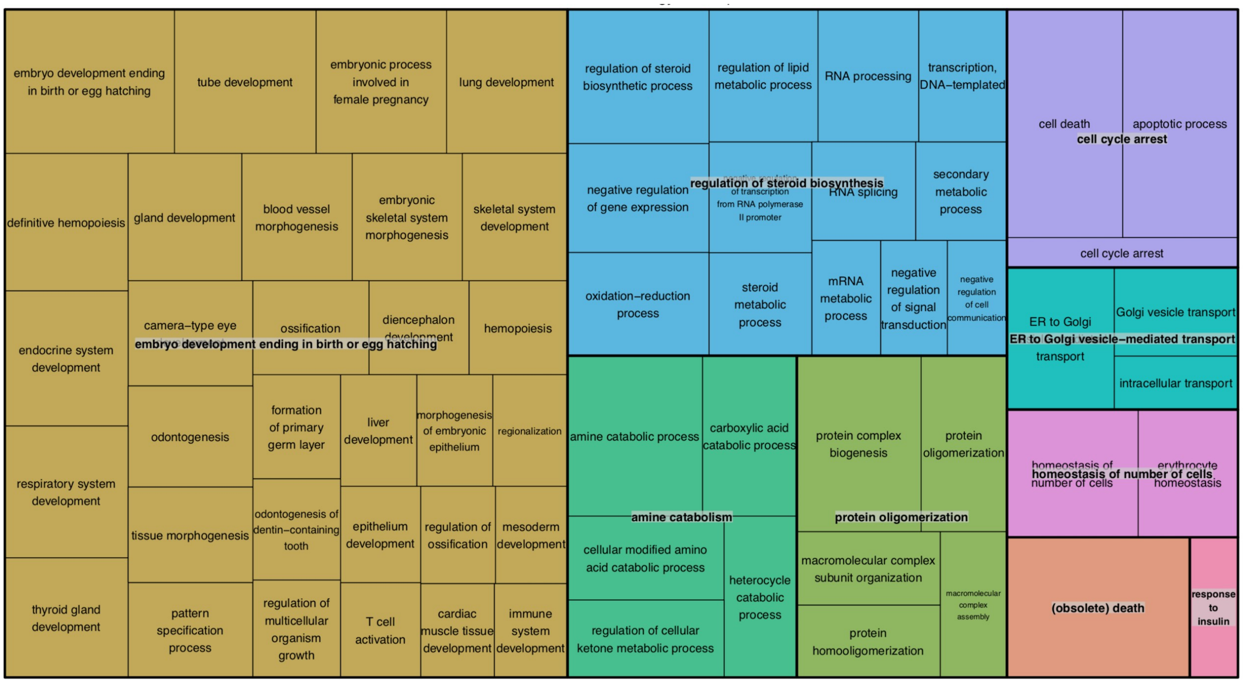

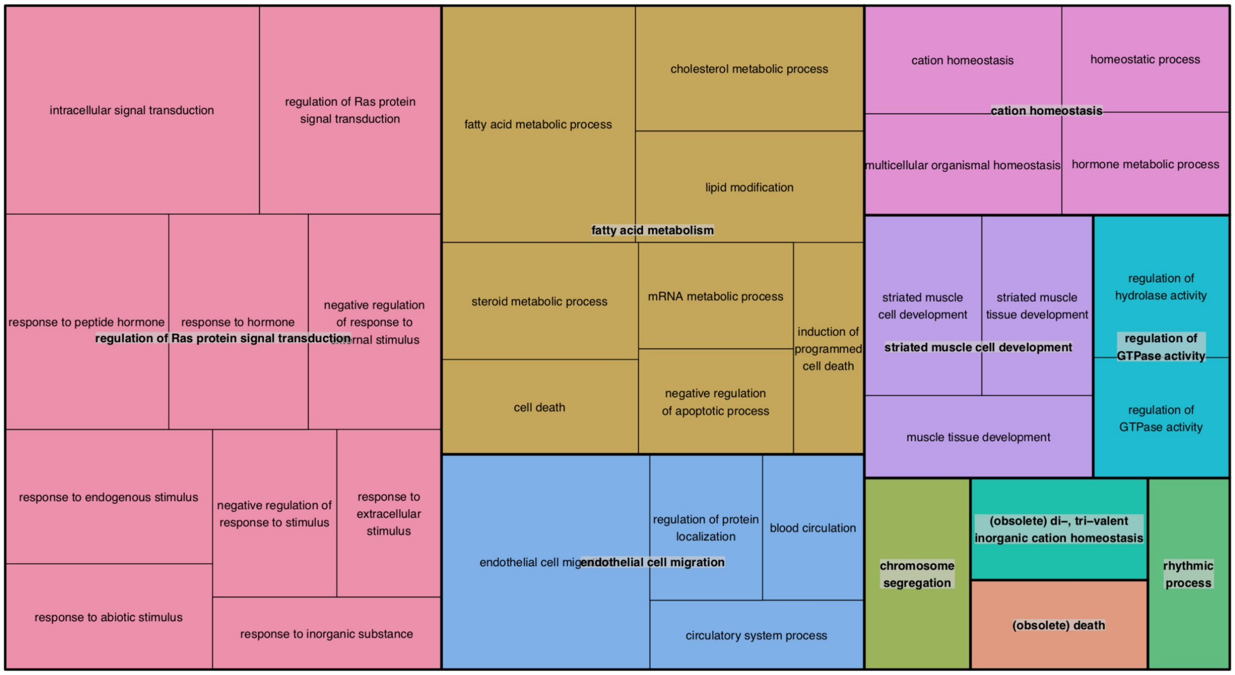

Supplementary Figure 4F: ZT20

Supplementary Figure 5: Urine sodium (A, B), potassium (C, D) and creatinine (E) in young (grey circles) and nephritic (black circles) NZB/W F1 mice. Each symbol represents an individual mouse. Statistics performed using Kruskal Wallis ANOVA followed by adjustment for multiple comparisons using Dunn's test. * $p<0.05$.

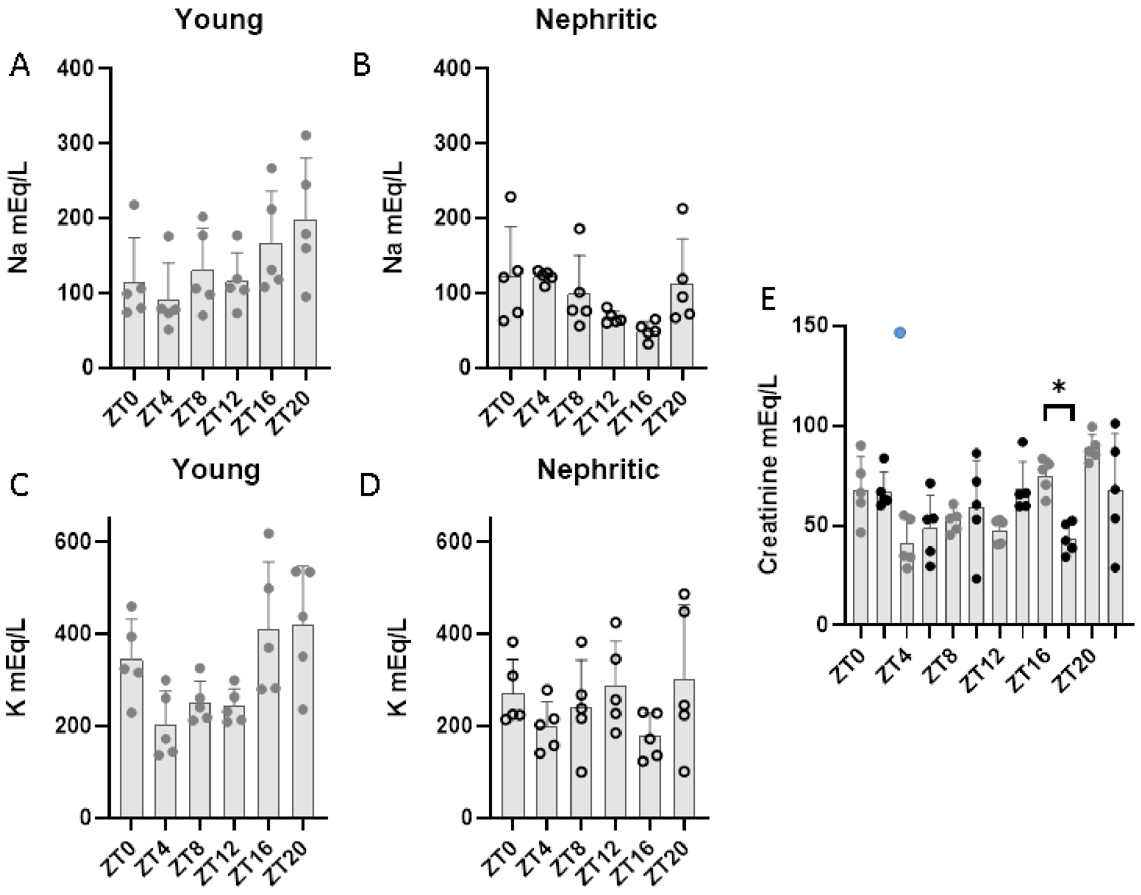

Supplementary Figure 6: Changes in serum glucose (**A, B**) and urine glucose (**C**) at the indicated ZT times in young (Y - grey) and nephritic (N – black circles) NZB/W mice.

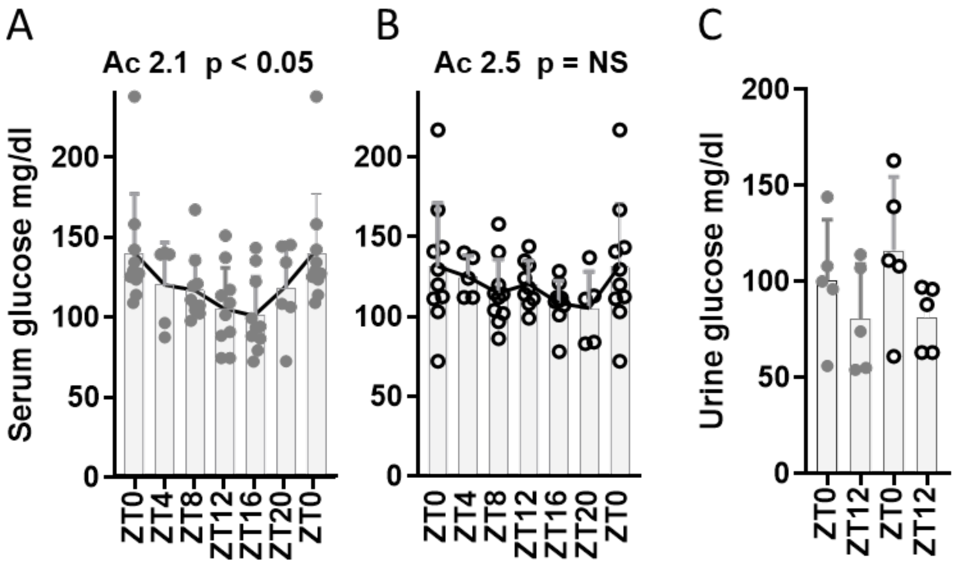

Supplementary Figure 7: Pathway analyses of circadian genes that corrected or did not correct after remission induction. A-C: Heatmaps of expression of genes related to circadian rhythm (**A**), fatty acid metabolism (**B**) and blood pressure regulation (**C**) from individual remission mice at ZT0 and ZT12 compared with young and nephritic mice. **D-G**. Major pathways identified by Ingenuity Pathway analysis that either corrected (green) or failed to correct (blue) after remission induction.

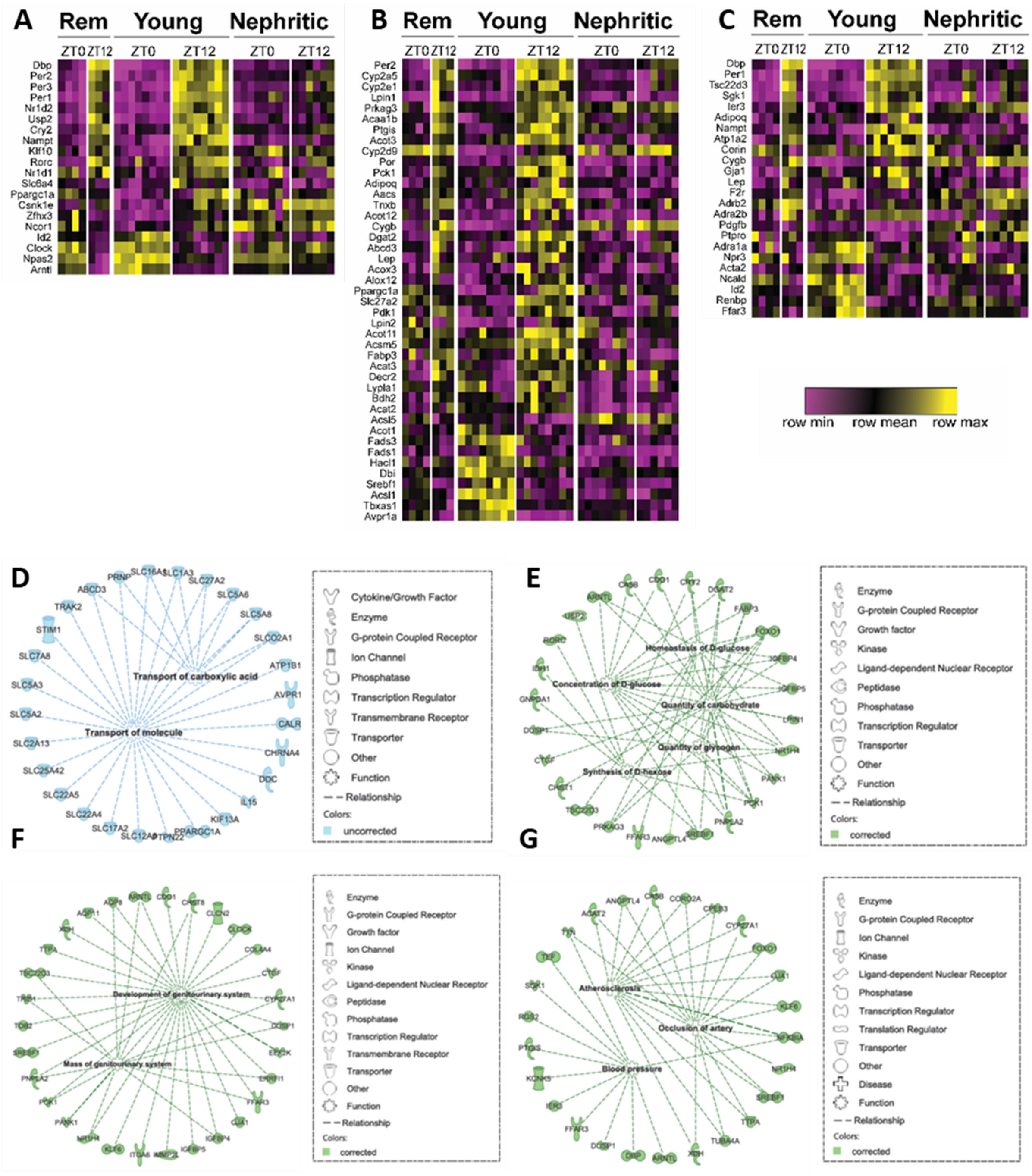

SUPPLEMENTARY TABLES

**Supplementary Table 1: Samples used for gene profiling**								
	**Time**	**Unique samples**	**Microarray**		**RNASeq**		**Both**	
			**Set 1**	**Set 2**	**Set 1**	**Set 2**	**Set 1**	**Set 2**
**NZB/W young**	ZT0	8	4		3	4	3	
	ZT4	8	4			4		
	ZT8	8	4			4		
	ZT12	7	3	3	3	4	2	3
	ZT16	8	4			4		
	ZT20	8	4			4		
**Total**		**47**	**23**			**24**		
**NZB/W Nephritic**	ZT0	7	3		3	4	2	
	ZT4	7	3			4		
	ZT8	9	5			4		
	ZT12	6	2	3	2	4	2	3
	ZT16	6	3			3		
	ZT20	6	3			3		
**Total**		**41**	**19**	**3**	**5**	**22**		
**C57BL/6 8 weeks**	ZT0	4				4		
	ZT12	4				4		
**C57BL/6 36 weeks**	ZT0	4				4		
	ZT12	4				4		
**Total**		**16**				**16**		
**NZB/W Remission**	ZT0	3				3		
	ZT12	4				4		
**Total**		**7**				**7**		

Supplementary Table 2: List of diurnally regulated genes involved in key metabolic and homeostatic functions				
**Category**	**Symbol**	**RefSeq**	**Name**	**UniGene_ID**
Blood Pressure	Avpr1a	NM_016847	arginine vasopressin receptor 1A	Mm.4351
Blood Pressure	Ncald	NM_001170867	neurocalcin delta	Mm.283370
Blood Pressure	Adra1a	NM_013461	adrenergic receptor, alpha 1a	Mm.57064
Blood Pressure	Npr3	NM_008728	natriuretic peptide receptor 3	Mm.25259
Blood Pressure	Renbp	NM_001164704	renin binding protein	Mm.236969
Blood Pressure	Id2	NM_010496	inhibitor of DNA binding 2	Mm.34871
Blood Pressure	Ramp2	NM_019444	receptor (calcitonin) activity modifying protein 2	Mm.260698
Blood Pressure	P2rx1	NM_008771	purinergic receptor P2X, ligand-gated ion channel, 1	Mm.25722
Blood Pressure	Hsd11b2	NM_008289	hydroxysteroid 11-beta dehydrogenase 2	Mm.5079
Blood Pressure	Kl	NM_013823	klotho	Mm.6500
Blood Pressure	Kcnj1	NM_001168354	potassium inwardly-rectifying channel, subfamily J, member 1	Mm.390168
Blood Pressure	Ffar3	NM_001033316	free fatty acid receptor 3	Mm.291167
Blood Pressure	Pdgfb	NM_011057	platelet derived growth factor, B polypeptide	Mm.144089
Blood Pressure	Ptpro	NM_011216	protein tyrosine phosphatase, receptor type, O	Mm.186361
Blood Pressure	Sucnr1	NM_032400	succinate receptor 1	Mm.125110
Blood Pressure	Ang	NM_001161731	angiogenin, ribonuclease, RNase A family, 5	Mm.202665
Blood Pressure	Eng	NM_007932	endoglin	Mm.225297
Blood Pressure	Ece1	NM_199307	endothelin converting enzyme 1	Mm.401062
Blood Pressure	P2rx1	NM_008771	purinergic receptor P2X, ligand-gated ion channel, 1	Mm.25722
Blood Pressure	Smtn	NM_001284428	smoothelin	Mm.188516
Blood Pressure	Agtr1a	NM_177322	angiotensin II receptor, type 1a	Mm.35062
Blood Pressure	Adrb2	NM_007420	adrenergic receptor, beta 2	Mm.5598
Blood Pressure	Scnn1b	NM_011325	sodium channel, nonvoltage-gated 1 beta	Mm.7709
Blood Pressure	Ace2	NM_027286	angiotensin I converting enzyme (peptidyl-dipeptidase A) 2	Mm.13451
Blood Pressure	Add1	NM_001331087	adducin 1 (alpha)	Mm.289106

Blood Pressure	Ppara	NM_001113418	peroxisome proliferator activated receptor alpha	Mm.212789
Blood Pressure	Sucnr1	NM_032400	succinate receptor 1	Mm.125110
Blood Pressure	Dbp	NM_016974	D site albumin promoter binding protein	Mm.24222
Blood Pressure	Tsc22d3	NM_010286	TSC22 domain family, member 3	Mm.22216
Blood Pressure	Sgk1	NM_001161848	serum/glucocorticoid regulated kinase 1	Mm.28405
Blood Pressure	Nampt	NM_021524	nicotinamide phosphoribosyltransferase	Mm.202727
Blood Pressure	Prcp	NM_028243	prolylcarboxypeptidase (angiotensinase C)	Mm.389969
Blood Pressure	Per1	NM_011065	period circadian clock 1	Mm.7373
Blood Pressure	Cygb	NM_030206	cytoglobin	Mm.34598
Blood Pressure	Gja1	NM_010288	gap junction protein, alpha 1	Mm.378921
Blood Pressure	Pparg	NM_001308352	peroxisome proliferator activated receptor gamma	Mm.3020
Blood Pressure	Mecp2	NM_010788	methyl CpG binding protein 2	Mm.131408
Blood Pressure	Nedd4l	NM_031881	neural precursor cell expressed, developmentally down-regulated gene 4-like	Mm.98668
Blood Pressure	Adra2b	NM_009633	adrenergic receptor, alpha 2b	Mm.347390
Blood Pressure	Ier3	NM_133662	immediate early response 3	Mm.25613
Blood Pressure	Corin	NM_001122756	corin	Mm.332425
Blood Pressure	Aoc3	NM_009675	amine oxidase, copper containing 3	Mm.67281
Blood Pressure	Atp1a2	NM_178405	ATPase, Na+/K+ transporting, alpha 2 polypeptide	Mm.207432
Blood Pressure	Adipoq	NM_009605	adiponectin, C1Q and collagen domain containing	Mm.3969
Blood Pressure	F2r	NM_010169	coagulation factor II (thrombin) receptor	Mm.24816
Blood Pressure	Klk1	NM_001320331	kallikrein 1	Mm.142722
Blood Pressure	Lep	NM_008493	leptin	Mm.277072
Blood Pressure	G6pdx	NM_008062	glucose-6-phosphate dehydrogenase X-linked	Mm.27210
Blood Pressure	Gnas	NM_001077510	GNAS (guanine nucleotide binding protein, alpha stimulating) complex locus	Mm.125770
Blood Pressure	Hsd11b1	NM_008288	hydroxysteroid 11-beta dehydrogenase 1	Mm.28328

Blood Pressure	Sod2	NM_013671	superoxide dismutase 2, mitochondrial	Mm.290876
Blood Pressure	Npy1r	NM_010934	neuropeptide Y receptor Y1	Mm.5112
Blood Pressure	Acta2	NM_007392	actin, alpha 2, smooth muscle, aorta	Mm.213025
Blood Pressure	Ephx2	NM_001271403	epoxide hydrolase 2, cytoplasmic	Mm.15295
Blood Pressure	Nr2f2	NM_183261	nuclear receptor subfamily 2, group F, member 2	Mm.158143
Blood Pressure	Adra1a	NM_013461	adrenergic receptor, alpha 1a	Mm.57064
Blood Pressure	Lep	NM_008493	leptin	Mm.277072
Blood Pressure	Sgk1	NM_001161848	serum/glucocorticoid regulated kinase 1	Mm.28405
Blood Pressure	Avpr1a	NM_016847	arginine vasopressin receptor 1A	Mm.4351
Blood Pressure	Tsc22d3	NM_010286	TSC22 domain family, member 3	Mm.22216
Blood Pressure	Rasl10b	NM_001013386	RAS-like, family 10, member B	Mm.66275
Blood Pressure	Cyp3a11	NM_007818	cytochrome P450, family 3, subfamily a, polypeptide 11	Mm.332844
Circadian Rhythm	Npas2	NM_008719	neuronal PAS domain protein 2	Mm.2380
Circadian Rhythm	Clock	NM_001289826	circadian locomotor output cycles kaput	Mm.3552
Circadian Rhythm	Arntl	NM_007489	aryl hydrocarbon receptor nuclear translocator-like	Mm.33970
Circadian Rhythm	Nr2f6	NM_010150	nuclear receptor subfamily 2, group F, member 6	Mm.28989
Circadian Rhythm	Id2	NM_010496	inhibitor of DNA binding 2	Mm.34871
Circadian Rhythm	Atf4	NM_009716	activating transcription factor 4	Mm.641
Circadian Rhythm	Prkg1	NM_011160	protein kinase, cGMP-dependent, type I	Mm.381170
Circadian Rhythm	Top1	NM_009408	topoisomerase (DNA) I	Mm.217233
Circadian Rhythm	Nr1d1	NM_145434	nuclear receptor subfamily 1, group D, member 1	Mm.390397
Circadian Rhythm	Maged1	NM_019791	melanoma antigen, family D, 1	Mm.27578
Circadian Rhythm	Hnf1b	NM_001291268	HNF1 homeobox B	Mm.7226
Circadian Rhythm	Ngfr	NM_033217	nerve growth factor receptor (TNFR superfamily, member 16)	Mm.283893
Circadian Rhythm	Crtc1	NM_001004062	CREB regulated transcription coactivator 1	Mm.227767

Circadian Rhythm	Trp53	NM_001127233	transformation related protein 53	Mm.222
Circadian Rhythm	Pspc1	NM_025682	paraspeckle protein 1	Mm.20129
Circadian Rhythm	Slc6a4	NM_010484	solute carrier family 6 (neurotransmitter transporter, serotonin), member 4	Mm.300318
Circadian Rhythm	Fbxl21	NM_178674	F-box and leucine-rich repeat protein 21	Mm.233904
Circadian Rhythm	Hdac3	NM_010411	histone deacetylase 3	Mm.20521
Circadian Rhythm	Ppara	NM_001113418	peroxisome proliferator activated receptor alpha	Mm.212789
Circadian Rhythm	Suv39h1	NM_011514	suppressor of variegation 3-9 homolog 1 (Drosophila)	Mm.9244
Circadian Rhythm	Ppargc1a	NR_132764	peroxisome proliferative activated receptor, gamma, coactivator 1 alpha	Mm.259072
Circadian Rhythm	Nr1d1	NM_145434	nuclear receptor subfamily 1, group D, member 1	Mm.390397
Circadian Rhythm	Nr1d2	NM_011584	nuclear receptor subfamily 1, group D, member 2	Mm.26587
Circadian Rhythm	Per3	NM_011067	period circadian clock 3	Mm.121361
Circadian Rhythm	Dbp	NM_016974	D site albumin promoter binding protein	Mm.24222
Circadian Rhythm	Btrc	NM_001286466	beta-transducin repeat containing protein	Mm.119717
Circadian Rhythm	Nampt	NM_021524	nicotinamide phosphoribosyltransferase	Mm.202727
Circadian Rhythm	Per2	NM_011066	period circadian clock 2	Mm.482463
Circadian Rhythm	Usp2	NM_198092	ubiquitin specific peptidase 2	Mm.272770
Circadian Rhythm	Cry2	NM_009963	cryptochrome 2 (photolyase-like)	Mm.254181
Circadian Rhythm	Csnk1e	NM_001289899	casein kinase 1, epsilon	Mm.30199
Circadian Rhythm	Per1	NM_011065	period circadian clock 1	Mm.7373
Circadian Rhythm	Klf10	NM_013692	Kruppel-like factor 10	Mm.4292
Circadian Rhythm	Crem	NM_001110854	cAMP responsive element modulator	Mm.5244

Circadian Rhythm	Pparg	NM_001308352	peroxisome proliferator activated receptor gamma	Mm.3020
Circadian Rhythm	Zfhx3	NM_007496	zinc finger homeobox 3	Mm.416972
Circadian Rhythm	Ep300	NM_177821	E1A binding protein p300	Mm.258397
Circadian Rhythm	Prkaa2	NM_178143	protein kinase, AMP-activated, alpha 2 catalytic subunit	Mm.48638
Circadian Rhythm	Mat2a	NM_145569	methionine adenosyltransferase II, alpha	Mm.29815
Circadian Rhythm	Cyp7b1	NM_007825	cytochrome P450, family 7, subfamily b, polypeptide 1	Mm.316000
Circadian Rhythm	Creb1	NM_001037726	cAMP responsive element binding protein 1	Mm.422634
Circadian Rhythm	Lepr	NM_001122899	leptin receptor	Mm.259282
Circadian Rhythm	Clock	NM_001289826	circadian locomotor output cycles kaput	Mm.3552
Circadian Rhythm	Rock2	NM_009072	Rho-associated coiled-coil containing protein kinase 2	Mm.276024
Circadian Rhythm	Rora	NM_013646	RAR-related orphan receptor alpha	Mm.378450
Circadian Rhythm	Rorc	NM_011281	RAR-related orphan receptor gamma	Mm.4372
Circadian Rhythm	Ddx5	NM_007840	DEAD (Asp-Glu-Ala-Asp) box polypeptide 5	Mm.491096
Circadian Rhythm	Ncor1	NM_011308	nuclear receptor co-repressor 1	Mm.271814
Circadian Rhythm	Ptgds	NM_008963	prostaglandin D2 synthase (brain)	Mm.1008
Circadian Rhythm	Rock2	NM_009072	Rho-associated coiled-coil containing protein kinase 2	Mm.276024
Circadian Rhythm	Dyrk1a	NM_007890	dual-specificity tyrosine-(Y)-phosphorylation regulated kinase 1a	Mm.310973
Circadian Rhythm	Rorc	NM_011281	RAR-related orphan receptor gamma	Mm.4372
Circadian Rhythm	Arntl	NM_007489	aryl hydrocarbon receptor nuclear translocator-like	Mm.33970
Circadian Rhythm	Npas2	NM_008719	neuronal PAS domain protein 2	Mm.2380
Circadian Rhythm	Mybbp1a	NM_016776	MYB binding protein (P160) 1a	Mm.147946

Circadian Rhythm	Mettl3	NM_019721	methyltransferase like 3	Mm.271759
Fatty Acid	Avpr1a	NM_016847	arginine vasopressin receptor 1A	Mm.4351
Fatty Acid	Acsl1	NM_007981	acyl-CoA synthetase long-chain family member 1	Mm.210323
Fatty Acid	Tbxas1	NM_011539	thromboxane A synthase 1, platelet	Mm.4054
Fatty Acid	Dbi	NM_007830	diazepam binding inhibitor	Mm.2785
Fatty Acid	Fads3	NM_021890	fatty acid desaturase 3	Mm.253875
Fatty Acid	Hacl1	NM_019975	2-hydroxyacyl-CoA lyase 1	Mm.38887
Fatty Acid	Sgpl1	NM_001316673	sphingosine phosphate lyase 1	Mm.412319
Fatty Acid	Prkaca	NM_001277898	protein kinase, cAMP dependent, catalytic, alpha	Mm.19111
Fatty Acid	Prkab2	NM_182997	protein kinase, AMP-activated, beta 2 non-catalytic subunit	Mm.31175
Fatty Acid	C1qtnf2	NM_026979	C1q and tumor necrosis factor related protein 2	Mm.390374
Fatty Acid	Acsl5	NM_027976	acyl-CoA synthetase long-chain family member 5	Mm.292056
Fatty Acid	Acss1	NM_080575	acyl-CoA synthetase short-chain family member 1	Mm.7044
Fatty Acid	Srebf2	NM_033218	sterol regulatory element binding factor 2	Mm.9002
Fatty Acid	Eif6	NM_010579	eukaryotic translation initiation factor 6	Mm.271674
Fatty Acid	Prkab1	NM_031869	protein kinase, AMP-activated, beta 1 non-catalytic subunit	Mm.458152
Fatty Acid	Ptges	NM_022415	prostaglandin E synthase	Mm.28768
Fatty Acid	Alkbh7	NM_025538	alkB homolog 7	Mm.196150
Fatty Acid	Lias	NM_001310612	lipoic acid synthetase	Mm.195776
Fatty Acid	Ggt5	NM_011820	gamma-glutamyltransferase 5	Mm.257927
Fatty Acid	Degs1	NM_007853	delta(4)-desaturase, sphingolipid 1	Mm.29648
Fatty Acid	Acaa1a	NM_130864	acetyl-Coenzyme A acyltransferase 1A	Mm.205266
Fatty Acid	Acsm5	NM_178758	acyl-CoA synthetase medium-chain family member 5	Mm.185183
Fatty Acid	Fabp3	NM_010174	fatty acid binding protein 3, muscle and heart	Mm.388886
Fatty Acid	Mapk14	NM_001168508	mitogen-activated protein kinase 14	Mm.311337

Fatty Acid	Cyp2s1	NM_028775	cytochrome P450, family 2, subfamily s, polypeptide 1	Mm.275188
Fatty Acid	Acot3	NM_134246	acyl-CoA thioesterase 3	Mm.202331
Fatty Acid	Prkag2	NM_001170556	protein kinase, AMP-activated, gamma 2 non-catalytic subunit	Mm.33649
Fatty Acid	Mlycd	NM_019966	malonyl-CoA decarboxylase	Mm.423037
Fatty Acid	Acot12	NM_028790	acyl-CoA thioesterase 12	Mm.275963
Fatty Acid	Bdh2	NM_027208	3-hydroxybutyrate dehydrogenase, type 2	Mm.45121
Fatty Acid	Cryl1	NM_030004	crystallin, lambda 1	Mm.25539
Fatty Acid	Prkacb	NM_001164198	protein kinase, cAMP dependent, catalytic, beta	Mm.16766
Fatty Acid	Nr1h2	NM_001285518	nuclear receptor subfamily 1, group H, member 2	Mm.968
Fatty Acid	Erlin1	NM_145502	ER lipid raft associated 1	Mm.279865
Fatty Acid	Ppara	NM_001113418	peroxisome proliferator activated receptor alpha	Mm.212789
Fatty Acid	Ppargc1a	NR_132764	peroxisome proliferative activated receptor, gamma, coactivator 1 alpha	Mm.259072
Fatty Acid	Pdk2	NM_133667	pyruvate dehydrogenase kinase, isoenzyme 2	Mm.29768
Fatty Acid	Fads1	NM_146094	fatty acid desaturase 1	Mm.30158
Fatty Acid	Degs1	NM_007853	delta(4)-desaturase, sphingolipid 1	Mm.29648
Fatty Acid	Tbxas1	NM_011539	thromboxane A synthase 1, platelet	Mm.4054
Fatty Acid	Fabp5	NM_001272097	fatty acid binding protein 5, epidermal	Mm.741
Fatty Acid	Thnsl2	NM_001033929	threonine synthase-like 2 (bacterial)	Mm.37547
Fatty Acid	Slc27a5	NM_009512	solute carrier family 27 (fatty acid transporter), member 5	Mm.10984
Fatty Acid	Cyp2c70	NM_145499	cytochrome P450, family 2, subfamily c, polypeptide 70	Mm.29119
Fatty Acid	Apoa2	NR_131173	apolipoprotein A-II	Mm.389209
Fatty Acid	Per2	NM_011066	period circadian clock 2	Mm.482463
Fatty Acid	Por	NM_008898	P450 (cytochrome) oxidoreductase	Mm.3863
Fatty Acid	Cyp2e1	NM_021282	cytochrome P450, family 2, subfamily e, polypeptide 1	Mm.21758
Fatty Acid	Cpt2	NM_009949	carnitine palmitoyltransferase 2	Mm.307620
Fatty Acid	Lpin1	NM_015763	lipin 1	Mm.153625

Fatty Acid	Dgat2	NM_026384	diacylglycerol O-acyltransferase 2	Mm.180189
Fatty Acid	Abcd3	NM_008991	ATP-binding cassette, sub-family D (ALD), member 3	Mm.399042
Fatty Acid	Acnat2	NM_145368	acyl-coenzyme A amino acid N-acyltransferase 2	Mm.35071
Fatty Acid	Aacs	NM_030210	acetoacetyl-CoA synthetase	Mm.431573
Fatty Acid	Tnxb	NM_031176	tenascin XB	Mm.290527
Fatty Acid	Cygb	NM_030206	cytoglobin	Mm.34598
Fatty Acid	Prkag3	NM_153744	protein kinase, AMP-activated, gamma 3 non-catatlytic subunit	Mm.166501
Fatty Acid	Pparg	NM_001308352	peroxisome proliferator activated receptor gamma	Mm.3020
Fatty Acid	Pdk1	NM_172665	pyruvate dehydrogenase kinase, isoenzyme 1	Mm.34411
Fatty Acid	Adipor2	NM_197985	adiponectin receptor 2	Mm.291826
Fatty Acid	Ptgis	NM_008968	prostaglandin I2 (prostacyclin) synthase	Mm.2339
Fatty Acid	Pck1	NM_011044	phosphoenolpyruvate carboxykinase 1, cytosolic	Mm.266867
Fatty Acid	Acaa1b	NM_146230	acetyl-Coenzyme A acyltransferase 1B	Mm.379402
Fatty Acid	Slc27a2	NM_011978	solute carrier family 27 (fatty acid transporter), member 2	Mm.290044
Fatty Acid	Lpin3	NM_022883	lipin 3	Mm.292111
Fatty Acid	Decr2	NM_011933	2-4-dienoyl-Coenzyme A reductase 2, peroxisomal	Mm.292869
Fatty Acid	Hsd17b4	NM_008292	hydroxysteroid (17-beta) dehydrogenase 4	Mm.277857
Fatty Acid	Pnpla8	NM_026164	patatin-like phospholipase domain containing 8	Mm.54126
Fatty Acid	Decr1	NM_026172	2,4-dienoyl CoA reductase 1, mitochondrial	Mm.393293
Fatty Acid	Lpin2	NM_001164885	lipin 2	Mm.227924
Fatty Acid	Asah2	NM_018830	N-acylsphingosine amidohydrolase 2	Mm.491229
Fatty Acid	Acat2	NM_009338	acetyl-Coenzyme A acetyltransferase 2	Mm.439711
Fatty Acid	Prkaa2	NM_178143	protein kinase, AMP-activated, alpha 2 catalytic subunit	Mm.48638
Fatty Acid	Alox12	NM_001331118	arachidonate 12-lipoxygenase	Mm.12286
Fatty Acid	Acot11	NM_001347159	acyl-CoA thioesterase 11	Mm.222956

Fatty Acid	Adipoq	NM_009605	adiponectin, C1Q and collagen domain containing	Mm.3969
Fatty Acid	Cav1	NM_007616	caveolin 1, caveolae protein	Mm.28278
Fatty Acid	Sesn2	NM_144907	sestrin 2	Mm.23608
Fatty Acid	Acox3	NM_030721	acyl-Coenzyme A oxidase 3, pristanoyl	Mm.291503
Fatty Acid	Cyp2a5	NM_007812	cytochrome P450, family 2, subfamily a, polypeptide 5	Mm.389848
Fatty Acid	Slc45a3	NM_145977	solute carrier family 45, member 3	Mm.200307
Fatty Acid	Acat3	NM_153151	acetyl-Coenzyme A acetyltransferase 3	Mm.490312
Fatty Acid	Nr1h3	NM_013839	nuclear receptor subfamily 1, group H, member 3	Mm.22690
Fatty Acid	Elovl5	NM_134255	ELOVL family member 5, elongation of long chain fatty acids (yeast)	Mm.430736
Fatty Acid	Srebf1	NM_011480	sterol regulatory element binding transcription factor 1	Mm.278701
Fatty Acid	Lep	NM_008493	leptin	Mm.277072
Fatty Acid	Them4	NM_029431	thioesterase superfamily member 4	Mm.45092
Fatty Acid	Crat	NM_007760	carnitine acetyltransferase	Mm.20396
Fatty Acid	Cyp2d9	NM_010006	cytochrome P450, family 2, subfamily d, polypeptide 9	Mm.226708
Fatty Acid	Lypla1	NM_008866	lysophospholipase 1	Mm.299955
Fatty Acid	Ptgds	NM_008963	prostaglandin D2 synthase (brain)	Mm.1008
Fatty Acid	Acsl4	NM_001033600	acyl-CoA synthetase long-chain family member 4	Mm.391337
Fatty Acid	Ivd	NM_019826	isovaleryl coenzyme A dehydrogenase	Mm.6635
Fatty Acid	Acadl	NM_007381	acyl-Coenzyme A dehydrogenase, long-chain	Mm.2445
Fatty Acid	Pank2	NM_153501	pantothenate kinase 2	Mm.101264
Fatty Acid	Abcd2	NM_011994	ATP-binding cassette, sub-family D (ALD), member 2	Mm.295456
Fatty Acid	Acot1	NM_012006	acyl-CoA thioesterase 1	Mm.1978
Fatty Acid	Elovl7	NM_029001	ELOVL family member 7, elongation of long chain fatty acids (yeast)	Mm.286127
Fatty Acid	Faah	NM_010173	fatty acid amide hydrolase	Mm.256025
Fatty Acid	Acsl3	NM_001136222	acyl-CoA synthetase long-chain family member 3	Mm.276016

Fatty Acid	Ephx2	NM_001271403	epoxide hydrolase 2, cytoplasmic	Mm.15295
Fatty Acid	Acnat2	NM_145368	acyl-coenzyme A amino acid N-acyltransferase 2	Mm.35071
Fatty Acid	Ankrd23	NM_153502	ankyrin repeat domain 23	Mm.41421
Fatty Acid	Lep	NM_008493	leptin	Mm.277072
Fatty Acid	Acsm3	NM_212442	acyl-CoA synthetase medium-chain family member 3	Mm.334199
Fatty Acid	Adipor2	NM_197985	adiponectin receptor 2	Mm.291826
Fatty Acid	Avpr1a	NM_016847	arginine vasopressin receptor 1A	Mm.4351
Fatty Acid	Acat3	NM_153151	acetyl-Coenzyme A acetyltransferase 3	Mm.490312
Fatty Acid	Acox1	NM_015729	acyl-Coenzyme A oxidase 1, palmitoyl	Mm.356689
Fatty Acid	Srebf1	NM_011480	sterol regulatory element binding transcription factor 1	Mm.278701
Fatty Acid	Prkag3	NM_153744	protein kinase, AMP-activated, gamma 3 non-catatlytic subunit	Mm.166501
Fatty Acid	Cyp4a31	NM_001252539	cytochrome P450, family 4, subfamily a, polypeptide 31	Mm.482086
Fatty Acid	Scd2	NM_009128	stearoyl-Coenzyme A desaturase 2	Mm.487021
Glycolysis	Idh1	NM_010497	isocitrate dehydrogenase 1 (NADP+), soluble	Mm.9925
Glycolysis	Pgam1	NM_023418	phosphoglycerate mutase 1	Mm.16783
Glycolysis	Igfbp5	NM_010518	insulin-like growth factor binding protein 5	Mm.405761
Glycolysis	Tnf	NM_001278601	tumor necrosis factor	Mm.1293
Glycolysis	Atf4	NM_009716	activating transcription factor 4	Mm.641
Glycolysis	Sirt6	NM_001163430	sirtuin 6	Mm.25643
Glycolysis	Gale	NM_178389	galactose-4-epimerase, UDP	Mm.247946
Glycolysis	Nr1d1	NM_145434	nuclear receptor subfamily 1, group D, member 1	Mm.390397
Glycolysis	C1qtnf2	NM_026979	C1q and tumor necrosis factor related protein 2	Mm.390374
Glycolysis	Lcmt1	NM_025304	leucine carboxyl methyltransferase 1	Mm.260527
Glycolysis	Arpp19	NM_001142655	cAMP-regulated phosphoprotein 19	Mm.247837
Glycolysis	Oma1	NM_025909	OMA1 zinc metallopeptidase	Mm.30021
Glycolysis	Eif6	NM_010579	eukaryotic translation initiation factor 6	Mm.271674
Glycolysis	Galk1	NM_016905	galactokinase 1	Mm.2820

Glycolysis	Tcf7l2	NM_001331144	transcription factor 7 like 2, T cell specific, HMG box	Mm.139815
Glycolysis	Ldha	NM_010699	lactate dehydrogenase A	Mm.29324
Glycolysis	Dcxr	NM_001347608	dicarbonyl L-xylulose reductase	Mm.231091
Glycolysis	Src	NM_009271	Rous sarcoma oncogene	Mm.22845
Glycolysis	Trp53	NM_001127233	transformation related protein 53	Mm.222
Glycolysis	Igfbp3	NM_008343	insulin-like growth factor binding protein 3	Mm.29254
Glycolysis	C1qtnf1	NM_001204129	C1q and tumor necrosis factor related protein 1	Mm.23845
Glycolysis	Mapk14	NM_001168508	mitogen-activated protein kinase 14	Mm.311337
Glycolysis	Prkag2	NM_001170556	protein kinase, AMP-activated, gamma 2 non-catalytic subunit	Mm.33649
Glycolysis	Mlycd	NM_019966	malonyl-CoA decarboxylase	Mm.423037
Glycolysis	Slc35b4	NM_021435	solute carrier family 35, member B4	Mm.245527
Glycolysis	Ugp2	NM_139297	UDP-glucose pyrophosphorylase 2	Mm.28877
Glycolysis	H6pd	NM_173371	hexose-6-phosphate dehydrogenase (glucose 1-dehydrogenase)	Mm.22183
Glycolysis	Galt	NM_001302511	galactose-1-phosphate uridyl transferase	Mm.439669
Glycolysis	Pfkm	NM_001163487	phosphofructokinase, muscle	Mm.272582
Glycolysis	Rpia	NM_009075	ribose 5-phosphate isomerase A	Mm.17905
Glycolysis	Ganc	NM_172672	glucosidase, alpha; neutral C	Mm.38851
Glycolysis	Ppara	NM_001113418	peroxisome proliferator activated receptor alpha	Mm.212789
Glycolysis	Ppargc1a	NR_132764	peroxisome proliferative activated receptor, gamma, coactivator 1 alpha	Mm.259072
Glycolysis	Pdk2	NM_133667	pyruvate dehydrogenase kinase, isoenzyme 2	Mm.29768
Glycolysis	Inpp5k	NM_008916	inositol polyphosphate 5-phosphatase K	Mm.1458
Glycolysis	Nr1d1	NM_145434	nuclear receptor subfamily 1, group D, member 1	Mm.390397
Glycolysis	Fabp5	NM_001272097	fatty acid binding protein 5, epidermal	Mm.741
Glycolysis	Mst1	NM_008243	macrophage stimulating 1 (hepatocyte growth factor-like)	Mm.8369
Glycolysis	Per2	NM_011066	period circadian clock 2	Mm.482463

Glycolysis	Dgat2	NM_026384	diacylglycerol O-acyltransferase 2	Mm.180189
Glycolysis	Igfbp4	NM_010517	insulin-like growth factor binding protein 4	Mm.233799
Glycolysis	Prkag3	NM_153744	protein kinase, AMP-activated, gamma 3 non-catatlytic subunit	Mm.166501
Glycolysis	Esrrb	NM_011934	estrogen related receptor, beta	Mm.235550
Glycolysis	Pdk1	NM_172665	pyruvate dehydrogenase kinase, isoenzyme 1	Mm.34411
Glycolysis	Dlat	NM_145614	dihydrolipoamide S-acetyltransferase (E2 component of pyruvate dehydrogenase complex)	Mm.285076
Glycolysis	Entpd5	NM_001286049	ectonucleoside triphosphate diphosphohydrolase 5	Mm.10211
Glycolysis	Pck1	NM_011044	phosphoenolpyruvate carboxykinase 1, cytosolic	Mm.266867
Glycolysis	Zbtb7a	NM_010731	zinc finger and BTB domain containing 7a	Mm.20920
Glycolysis	Ier3	NM_133662	immediate early response 3	Mm.25613
Glycolysis	Gys1	NM_030678	glycogen synthase 1, muscle	Mm.275654
Glycolysis	Prkaa2	NM_178143	protein kinase, AMP-activated, alpha 2 catalytic subunit	Mm.48638
Glycolysis	Adipoq	NM_009605	adiponectin, C1Q and collagen domain containing	Mm.3969
Glycolysis	Idh3a	NM_029573	isocitrate dehydrogenase 3 (NAD+) alpha	Mm.279195
Glycolysis	Foxo1	NM_019739	forkhead box O1	Mm.29891
Glycolysis	Sesn2	NM_144907	sestrin 2	Mm.23608
Glycolysis	Lepr	NM_001122899	leptin receptor	Mm.259282
Glycolysis	Nr3c1	NM_008173	nuclear receptor subfamily 3, group C, member 1	Mm.129481
Glycolysis	Rora	NM_013646	RAR-related orphan receptor alpha	Mm.378450
Glycolysis	Rorc	NM_011281	RAR-related orphan receptor gamma	Mm.4372
Glycolysis	Slc45a3	NM_145977	solute carrier family 45, member 3	Mm.200307
Glycolysis	G6pc	NM_008061	glucose-6-phosphatase, catalytic	Mm.18064
Glycolysis	Ncor1	NM_011308	nuclear receptor co-repressor 1	Mm.271814
Glycolysis	Lep	NM_008493	leptin	Mm.277072
Glycolysis	Kat2a	NM_020004	K(lysine) acetyltransferase 2A	Mm.218837
Glycolysis	G6pdx	NM_008062	glucose-6-phosphate dehydrogenase X-linked	Mm.27210

Glycolysis	Cbr2	NM_007621	carbonyl reductase 2	Mm.21454
Glycolysis	Igfbp4	NM_010517	insulin-like growth factor binding protein 4	Mm.233799
Glycolysis	Npy1r	NM_010934	neuropeptide Y receptor Y1	Mm.5112
Glycolysis	Pfkp	NM_001291071	phosphofructokinase, platelet	Mm.273874
Glycolysis	Gale	NM_178389	galactose-4-epimerase, UDP	Mm.247946
Glycolysis	Prps2	NM_026662	phosphoribosyl pyrophosphate synthetase 2	Mm.272955
Glycolysis	Pgm5	NM_175013	phosphoglucomutase 5	Mm.105222
Glycolysis	Rorc	NM_011281	RAR-related orphan receptor gamma	Mm.4372
Glycolysis	Lep	NM_008493	leptin	Mm.277072
Glycolysis	Rbp4	NM_001159487	retinol binding protein 4, plasma	Mm.2605
Glycolysis	Prkag3	NM_153744	protein kinase, AMP-activated, gamma 3 non-catatlytic subunit	Mm.166501
Ox phphos/mit	Bid	NM_007544	BH3 interacting domain death agonist	Mm.235081
Ox phphos/mit	Cox6a1	NM_007748	cytochrome c oxidase subunit VIa polypeptide 1	Mm.43415
Ox phphos/mit	Msh2	NM_008628	mutS homolog 2	Mm.4619
Ox phphos/mit	Pde2a	NM_001243758	phosphodiesterase 2A, cGMP-stimulated	Mm.247564
Ox phphos/mit	Slc25a33	NM_027460	solute carrier family 25, member 33	Mm.41877
Ox phphos/mit	Ndufs4	NM_010887	NADH dehydrogenase (ubiquinone) Fe-S protein 4	Mm.253142
Ox phphos/mit	Mecp2	NM_010788	methyl CpG binding protein 2	Mm.131408
Ox phphos/mit	Foxred1	NM_001291448	FAD-dependent oxidoreductase domain containing 1	Mm.138512
Ox phphos/mit	Foxred1	NM_001291448	FAD-dependent oxidoreductase domain containing 1	Mm.138512
Transporter	Slc7a8	NM_016972	solute carrier family 7 (cationic amino acid transporter, y+ system), member 8	Mm.276831
Transporter	Slc22a12	NM_009203	solute carrier family 22 (organic anion/cation transporter), member 12	Mm.391146
Transporter	Slc22a12	NM_009203	solute carrier family 22 (organic anion/cation transporter), member 12	Mm.391146

Transporter	Slc25a15	NM_181325	solute carrier family 25 (mitochondrial carrier ornithine transporter), member 15	Mm.200907
Transporter	Slc3a2	NM_001161413	solute carrier family 3 (activators of dibasic and neutral amino acid transport), member 2	Mm.4114
Transporter	Aqp8	NM_001109045	aquaporin 8	Mm.273175
Transporter	Slc24a3	NM_053195	solute carrier family 24 (sodium/potassium/calcium exchanger), member 3	Mm.217171
Transporter	Slc16a10	NM_028247	solute carrier family 16 (monocarboxylic acid transporters), member 10	Mm.186778
Transporter	Slc5a2	NM_133254	solute carrier family 5 (sodium/glucose cotransporter), member 2	Mm.38870
Transporter	Slc31a2	NM_025286	solute carrier family 31, member 2	Mm.292539
Transporter	Slc16a1	NM_009196	solute carrier family 16 (monocarboxylic acid transporters), member 1	Mm.9086
Transporter	Slc4a1ap	NM_009206	solute carrier family 4 (anion exchanger), member 1, adaptor protein	Mm.352407
Transporter	Slc35f1	NM_178675	solute carrier family 35, member F1	Mm.338690
Transporter	Slc35a3	NM_144902	solute carrier family 35 (UDP-N-acetylglucosamine (UDP-GlcNAc) transporter), member 3	Mm.190758
Transporter	Slc22a17	NM_021551	solute carrier family 22 (organic cation transporter), member 17	Mm.27435
Transporter	Slc25a19	NM_001252395	solute carrier family 25 (mitochondrial thiamine pyrophosphate carrier), member 19	Mm.383426
Transporter	Slc29a3	NM_023596	solute carrier family 29 (nucleoside transporters), member 3	Mm.284462
Transporter	Slc34a3	NM_080854	solute carrier family 34 (sodium phosphate), member 3	Mm.346652
Transporter	Slc35c2	NM_001252575	solute carrier family 35, member C2	Mm.21184
Transporter	Slc15a3	NM_023044	solute carrier family 15, member 3	Mm.27387

Transporter	Slc7a7	NM_011405	solute carrier family 7 (cationic amino acid transporter, y+ system), member 7	Mm.142455
Transporter	Slc41a3	NM_027868	solute carrier family 41, member 3	Mm.272633
Transporter	Slc2a6	NM_172659	solute carrier family 2 (facilitated glucose transporter), member 6	Mm.41203
Transporter	Slc5a11	NM_146198	solute carrier family 5 (sodium/glucose cotransporter), member 11	Mm.211838
Transporter	Slc31a1	NM_175090	solute carrier family 31, member 1	Mm.248637
Transporter	Slc35b3	NM_134060	solute carrier family 35, member B3	Mm.255506
Transporter	Slc22a18	NM_001042760	solute carrier family 22 (organic cation transporter), member 18	Mm.271740
Transporter	Slc26a6	NM_134420	solute carrier family 26, member 6	Mm.45201
Transporter	Slc26a6	NM_134420	solute carrier family 26, member 6	Mm.45201
Transporter	Slc39a8	NM_001135150	solute carrier family 39 (metal ion transporter), member 8	Mm.30239
Transporter	Slc22a12	NM_009203	solute carrier family 22 (organic anion/cation transporter), member 12	Mm.391146
Transporter	Slc22a12	NM_009203	solute carrier family 22 (organic anion/cation transporter), member 12	Mm.391146
Transporter	Slc35f3	NM_175434	solute carrier family 35, member F3	Mm.405238
Transporter	Slc22a4	NM_001330304	solute carrier family 22 (organic cation transporter), member 4	Mm.274590
Transporter	Slc25a33	NM_027460	solute carrier family 25, member 33	Mm.41877
Transporter	Slc12a7	NM_011390	solute carrier family 12, member 7	Mm.275800
Transporter	Slc13a2	NM_022411	solute carrier family 13 (sodium-dependent dicarboxylate transporter), member 2	Mm.274058
Transporter	Slc25a42	NM_001007570	solute carrier family 25, member 42	Mm.185413
Transporter	Slc6a19	NM_028878	solute carrier family 6 (neurotransmitter transporter), member 19	Mm.271635
Transporter	Slc9a3r1	NM_012030	solute carrier family 9 (sodium/hydrogen exchanger), member 3 regulator 1	Mm.27842

Transporter	Slc6a9	NM_008135	solute carrier family 6 (neurotransmitter transporter, glycine), member 9	Mm.244549
Transporter	Slc46a1	NM_026740	solute carrier family 46, member 1	Mm.131618
Transporter	Slc26a1	NM_001310691	solute carrier family 26 (sulfate transporter), member 1	Mm.440571
Transporter	Slc25a10	NM_013770	solute carrier family 25 (mitochondrial carrier, dicarboxylate transporter), member 10	Mm.3991
Transporter	Slc26a6	NM_134420	solute carrier family 26, member 6	Mm.45201
Transporter	Slc26a6	NM_134420	solute carrier family 26, member 6	Mm.45201
Transporter	Aqp11	NM_175105	aquaporin 11	Mm.29756
Transporter	Slc6a4	NM_010484	solute carrier family 6 (neurotransmitter transporter, serotonin), member 4	Mm.300318
Transporter	Slc25a37	NM_026331	solute carrier family 25, member 37	Mm.293635
Transporter	Slc5a9	NM_145551	solute carrier family 5 (sodium/glucose cotransporter), member 9	Mm.26630
Transporter	Slc35b4	NM_021435	solute carrier family 35, member B4	Mm.245527
Transporter	Slc25a26	NM_026255	solute carrier family 25 (mitochondrial carrier, phosphate carrier), member 26	Mm.280725
Transporter	Aqp3	NM_016689	aquaporin 3	Mm.34043
Transporter	Slc5a1	NM_019810	solute carrier family 5 (sodium/glucose cotransporter), member 1	Mm.25237
Transporter	Slc30a9	NM_178651	solute carrier family 30 (zinc transporter), member 9	Mm.234455
Transporter	Slc17a5	NM_172773	solute carrier family 17 (anion/sugar transporter), member 5	Mm.46932
Transporter	Slc7a9	NM_021291	solute carrier family 7 (cationic amino acid transporter, y+ system), member 9	Mm.45874
Transporter	Slc25a44	NM_001145876	solute carrier family 25, member 44	Mm.301740

Transporter	Slc28a1	NM_001004184	solute carrier family 28 (sodium-coupled nucleoside transporter), member 1	Mm.389909
Transporter	Slc35e4	NM_153142	solute carrier family 35, member E4	Mm.171514
Transporter	Slc6a20b	NM_011731	solute carrier family 6 (neurotransmitter transporter), member 20B	Mm.41963
Transporter	Slc25a11	NM_024211	solute carrier family 25 (mitochondrial carrier oxoglutarate carrier), member 11	Mm.296082
Transporter	Slc38a10	NM_001164802	solute carrier family 38, member 10	Mm.253403
Transporter	Slc27a5	NM_009512	solute carrier family 27 (fatty acid transporter), member 5	Mm.10984
Transporter	Slc25a46	NM_026165	solute carrier family 25, member 46	Mm.23896
Transporter	Slc5a6	NM_177870	solute carrier family 5 (sodium-dependent vitamin transporter), member 6	Mm.205463
Transporter	Slc7a4	NM_144852	solute carrier family 7 (cationic amino acid transporter, y+ system), member 4	Mm.298878
Transporter	Slc17a4	NM_177016	solute carrier family 17 (sodium phosphate), member 4	Mm.97959
Transporter	Slc12a6	NM_133648	solute carrier family 12, member 6	Mm.491155
Transporter	Slc46a3	NM_027872	solute carrier family 46, member 3	Mm.153218
Transporter	Slc16a9	NM_025807	solute carrier family 16 (monocarboxylic acid transporters), member 9	Mm.19325
Transporter	Slc25a20	NM_020520	solute carrier family 25 (mitochondrial carnitine/acylcarnitine translocase), member 20	Mm.29666
Transporter	Slc22a5	NM_011396	solute carrier family 22 (organic cation transporter), member 5	Mm.42253
Transporter	Slc25a34	NM_001013780	solute carrier family 25, member 34	Mm.295682
Transporter	Slc27a2	NM_011978	solute carrier family 27 (fatty acid transporter), member 2	Mm.290044
Transporter	Slc17a2	NM_144836	solute carrier family 17 (sodium phosphate), member 2	Mm.24030

Transporter	Slc5a8	NM_145423	solute carrier family 5 (iodide transporter), member 8	Mm.77381
Transporter	Slc1a3	NM_148938	solute carrier family 1 (glial high affinity glutamate transporter), member 3	Mm.204834
Transporter	Slc37a4	NM_001293631	solute carrier family 37 (glucose-6-phosphate transporter), member 4	Mm.30087
Transporter	Slc2a13	NM_001033633	solute carrier family 2 (facilitated glucose transporter), member 13	Mm.360596
Transporter	Slc35e3	NM_029875	solute carrier family 35, member E3	Mm.256753
Transporter	Slco2a1	NM_033314	solute carrier organic anion transporter family, member 2a1	Mm.207106
Transporter	Slc7a2	NM_007514	solute carrier family 7 (cationic amino acid transporter, y+ system), member 2	Mm.4676
Transporter	Slc25a13	NM_001177572	solute carrier family 25 (mitochondrial carrier, adenine nucleotide translocator), member 13	Mm.24513
Transporter	Slc16a14	NM_027921	solute carrier family 16 (monocarboxylic acid transporters), member 14	Mm.158754
Transporter	Slc5a3	NM_017391	solute carrier family 5 (inositol transporters), member 3	Mm.217354
Transporter	Slc25a25	NM_001164358	solute carrier family 25 (mitochondrial carrier, phosphate carrier), member 25	Mm.37395
Transporter	Slc15a4	NM_133895	solute carrier family 15, member 4	Mm.28506
Transporter	Slc44a1	NM_133891	solute carrier family 44, member 1	Mm.482207
Transporter	Slco3a1	NM_023908	solute carrier organic anion transporter family, member 3a1	Mm.425467
Transporter	Slc2a1	NM_011400	solute carrier family 2 (facilitated glucose transporter), member 1	Mm.21002
Transporter	Slc45a3	NM_145977	solute carrier family 45, member 3	Mm.200307
Transporter	Slc6a6	NM_009320	solute carrier family 6 (neurotransmitter transporter, taurine), member 6	Mm.395650
Transporter	Slc25a35	NM_028048	solute carrier family 25, member 35	Mm.298622
Transporter	Slc17a1	NR_121611	solute carrier family 17 (sodium phosphate), member 1	Mm.2656

Transporter	Slc22a13	NM_133980	solute carrier family 22 (organic cation transporter), member 13	Mm.38775
Transporter	Slc6a15	NM_175328	solute carrier family 6 (neurotransmitter transporter), member 15	Mm.458408
Transporter	Slc22a7	NM_144856	solute carrier family 22 (organic anion transporter), member 7	Mm.387538
Transporter	Slc9a3r2	NM_023449	solute carrier family 9 (sodium/hydrogen exchanger), member 3 regulator 2	Mm.21587
Transporter	Slc19a3	NM_030556	solute carrier family 19, member 3	Mm.261542
Transporter	Slc35f2	NM_028060	solute carrier family 35, member F2	Mm.26159
Transporter	Slc35b1	NM_016752	solute carrier family 35, member B1	Mm.4593
Transporter	Slc15a2	NM_021301	solute carrier family 15 (H+/peptide transporter), member 2	Mm.281804
Transporter	Slc10a3	NM_001256104	solute carrier family 10 (sodium/bile acid cotransporter family), member 3	Mm.19931
Transporter	Slc6a12	NM_133661	solute carrier family 6 (neurotransmitter transporter, betaine/GABA), member 12	Mm.274506
Transporter	Slc11a2	NM_008732	solute carrier family 11 (proton-coupled divalent metal ion transporters), member 2	Mm.234608
Transporter	Slc25a38	NM_144793	solute carrier family 25, member 38	Mm.236656

Supplementary Table 3: List of transporters belonging to the Slc family				
**Name**	**Transport**	**Slc family**	**Young ANOVA Padj**	**Nephritic ANOVA Padj**
Slc15a2	small peptides	Proton oligopeptide cotransporter	**0.017**	0.99
Slc15a3	peptides/histidine	Proton oligopeptide cotransporter	**0.008**	0.46
Slc16a10	aromatic amino acids	Monocarboxylate transporter	**0.000**	0.39
Slc1a3	neutral amino acids	High-affinity glutamate and neutral amino acid transporter	**0.001**	0.41
Slc3a2	neutral amino acids - colocalizes with Slc7a8	Heavy subunits of the heteromeric amino acid transporters	**0.000**	0.57
Slc6a15	neutral amino acids	Sodium- and chloride-dependent neurotransmitter transporter	**0.002**	0.24
Slc6a19	neutral amino acids	Sodium- and chloride-dependent neurotransmitter transporter	**0.000**	0.11
Slc6a20b	proline, sarcosine	Sodium- and chloride-dependent neurotransmitter transporter	**0.027**	0.61
Slc6a6	taurine	Sodium- and chloride-dependent neurotransmitter transporter	**0.002**	0.11
Slc6a9	glycine	Sodium- and chloride-dependent neurotransmitter transporter	**0.003**	0.45
Slc7a2	cationic amino acids	Cationic amino acid transporter/glycoprotein-associated	**0.037**	0.65
Slc7a4	cationic amino acids	Cationic amino acid transporter/glycoprotein-associated	**0.001**	**0.01**
Slc7a7	cationic amino acids	Cationic amino acid transporter/glycoprotein-associated	**0.002**	0.56
Slc7a8	neutral amino acids	Cationic amino acid transporter/glycoprotein-associated	**0.000**	0.10
Slc7a9	cationic amino acids	Cationic amino acid transporter/glycoprotein-associated	**0.033**	0.41
Slc6a4	serotonin	Sodium- and chloride-dependent neurotransmitter transporter	**0.001**	0.22
Slc6a12	GABA, betaine	Sodium- and chloride-dependent neurotransmitter transporter	**0.008**	0.18
Slc16a1	GABA	Monocarboxylate transporter	**0.006**	0.13
Slc12a6	Cl	Electroneutral cation-coupled Cl cotransporter	**0.000**	0.41
Slc12a7	Cl	Electroneutral cation-coupled Cl cotransporter	**0.000**	**0.05**
Slc17a2	PO4	Vesicular glutamate transporter	**0.001**	0.43
Slc17a4	Na/PO4	Vesicular glutamate transporter	**0.005**	**0.02**
Slc24a3	Na/K/Ca	Na+/(Ca2+-K+) exchanger	**0.002**	0.09
Slc34a3	Na/PO4	Type II Na+-phosphate cotransporter	**0.000**	0.19

Slc4a1ap	anions	Bicarbonate transporter	**0.023**	0.10
Slc9a3r1	Na/H	Na+/H+ exchanger	**0.000**	0.38
Slc9a3r2	Na/H	Na+/H+ exchanger	**0.006**	0.04
Slc2a1	glucose, galactose, mannose, glucosamine Glut1	Facilitative GLUT transporter	**0.000**	0.18
Slc2a6	glucose, galactose, fructose, mannose, glucosamine Glut 6	Facilitative GLUT transporter	**0.022**	0.29
Slc2a13	H/myoinositol	Facilitative GLUT transporter	**0.000**	0.08
Slc45a3	sucrose, glucose, fructose	H+/sugar cotransporter	**0.000**	0.14
Slc5a1	glucose galactose SGLT1	Sodium glucose cotransporter	**0.017**	0.08
Slc5a2	glucose SGLT2	Sodium glucose cotransporter	**0.024**	0.84
Slc5a3	glucose myoinositol	Sodium glucose cotransporter	**0.017**	0.72
Slc5a9	mannose, fructose, glucose	Sodium glucose cotransporter	**0.006**	0.36
Slc5a11	myoinositol	Sodium glucose cotransporter	**0.040**	0.24
Slc17a5	sialic acid, other acidic sugars	Vesicular glutamate transporter	**0.035**	0.08
Slc22a4	Na/carnitine low affinity	Organic cation/anion/zwitterion	**0.000**	0.18
Slc22a5	Na/carnitine high affinity	Organic cation/anion/zwitterion	**0.000**	0.94
Slc27a2	fatty acid	Fatty acid transporter	**0.003**	0.41
Slc5a8	short chain fatty acids	Sodium glucose cotransporter	**0.000**	0.61
Slc19a3	thiamine	Folate/thiamine transporter	**0.000**	0.47
Slc46a1	folate, heme	Folate transporter	**0.001**	0.23
Slc5a6	pantothenate, biotin, lipoate	Sodium glucose cotransporter	**0.000**	**0.02**
Slc30a9	Zn Cd Mg	Zinc efflux	**0.007**	0.06
Slc31a1	Cu	Copper transporter	**0.019**	0.46
Slc31a2	Cu	Copper transporter	**0.001**	0.68
Slc41a3	Mg	MgtE-like magnesium transporter	**0.013**	0.36
Slc28a1	nucleoside	Na+-coupled nucleoside transport	**0.038**	0.51
Slc29a3	nucleoside	Facilitative nucleoside transporter	**0.000**	0.32
Slc35b1	nucleoside	Nucleoside sugar	**0.014**	**0.05**
Slc35b3	nucleoside	Nucleoside sugar	**0.013**	0.53
Slc35b4	nucleoside	Nucleoside sugar	**0.010**	0.38

Slc35c2	nucleoside	Nucleoside sugar	**0.000**	0.11
Slc35e3	nucleoside	Nucleoside sugar	**0.004**	0.14
Slc35e4	nucleoside	Nucleoside sugar	**0.044**	0.18
Slc35f1	nucleoside	Nucleoside sugar	**0.047**	0.28
Slc35f2	nucleoside	Nucleoside sugar	**0.001**	0.51
Slc35f3	nucleoside	Nucleoside sugar	**0.037**	**0.01**
Slc13a2	dicarboxylates	Human Na+-sulfate/carboxylate cotransporter	**0.000**	0.16
Slc16a14	monocarboxylates	Monocarboxylate transporter	**0.001**	0.16
Slc16a9	monocarboxylates	Monocarboxylate transporter	**0.000**	0.63
Slc26a1	monocarboxylates	Multifunctional anion exchanger	**0.000**	0.40
Slc26a6	monocarboxylates	Multifunctional anion exchanger	**0.000**	**0.01**
Slc25a10	dicarboxylates	Mitochondrial carrier	**0.003**	0.10
Slc25a13	aspartate, glutamate	Mitochondrial carrier	**0.019**	0.11
Slc25a15	ornithine	Mitochondrial carrier	**0.000**	0.66
Slc25a19	nucleosides	Mitochondrial carrier	**0.004**	**0.05**
Slc25a20	acylcarnitines	Mitochondrial carrier	**0.000**	0.07
Slc25a25	nucleosides	Mitochondrial carrier	**0.044**	0.35
Slc25a26	S-adenosyl-methionine, S-adenosyl-homocysteine	Mitochondrial carrier	**0.031**	0.52
Slc25a33	pyrimidines	Mitochondrial carrier	**0.000**	0.40
Slc25a34	unknown	Mitochondrial carrier	**0.000**	0.37
Slc25a35	unknown	Mitochondrial carrier	**0.000**	0.10
Slc25a37	Fe	Mitochondrial carrier	**0.002**	0.37
Slc25a42	nucleosides	Mitochondrial carrier	**0.000**	0.44
Slc25a44	unknown	Mitochondrial carrier	**0.025**	0.69
Slc17a1	organic anions/PO4/Cl	Vesicular glutamate transporter	**0.026**	0.91
Slc22a12	urate URAT1	Organic cation/anion/zwitterion	**0.000**	**0.05**
Slc22a13	urate organic anions OAT10	Organic cation/anion/zwitterion	**0.046**	0.68
Slc22a18	organic anions	Organic cation/anion/zwitterion	**0.036**	0.91
Slc22a7	organic anions OAT2	Organic cation/anion/zwitterion	**0.030**	0.20
Slc10a3	unknown	Sodium bile salt cotransport	**0.034**	0.23
Slc37a4	unknown	Sugar-phosphate/phosphate exchanger	**0.033**	0.44
Slc46a3	?lysosomal	Folate transporter	**0.000**	0.16
Slc15a4	peptides/histidine	Proton oligopeptide cotransporter	0.146	**0.05**
Slc27a5	bile acids	Fatty acid transporter	0.585	**0.05**
Slc44a1	choline	Choline-like transporter	0.263	**0.05**
Slc11a2	Fe	Proton-coupled metal ion transporter	0.405	**0.04**

Slc39a8	Zn Cd Mg	Metal ion transporter	0.149	**0.03**
Slc35a3	nucleoside	Nucleoside sugar	0.292	**0.04**
Slc25a11	oxoglutarate, malate	Mitochondrial carrier	0.283	**0.01**
Slc25a38	?glycine	Mitochondrial carrier	0.051	**0.04**
Slc25a46	unknown	Mitochondrial carrier	0.582	**0.02**
Slc22a17	unknown	Organic cation/anion/zwitterion	0.148	**0.05**
Slc38a10	unknown	System A and System N sodium-coupled neutral amino acid transporter	0.632	**0.01**

Supplementary Table 4: List of 320 diurnally regulated genes corrected by ≥50% and not corrected by remission induction

	**A. Mean Normalized Expression (RNASeq)**								
**Corrected**	**Remission**			**Young**			**Nephritic**		
**Symbol**	**ZT0**	**ZT12**	***Ratio ZT0:ZT12***	**ZT0**	**ZT12**	***Ratio ZT0:ZT12***	**ZT0**	**ZT12**	***Ratio ZT0:ZT12***
**4930550C14Rik**	1.13	0.73	*1.55*	1.30	0.76	*1.72*	1.14	0.89	*1.28*
**Acaa1b**	0.86	1.43	*0.60*	0.77	1.61	*0.48*	0.91	0.90	*1.02*
**Acat3**	0.82	1.29	*0.63*	0.90	1.14	*0.79*	1.22	1.02	*1.20*
**Acot3**	0.88	1.23	*0.71*	1.00	1.96	*0.51*	0.69	0.61	*1.13*
**Acox3**	0.85	1.32	*0.64*	0.86	1.29	*0.67*	0.89	0.89	*1.00*
**Aifm2**	0.95	1.25	*0.76*	0.93	1.36	*0.69*	0.97	0.87	*1.12*
**Ak5**	1.40	0.91	*1.55*	1.22	0.85	*1.44*	1.05	0.92	*1.14*
**Akr1c19**	0.83	1.19	*0.70*	0.60	1.01	*0.59*	1.09	1.14	*0.96*
**Alas1**	1.17	1.66	*0.71*	0.97	1.95	*0.50*	1.09	1.16	*0.94*
**Amt**	1.32	0.90	*1.48*	1.35	0.95	*1.42*	0.99	0.91	*1.09*
**Angptl4**	0.32	2.47	*0.13*	0.35	1.72	*0.20*	1.18	1.81	*0.65*
**Ankrd12**	0.83	1.10	*0.75*	0.81	1.18	*0.69*	1.09	1.11	*0.98*
**Aqp11**	0.86	1.07	*0.80*	0.98	1.24	*0.79*	0.70	0.70	*1.00*
**Aqp8**	1.36	0.29	*4.73*	2.21	0.71	*3.10*	0.96	0.47	*2.04*
**Arhgef3**	0.93	1.27	*0.73*	0.79	1.17	*0.68*	1.08	1.11	*0.98*
**Arntl**	1.88	0.14	*13.73*	3.92	0.33	*11.73*	1.04	0.76	*1.38*
**Avpi1**	0.94	1.15	*0.82*	0.88	1.15	*0.77*	0.97	0.85	*1.15*
**Azin1**	0.96	1.11	*0.87*	0.91	1.19	*0.76*	0.96	0.98	*0.97*
**Blvrb**	0.88	1.18	*0.75*	0.83	1.21	*0.69*	0.94	0.91	*1.03*
**Car5b**	1.16	0.81	*1.43*	1.64	0.98	*1.67*	1.13	0.98	*1.15*
**Cdc42ep4**	0.86	1.21	*0.71*	0.82	1.12	*0.74*	1.13	1.09	*1.04*
**Cdkl1**	1.15	0.60	*1.91*	1.43	0.86	*1.67*	0.94	0.80	*1.17*
**Cdo1**	0.78	1.26	*0.62*	0.80	1.09	*0.73*	1.01	0.96	*1.05*
**Chst1**	0.75	1.44	*0.52*	0.69	1.27	*0.55*	0.96	1.11	*0.86*
**Chst8**	0.86	1.06	*0.80*	0.74	1.15	*0.64*	0.90	0.87	*1.03*
**Cited4**	0.89	1.73	*0.52*	0.97	1.57	*0.62*	1.13	1.06	*1.06*
**Ckb**	1.34	0.78	*1.73*	1.09	0.73	*1.49*	1.01	0.87	*1.16*

**Clcn2**	0.87	1.18	*0.74*	0.85	1.44	*0.59*	0.93	0.93	*1.00*
**Cldn10**	1.41	0.84	*1.68*	1.41	0.91	*1.55*	0.75	0.79	*0.94*
**Clec2d**	0.67	1.40	*0.48*	0.83	1.25	*0.66*	1.10	1.03	*1.07*
**Clock**	1.29	0.79	*1.62*	1.27	0.81	*1.57*	1.08	1.14	*0.94*
**Clpx**	1.31	0.91	*1.44*	1.78	0.93	*1.91*	0.86	0.90	*0.96*
**Clstn3**	0.66	1.40	*0.48*	0.83	1.45	*0.57*	1.00	0.81	*1.24*
**Cmc1**	0.80	1.09	*0.73*	0.89	1.15	*0.78*	0.98	0.95	*1.03*
**Col27a1**	0.51	1.00	*0.51*	0.94	1.70	*0.55*	0.92	1.13	*0.82*
**Col4a4**	1.22	0.79	*1.55*	1.23	0.89	*1.38*	0.96	0.87	*1.10*
**Coq10b**	0.82	1.57	*0.52*	0.85	2.11	*0.40*	0.86	1.16	*0.75*
**Coro2a**	0.72	1.26	*0.57*	0.82	1.12	*0.73*	1.05	1.09	*0.96*
**Cpeb3**	0.78	1.30	*0.60*	0.78	1.23	*0.63*	0.82	1.00	*0.81*
**Cpt2**	0.92	1.31	*0.70*	0.76	1.21	*0.63*	0.87	1.01	*0.86*
**Creb3l1**	1.19	0.63	*1.88*	1.32	0.80	*1.65*	1.08	0.87	*1.23*
**Cry2**	0.63	1.34	*0.47*	0.66	1.44	*0.45*	0.77	0.95	*0.81*
**Csnk1g3**	0.92	1.21	*0.76*	0.84	1.25	*0.67*	0.86	1.00	*0.86*
**Ctgf**	0.57	1.56	*0.36*	0.59	1.41	*0.42*	1.23	1.79	*0.69*
**Cygb**	0.75	1.50	*0.50*	0.54	0.87	*0.62*	1.23	1.54	*0.80*
**Cyp27a1**	0.76	1.24	*0.61*	0.86	1.29	*0.67*	0.93	0.97	*0.95*
**Cyp2a5**	0.47	2.02	*0.23*	0.73	2.30	*0.32*	0.53	0.75	*0.70*
**Cyp2e1**	0.80	2.48	*0.32*	0.70	2.10	*0.33*	0.93	0.86	*1.08*
**D930015E06Rik**	0.76	1.13	*0.67*	0.70	1.02	*0.68*	1.03	1.12	*0.92*
**Dbp**	0.57	12.60	*0.04*	0.25	5.98	*0.04*	0.58	1.03	*0.57*
**Dcun1d3**	0.99	1.22	*0.81*	0.97	1.30	*0.75*	1.03	1.02	*1.01*
**Ddit4l**	1.31	0.57	*2.31*	1.41	0.65	*2.18*	1.22	1.07	*1.14*
**Dgat2**	0.77	1.41	*0.55*	0.95	1.52	*0.62*	0.77	0.85	*0.91*
**Dhrs7**	0.88	1.24	*0.71*	0.92	1.24	*0.74*	0.94	1.00	*0.94*
**Dleu7**	1.55	0.68	*2.28*	1.71	0.87	*1.97*	0.91	0.87	*1.04*
**Dnase1**	0.75	1.24	*0.60*	0.99	1.48	*0.67*	0.52	0.36	*1.46*
**Dusp1**	0.86	1.84	*0.47*	1.02	2.56	*0.40*	0.95	1.03	*0.92*
**Dusp14**	1.17	0.78	*1.50*	1.44	0.96	*1.50*	1.03	0.92	*1.12*
**Dusp7**	0.98	1.55	*0.63*	0.82	1.56	*0.53*	1.23	1.10	*1.12*
**Eef2k**	0.86	1.06	*0.81*	0.81	1.11	*0.74*	0.97	1.00	*0.97*
**Efhd1**	1.38	0.77	*1.80*	1.49	0.83	*1.78*	1.09	0.85	*1.29*
**Eif4ebp3**	0.74	1.26	*0.58*	0.92	1.23	*0.75*	0.97	0.90	*1.07*
**Ephx1**	0.87	1.27	*0.69*	0.71	1.35	*0.52*	0.90	1.08	*0.84*
**Errfi1**	0.79	1.35	*0.58*	1.11	1.77	*0.63*	0.97	1.07	*0.91*
**Fabp3**	0.99	1.14	*0.87*	0.90	1.14	*0.79*	0.85	0.72	*1.17*
**Fam102a**	0.90	1.22	*0.74*	0.90	1.14	*0.79*	1.14	1.15	*1.00*
**Fam163a**	1.86	0.58	*3.21*	1.64	0.79	*2.07*	1.00	0.69	*1.45*
**Fermt1**	1.29	0.73	*1.77*	1.18	0.80	*1.48*	1.13	1.05	*1.08*

**Ffar3**	0.98	0.46	*2.12*	1.91	1.00	*1.92*	1.01	0.86	*1.18*
**Fkbp5**	0.56	2.74	*0.20*	0.59	2.28	*0.26*	0.94	1.78	*0.53*
**Fmnl1**	0.72	1.49	*0.49*	0.79	1.49	*0.53*	0.94	0.91	*1.04*
**Foxo1**	0.91	1.41	*0.64*	1.05	1.39	*0.75*	1.03	1.05	*0.98*
**Gja1**	0.72	1.32	*0.54*	1.02	1.61	*0.63*	0.88	0.81	*1.09*
**Gldc**	0.92	2.44	*0.38*	1.19	2.46	*0.49*	0.89	1.05	*0.85*
**Glul**	0.89	1.60	*0.56*	0.86	1.73	*0.50*	0.96	1.02	*0.94*
**Gnpda1**	1.08	0.84	*1.29*	1.55	1.01	*1.54*	0.96	1.00	*0.96*
**Grasp**	0.77	1.28	*0.60*	0.82	1.29	*0.64*	1.05	1.14	*0.92*
**Gstt2**	0.82	1.23	*0.67*	0.74	1.29	*0.58*	0.90	0.95	*0.94*
**Gypc**	0.94	1.27	*0.74*	0.98	1.71	*0.57*	0.74	0.79	*0.93*
**Herpud1**	0.68	1.94	*0.35*	0.78	2.16	*0.36*	0.95	1.31	*0.73*
**Hn1l**	1.19	0.65	*1.83*	1.31	0.62	*2.12*	1.22	0.98	*1.25*
**Hspb6**	0.84	1.17	*0.72*	0.77	1.11	*0.70*	0.93	0.94	*0.99*
**Idh1**	1.07	0.74	*1.45*	1.46	0.87	*1.68*	1.04	0.97	*1.06*
**Ier3**	0.75	1.28	*0.59*	0.78	1.69	*0.46*	1.14	1.45	*0.78*
**Ifrd2**	0.91	1.34	*0.68*	0.93	1.21	*0.77*	0.92	0.91	*1.01*
**Igfbp4**	0.73	1.39	*0.52*	0.77	1.30	*0.59*	0.90	1.05	*0.86*
**Igfbp5**	1.59	0.60	*2.67*	1.23	0.88	*1.39*	0.72	0.70	*1.02*
**Il17f**	2.07	0.68	*3.02*	2.21	1.19	*1.86*	0.57	0.84	*0.68*
**Il17rb**	0.88	1.08	*0.81*	0.78	1.14	*0.69*	1.16	1.04	*1.11*
**Immp2l**	0.80	1.33	*0.60*	0.94	1.32	*0.71*	0.91	0.84	*1.09*
**Insc**	2.01	0.40	*5.03*	2.71	0.61	*4.46*	1.37	0.64	*2.15*
**Itga6**	1.14	0.65	*1.75*	1.38	0.72	*1.91*	0.99	1.09	*0.90*
**Itgb6**	1.50	0.68	*2.21*	1.50	0.72	*2.09*	0.94	0.84	*1.12*
**Itprip**	0.84	1.44	*0.58*	0.78	1.26	*0.62*	1.11	1.18	*0.94*
**Kcnj15**	0.92	1.08	*0.85*	0.93	1.25	*0.74*	0.93	0.97	*0.96*
**Kcnk5**	0.81	1.96	*0.41*	1.01	2.07	*0.49*	1.21	1.34	*0.90*
**Kif27**	1.06	0.78	*1.37*	1.55	1.00	*1.54*	1.01	0.87	*1.16*
**Klf10**	0.77	1.12	*0.68*	0.63	1.18	*0.53*	1.14	1.07	*1.06*
**Klf13**	0.79	1.48	*0.53*	0.66	1.25	*0.52*	1.03	1.10	*0.94*
**Klf6**	0.89	1.29	*0.69*	0.67	1.05	*0.64*	1.58	1.74	*0.91*
**Klf9**	0.91	1.42	*0.64*	0.79	1.52	*0.52*	0.89	1.06	*0.84*
**Klhl21**	0.94	1.17	*0.81*	0.90	1.32	*0.68*	1.09	1.14	*0.96*
**Lama1**	1.05	0.83	*1.26*	1.06	0.75	*1.41*	1.07	0.99	*1.08*
**Ldoc1l**	1.39	0.61	*2.26*	1.60	0.53	*3.00*	1.14	0.81	*1.39*
**Leo1**	1.24	0.66	*1.88*	1.34	0.76	*1.75*	1.14	1.00	*1.14*
**Lonrf1**	1.01	1.42	*0.71*	0.93	1.52	*0.61*	0.89	1.06	*0.84*
**Lpin1**	0.77	2.21	*0.35*	0.85	2.43	*0.35*	0.91	0.89	*1.02*
**Lrrc40**	0.89	1.11	*0.80*	0.89	1.16	*0.77*	0.88	0.92	*0.96*
**Lyrm1**	0.86	1.06	*0.81*	1.00	1.37	*0.73*	0.86	0.86	*1.00*

**Map3k6**	0.67	1.88	*0.35*	0.71	1.85	*0.38*	1.20	1.40	*0.86*
**Mbd1**	0.91	1.40	*0.65*	0.88	1.26	*0.70*	0.98	1.04	*0.94*
**Mknk2**	0.92	1.35	*0.68*	0.82	1.32	*0.62*	1.12	1.13	*0.99*
**Mpp4**	0.59	1.27	*0.47*	0.92	1.62	*0.57*	0.78	0.94	*0.83*
**Mreg**	0.75	1.52	*0.49*	0.97	1.36	*0.71*	0.87	0.97	*0.90*
**Myo9a**	1.01	0.79	*1.28*	1.13	0.82	*1.38*	1.11	1.18	*0.94*
**Nampt**	0.88	1.31	*0.67*	0.81	1.54	*0.53*	0.83	0.94	*0.88*
**Nat2**	0.79	1.68	*0.47*	0.94	1.83	*0.51*	0.91	0.93	*0.98*
**Ncald**	1.18	0.87	*1.35*	1.32	0.92	*1.43*	0.89	0.87	*1.03*
**Nfkbia**	0.85	1.62	*0.52*	0.71	1.38	*0.51*	1.27	1.34	*0.95*
**Nid2**	1.11	0.78	*1.42*	1.29	0.85	*1.51*	0.92	1.05	*0.87*
**Npas2**	1.02	0.10	*10.11*	2.92	0.37	*7.92*	1.35	0.57	*2.37*
**Nr1d1**	0.90	1.15	*0.79*	0.64	1.03	*0.62*	0.82	0.74	*1.12*
**Nr1d2**	0.73	1.88	*0.39*	0.61	2.34	*0.26*	0.77	1.16	*0.67*
**Nr1h4**	0.86	1.22	*0.70*	0.89	1.46	*0.61*	0.81	0.84	*0.96*
**Nrg4**	0.67	1.39	*0.48*	0.87	1.56	*0.56*	0.83	0.91	*0.92*
**Oplah**	0.94	1.35	*0.70*	0.72	1.37	*0.53*	1.00	1.12	*0.89*
**Osbpl9**	0.88	1.55	*0.56*	0.89	1.69	*0.53*	0.91	1.19	*0.77*
**Pank1**	1.12	1.50	*0.75*	1.01	1.48	*0.68*	0.80	0.78	*1.02*
**Paox**	0.89	1.39	*0.64*	0.92	1.28	*0.72*	1.02	0.91	*1.13*
**Paqr7**	1.01	1.25	*0.81*	0.85	1.22	*0.70*	0.83	0.82	*1.01*
**Pck1**	0.79	1.63	*0.49*	1.15	2.19	*0.53*	0.72	0.76	*0.95*
**Pdk1**	0.96	1.19	*0.81*	0.85	1.18	*0.72*	0.84	0.91	*0.92*
**Per1**	0.45	1.94	*0.23*	0.59	3.11	*0.19*	1.09	1.35	*0.81*
**Per2**	0.43	2.46	*0.17*	0.36	3.30	*0.11*	0.79	1.52	*0.52*
**Per3**	0.53	1.87	*0.28*	0.49	2.85	*0.17*	0.69	1.24	*0.55*
**Pex11a**	0.98	1.39	*0.71*	0.82	1.25	*0.66*	1.02	1.11	*0.92*
**Pik3ip1**	0.77	1.25	*0.62*	0.82	1.25	*0.65*	0.98	1.04	*0.94*
**Pim3**	0.77	1.78	*0.43*	1.11	2.40	*0.46*	0.89	1.03	*0.86*
**Plek2**	0.91	1.25	*0.73*	1.07	1.63	*0.65*	0.89	0.87	*1.03*
**Plk3**	0.51	2.54	*0.20*	0.93	3.90	*0.24*	1.10	1.75	*0.63*
**Pnpla2**	0.79	1.27	*0.62*	0.85	1.37	*0.62*	1.02	0.97	*1.05*
**Pnpla7**	0.85	1.31	*0.65*	0.87	1.31	*0.66*	0.96	0.86	*1.12*
**Podn**	0.79	1.21	*0.65*	0.89	1.35	*0.66*	0.97	0.92	*1.05*
**Ppm1h**	1.11	0.69	*1.61*	1.20	0.74	*1.62*	1.14	0.95	*1.20*
**Prkag3**	0.56	1.80	*0.31*	0.66	1.45	*0.46*	1.05	1.27	*0.83*
**Ptgis**	0.78	1.54	*0.51*	0.75	1.53	*0.49*	0.97	1.09	*0.89*
**Ptprd**	1.01	1.08	*0.93*	0.98	1.35	*0.73*	0.87	0.75	*1.15*
**Ptprk**	1.10	0.82	*1.35*	1.32	0.95	*1.39*	0.97	0.96	*1.01*
**Pxmp2**	0.80	1.26	*0.63*	1.02	1.48	*0.69*	0.70	0.64	*1.09*
**Rab43**	0.90	1.28	*0.71*	0.80	1.14	*0.70*	0.98	1.02	*0.97*

**Rab6b**	0.90	1.28	*0.71*	0.59	0.98	*0.60*	1.08	1.24	*0.87*
**Rabep2**	0.78	0.98	*0.79*	0.87	1.16	*0.75*	1.06	1.02	*1.04*
**Rasl11a**	1.06	0.70	*1.52*	1.03	0.56	*1.86*	1.22	1.17	*1.04*
**Rgs2**	0.68	1.39	*0.49*	0.80	1.25	*0.64*	1.03	1.03	*1.00*
**Ripk4**	0.89	1.28	*0.70*	0.99	1.62	*0.61*	0.98	1.01	*0.97*
**Rorc**	0.77	1.81	*0.43*	0.93	1.71	*0.54*	0.90	1.18	*0.76*
**Rtkn**	0.83	1.24	*0.67*	0.86	1.38	*0.62*	0.99	0.98	*1.02*
**Sema7a**	0.74	1.30	*0.56*	0.70	1.22	*0.58*	1.05	1.41	*0.74*
**Serpina7**	1.27	0.42	*3.01*	1.77	0.78	*2.26*	1.72	1.05	*1.64*
**Sgk1**	0.84	2.59	*0.32*	1.06	3.23	*0.33*	1.30	1.60	*0.81*
**Slc13a2**	0.81	1.38	*0.59*	0.95	1.68	*0.56*	0.76	0.69	*1.10*
**Slc22a12**	1.54	0.85	*1.80*	1.55	0.69	*2.24*	0.89	0.65	*1.37*
**Slc25a20**	0.83	1.42	*0.58*	0.87	1.52	*0.57*	0.86	1.02	*0.84*
**Slc25a33**	0.84	1.67	*0.50*	0.95	1.75	*0.54*	0.94	0.96	*0.99*
**Slc25a34**	0.92	1.22	*0.75*	0.85	1.41	*0.60*	0.87	0.92	*0.94*
**Slc46a3**	0.86	1.56	*0.55*	0.85	1.86	*0.45*	0.72	0.91	*0.79*
**Smarca2**	0.92	1.22	*0.76*	0.91	1.22	*0.75*	0.86	0.90	*0.95*
**Smpdl3b**	0.91	1.75	*0.52*	0.59	0.98	*0.60*	2.36	2.48	*0.95*
**Snrk**	0.97	1.43	*0.68*	0.96	1.39	*0.69*	0.98	1.01	*0.97*
**Spsb3**	1.11	0.80	*1.38*	1.12	0.77	*1.45*	1.07	0.98	*1.09*
**Srebf1**	1.19	0.82	*1.45*	1.43	0.90	*1.59*	0.96	0.86	*1.12*
**St5**	1.03	0.78	*1.32*	1.10	0.72	*1.53*	1.09	0.98	*1.11*
**St6galnac2**	0.76	1.16	*0.66*	0.86	1.10	*0.78*	1.17	1.17	*1.00*
**Stc2**	1.22	0.80	*1.54*	0.93	0.60	*1.54*	1.60	1.32	*1.21*
**Stk35**	0.87	1.14	*0.77*	0.94	1.30	*0.73*	1.00	1.08	*0.92*
**Stx18**	1.29	0.83	*1.56*	1.53	0.89	*1.72*	1.19	0.92	*1.29*
**Sult1a1**	0.71	1.36	*0.52*	1.03	1.86	*0.55*	1.03	0.88	*1.17*
**Sult1d1**	1.01	1.17	*0.87*	0.94	1.24	*0.76*	0.99	1.00	*0.98*
**Susd3**	0.77	2.09	*0.37*	0.84	2.41	*0.35*	0.83	1.03	*0.80*
**Syt12**	0.65	1.20	*0.54*	0.83	1.05	*0.79*	1.12	1.14	*0.98*
**Taf1d**	0.82	0.98	*0.83*	0.91	1.18	*0.77*	1.14	1.00	*1.14*
**Tchhl1**	1.20	0.66	*1.83*	1.38	0.91	*1.51*	1.06	1.04	*1.01*
**Tef**	0.69	1.62	*0.43*	0.63	2.00	*0.32*	0.69	1.16	*0.59*
**Tmem108**	1.20	0.74	*1.63*	1.48	0.81	*1.82*	0.98	0.93	*1.05*
**Tmem218**	1.17	0.89	*1.31*	1.40	0.99	*1.42*	0.96	0.84	*1.13*
**Tmem57**	0.90	1.27	*0.71*	0.79	1.19	*0.67*	0.89	1.00	*0.88*
**Tnfrsf21**	0.87	1.70	*0.51*	0.77	1.96	*0.40*	0.95	1.20	*0.80*
**Tnnc1**	0.76	1.62	*0.47*	0.68	1.98	*0.34*	0.93	1.26	*0.74*
**Tnxb**	0.86	1.29	*0.67*	0.71	1.28	*0.56*	0.83	0.95	*0.87*
**Tob2**	0.86	2.19	*0.39*	0.67	1.66	*0.40*	1.40	1.31	*1.07*
**Trib1**	0.86	1.33	*0.65*	0.75	1.21	*0.62*	1.15	1.02	*1.12*

**Tsc22d3**	0.74	2.85	*0.26*	0.85	2.81	*0.30*	1.12	1.47	*0.76*
**Tsku**	0.71	1.98	*0.36*	0.67	1.35	*0.49*	0.96	1.11	*0.86*
**Tspan14**	1.16	0.92	*1.26*	1.25	0.93	*1.35*	1.01	0.98	*1.03*
**Tspan4**	0.70	1.54	*0.45*	0.76	1.33	*0.57*	0.93	1.11	*0.84*
**Ttn**	1.34	0.72	*1.85*	1.38	0.98	*1.42*	0.99	1.11	*0.90*
**Ttpa**	0.91	1.60	*0.57*	0.85	1.22	*0.70*	1.00	1.05	*0.95*
**Tuba4a**	1.21	0.84	*1.44*	1.24	0.92	*1.35*	1.03	0.98	*1.05*
**Tut1**	0.85	1.19	*0.71*	0.93	1.24	*0.75*	1.02	0.99	*1.03*
**Unc5c**	1.37	0.75	*1.84*	1.37	0.84	*1.63*	1.03	0.87	*1.18*
**Upp2**	0.83	1.99	*0.42*	0.87	2.04	*0.42*	0.76	0.98	*0.78*
**Usp2**	0.73	2.30	*0.32*	0.72	2.33	*0.31*	0.98	1.07	*0.91*
**Vasn**	0.85	1.28	*0.66*	0.92	1.47	*0.63*	1.12	1.21	*0.92*
**Wnt5b**	0.83	1.61	*0.52*	0.94	2.01	*0.47*	0.79	0.88	*0.90*
**Xdh**	0.84	1.30	*0.65*	0.73	1.22	*0.60*	1.02	1.15	*0.89*
**Not corrected**									
**Symbol**									
**4930404N11Rik**	*0.70*	*1.08*	*0.65*	*1.24*	*0.88*	*1.41*	*0.99*	*0.97*	*1.02*
**Aacs**	*1.12*	*1.42*	*0.79*	*1.10*	*1.98*	*0.55*	*0.69*	*0.80*	*0.86*
**Abcd3**	*0.95*	*1.21*	*0.79*	*0.79*	*1.24*	*0.64*	*0.90*	*0.98*	*0.92*
**Acot11**	*1.04*	*1.05*	*0.99*	*0.92*	*1.21*	*0.76*	*0.90*	*0.87*	*1.02*
**Acot12**	*1.15*	*1.33*	*0.86*	*0.88*	*1.48*	*0.60*	*0.81*	*0.84*	*0.96*
**Acsm5**	*0.98*	*1.02*	*0.96*	*1.05*	*1.35*	*0.78*	*0.79*	*0.74*	*1.07*
**Acvr2b**	*0.87*	*1.03*	*0.85*	*0.61*	*1.77*	*0.34*	*0.93*	*1.09*	*0.85*
**Alox12**	*1.00*	*1.02*	*0.98*	*0.95*	*1.41*	*0.67*	*0.95*	*1.03*	*0.92*
**Asah2**	*1.21*	*1.01*	*1.20*	*0.80*	*1.11*	*0.72*	*0.96*	*0.91*	*1.05*
**Atp1b1**	*1.11*	*1.00*	*1.11*	*0.81*	*1.11*	*0.73*	*1.01*	*0.98*	*1.04*
**Avpr1a**	*1.29*	*0.69*	*1.85*	*1.81*	*0.56*	*3.22*	*1.10*	*0.85*	*1.30*
**BC022687**	*1.10*	*0.81*	*1.35*	*1.60*	*0.68*	*2.36*	*0.98*	*0.79*	*1.23*
**Calr**	*1.13*	*0.80*	*1.41*	*1.52*	*0.82*	*1.87*	*1.23*	*1.05*	*1.17*
**Cd300lg**	*1.03*	*1.27*	*0.81*	*0.81*	*1.46*	*0.55*	*0.66*	*0.65*	*1.01*
**Cdkn1a**	*0.86*	*1.52*	*0.56*	*1.05*	*0.56*	*1.88*	*1.88*	*1.68*	*1.12*
**Cdr2**	*1.13*	*1.38*	*0.82*	*1.01*	*1.42*	*0.71*	*1.05*	*1.14*	*0.92*
**Chrna4**	*0.96*	*0.75*	*1.27*	*1.87*	*0.77*	*2.44*	*0.83*	*0.78*	*1.06*
**Corin**	*1.19*	*0.41*	*2.89*	*0.85*	*1.48*	*0.57*	*0.65*	*0.81*	*0.80*
**Csdc2**	*1.06*	*0.83*	*1.28*	*1.33*	*0.89*	*1.49*	*1.15*	*0.97*	*1.18*
**Ctps**	*0.97*	*1.12*	*0.87*	*0.76*	*1.07*	*0.71*	*1.20*	*1.23*	*0.98*
**D630023F18Rik**	*1.15*	*0.87*	*1.33*	*1.84*	*0.69*	*2.69*	*1.03*	*0.77*	*1.33*
**Ddc**	*1.81*	*0.75*	*2.42*	*3.04*	*0.69*	*4.39*	*1.06*	*0.69*	*1.53*
**Defb1**	*1.17*	*0.93*	*1.26*	*1.60*	*0.95*	*1.69*	*0.90*	*0.76*	*1.18*
**Dlat**	*1.16*	*1.06*	*1.09*	*0.85*	*1.16*	*0.73*	*0.93*	*0.89*	*1.05*

**Dnajc6**	*1.30*	*0.99*	*1.31*	*0.86*	*1.72*	*0.50*	*0.68*	*0.72*	*0.94*
**Dtx4**	*0.92*	*0.67*	*1.37*	*1.29*	*0.43*	*3.02*	*1.44*	*1.11*	*1.29*
**Elfn1**	*1.83*	*0.97*	*1.89*	*0.78*	*1.31*	*0.59*	*1.05*	*0.76*	*1.38*
**Enox1**	*1.17*	*1.12*	*1.04*	*1.01*	*1.56*	*0.65*	*1.06*	*1.27*	*0.84*
**Entpd5**	*1.08*	*1.05*	*1.03*	*0.81*	*1.23*	*0.65*	*0.80*	*0.93*	*0.86*
**Esrrb**	*1.31*	*1.13*	*1.16*	*0.89*	*1.42*	*0.63*	*0.91*	*0.89*	*1.02*
**Ethe1**	*1.21*	*0.70*	*1.72*	*2.23*	*0.41*	*5.49*	*1.25*	*0.83*	*1.51*
**G0s2**	*0.91*	*1.30*	*0.70*	*1.52*	*1.05*	*1.45*	*0.85*	*0.77*	*1.11*
**Gdpd2**	*0.60*	*1.01*	*0.60*	*1.25*	*0.78*	*1.61*	*1.23*	*1.01*	*1.22*
**Gfpt2**	*1.18*	*0.87*	*1.36*	*1.30*	*0.85*	*1.52*	*1.04*	*0.87*	*1.20*
**Il15**	*0.91*	*1.00*	*0.91*	*0.91*	*1.49*	*0.61*	*0.74*	*0.91*	*0.81*
**Kcnab2**	*1.00*	*1.25*	*0.80*	*1.14*	*0.77*	*1.47*	*1.19*	*1.09*	*1.09*
**Kif13a**	*1.17*	*1.08*	*1.09*	*0.88*	*1.22*	*0.72*	*1.00*	*1.01*	*0.99*
**Klf1**	*0.93*	*0.96*	*0.97*	*1.03*	*1.61*	*0.64*	*0.81*	*0.80*	*1.01*
**Lgmn**	*0.87*	*1.12*	*0.77*	*1.20*	*0.85*	*1.41*	*1.06*	*1.11*	*0.95*
**Lhfpl2**	*1.03*	*0.75*	*1.37*	*1.18*	*0.66*	*1.78*	*1.29*	*1.19*	*1.08*
**Lpin2**	*1.27*	*0.92*	*1.37*	*0.76*	*1.02*	*0.75*	*1.12*	*1.12*	*1.00*
**Lrig3**	*1.06*	*1.00*	*1.06*	*0.84*	*1.18*	*0.71*	*0.98*	*1.04*	*0.94*
**Masp2**	*1.24*	*0.78*	*1.59*	*0.91*	*1.44*	*0.64*	*1.03*	*0.85*	*1.21*
**Mif4gd**	*1.11*	*0.84*	*1.32*	*1.33*	*0.84*	*1.58*	*1.08*	*0.96*	*1.13*
**Mmp15**	*1.09*	*0.76*	*1.43*	*1.12*	*0.60*	*1.85*	*1.22*	*1.05*	*1.16*
**Mpp5**	*1.08*	*1.14*	*0.95*	*0.85*	*1.13*	*0.75*	*0.96*	*0.99*	*0.97*
**Mpv17l**	*1.32*	*1.04*	*1.26*	*0.94*	*1.67*	*0.56*	*0.77*	*0.90*	*0.86*
**Mtss1**	*0.94*	*1.10*	*0.85*	*0.91*	*1.35*	*0.67*	*0.95*	*1.03*	*0.92*
**Narf**	*0.94*	*1.11*	*0.85*	*0.76*	*1.15*	*0.66*	*0.90*	*0.96*	*0.94*
**Neu2**	*1.05*	*1.19*	*0.89*	*0.75*	*1.27*	*0.59*	*0.79*	*0.88*	*0.90*
**Nfib**	*0.91*	*1.06*	*0.85*	*0.91*	*1.27*	*0.72*	*0.85*	*0.94*	*0.91*
**Ngef**	*0.94*	*1.17*	*0.80*	*0.88*	*1.42*	*0.62*	*0.82*	*0.84*	*0.98*
**Notch4**	*0.95*	*0.99*	*0.96*	*0.85*	*1.13*	*0.76*	*0.98*	*1.02*	*0.96*
**Nphs2**	*1.27*	*0.88*	*1.44*	*1.63*	*0.77*	*2.12*	*1.12*	*1.14*	*0.99*
**Nudt19**	*1.40*	*1.93*	*0.73*	*0.98*	*2.24*	*0.44*	*0.49*	*0.67*	*0.74*
**Osr2**	*1.05*	*0.78*	*1.35*	*1.67*	*1.05*	*1.59*	*0.99*	*0.80*	*1.23*
**P2ry14**	*0.88*	*1.00*	*0.88*	*0.69*	*1.21*	*0.57*	*0.97*	*1.15*	*0.84*
**Pcdh17**	*1.02*	*0.94*	*1.09*	*0.73*	*1.32*	*0.55*	*0.72*	*0.80*	*0.90*
**Pcgf5**	*1.11*	*1.21*	*0.92*	*0.87*	*1.29*	*0.67*	*0.97*	*1.05*	*0.93*
**Pcsk9**	*1.18*	*0.86*	*1.37*	*2.93*	*0.90*	*3.25*	*1.03*	*0.73*	*1.42*
**Polg2**	*1.01*	*1.07*	*0.94*	*0.96*	*1.35*	*0.71*	*0.79*	*0.79*	*1.01*
**Por**	*1.07*	*1.21*	*0.88*	*0.88*	*1.69*	*0.52*	*0.78*	*0.90*	*0.87*
**Ppargc1a**	*1.10*	*0.93*	*1.18*	*0.74*	*1.09*	*0.68*	*0.97*	*1.00*	*0.98*
**Ppp1r9a**	*1.00*	*0.78*	*1.28*	*0.90*	*1.15*	*0.78*	*0.93*	*0.93*	*0.99*
**Ppp2r5e**	*0.99*	*1.15*	*0.86*	*0.87*	*1.17*	*0.74*	*0.99*	*1.03*	*0.96*

**Ppp3cc**	*1.03*	*1.19*	*0.86*	*0.89*	*1.30*	*0.68*	*0.91*	*0.93*	*0.97*
**Prmt2**	*1.16*	*0.90*	*1.29*	*1.13*	*0.71*	*1.61*	*1.15*	*1.09*	*1.06*
**Prnp**	*0.93*	*1.38*	*0.67*	*0.77*	*1.55*	*0.49*	*0.93*	*1.19*	*0.77*
**Ptpn22**	*0.68*	*1.31*	*0.51*	*0.99*	*0.61*	*1.60*	*1.21*	*1.42*	*0.85*
**Rsad1**	*0.98*	*1.07*	*0.92*	*0.84*	*1.17*	*0.72*	*0.97*	*0.91*	*1.06*
**Rxrg**	*1.42*	*0.97*	*1.45*	*1.75*	*0.83*	*2.11*	*1.00*	*0.64*	*1.57*
**Sall2**	*1.04*	*0.79*	*1.31*	*1.62*	*0.81*	*1.99*	*1.03*	*0.82*	*1.26*
**Sdc3**	*0.89*	*1.07*	*0.84*	*0.62*	*1.05*	*0.59*	*1.02*	*1.28*	*0.80*
**Sema3f**	*1.00*	*1.09*	*0.92*	*0.74*	*0.95*	*0.78*	*1.17*	*1.19*	*0.98*
**Slc12a6**	*0.85*	*1.15*	*0.74*	*0.78*	*1.73*	*0.45*	*0.85*	*1.05*	*0.81*
**Slc16a1**	*1.23*	*0.85*	*1.44*	*1.74*	*0.86*	*2.03*	*1.14*	*0.99*	*1.15*
**Slc16a9**	*0.87*	*1.41*	*0.61*	*0.67*	*1.94*	*0.34*	*0.66*	*0.94*	*0.70*
**Slc17a2**	*1.17*	*0.81*	*1.45*	*1.10*	*2.24*	*0.49*	*0.62*	*0.48*	*1.30*
**Slc1a3**	*1.10*	*1.16*	*0.95*	*0.83*	*1.40*	*0.59*	*0.89*	*1.05*	*0.85*
**Slc22a4**	*0.96*	*1.19*	*0.81*	*0.89*	*1.50*	*0.59*	*0.71*	*0.83*	*0.85*
**Slc22a5**	*0.98*	*1.25*	*0.78*	*0.83*	*1.41*	*0.59*	*0.84*	*0.95*	*0.88*
**Slc25a35**	*1.04*	*0.98*	*1.06*	*0.97*	*1.24*	*0.78*	*0.88*	*0.86*	*1.02*
**Slc25a42**	*0.89*	*1.43*	*0.62*	*0.76*	*1.70*	*0.45*	*0.84*	*1.09*	*0.77*
**Slc27a2**	*1.19*	*1.05*	*1.14*	*0.88*	*1.26*	*0.70*	*0.83*	*0.91*	*0.92*
**Slc2a13**	*1.74*	*0.99*	*1.75*	*0.91*	*1.83*	*0.50*	*0.79*	*0.87*	*0.90*
**Slc5a2**	*0.78*	*1.18*	*0.66*	*1.96*	*1.08*	*1.82*	*0.95*	*0.84*	*1.13*
**Slc5a3**	*1.69*	*1.06*	*1.60*	*0.80*	*1.14*	*0.70*	*1.26*	*1.09*	*1.16*
**Slc5a6**	*1.16*	*1.22*	*0.95*	*0.91*	*2.02*	*0.45*	*0.66*	*0.62*	*1.06*
**Slc5a8**	*1.29*	*1.66*	*0.77*	*0.85*	*2.08*	*0.41*	*0.79*	*0.85*	*0.93*
**Slc7a8**	*1.23*	*0.86*	*1.43*	*2.24*	*0.73*	*3.05*	*1.14*	*0.79*	*1.45*
**Slco2a1**	*1.03*	*1.09*	*0.95*	*0.91*	*1.26*	*0.72*	*0.97*	*0.98*	*0.99*
**Slit2**	*0.98*	*1.34*	*0.73*	*0.90*	*1.60*	*0.56*	*0.83*	*0.93*	*0.90*
**Spsb4**	*1.25*	*0.87*	*1.43*	*1.50*	*0.78*	*1.92*	*1.10*	*0.93*	*1.18*
**Srd5a1**	*0.95*	*1.07*	*0.89*	*0.77*	*1.04*	*0.73*	*1.10*	*1.19*	*0.92*
**Stim1**	*1.06*	*1.21*	*0.87*	*0.81*	*1.28*	*0.64*	*1.00*	*1.04*	*0.97*
**Stk32b**	*1.07*	*1.26*	*0.85*	*0.95*	*1.53*	*0.62*	*0.92*	*0.99*	*0.93*
**Stxbp4**	*1.09*	*0.90*	*1.21*	*0.84*	*1.46*	*0.57*	*0.94*	*1.05*	*0.90*
**Sult1c2**	*1.18*	*0.90*	*1.31*	*1.40*	*0.91*	*1.54*	*0.97*	*0.81*	*1.20*
**Sumo3**	*1.15*	*0.86*	*1.34*	*1.36*	*0.81*	*1.68*	*1.08*	*0.94*	*1.14*
**Sypl2**	*0.91*	*1.26*	*0.72*	*0.93*	*1.67*	*0.55*	*0.75*	*0.97*	*0.77*
**Tbxas1**	*1.07*	*0.70*	*1.52*	*2.28*	*0.94*	*2.44*	*0.90*	*0.68*	*1.33*
**Timm8a1**	*0.98*	*1.08*	*0.91*	*0.90*	*1.26*	*0.71*	*0.84*	*0.91*	*0.92*
**Tmem45b**	*0.88*	*1.06*	*0.83*	*0.88*	*1.24*	*0.72*	*0.89*	*0.97*	*0.93*
**Tmem53**	*1.07*	*0.84*	*1.27*	*1.50*	*0.95*	*1.57*	*0.95*	*0.80*	*1.19*
**Tppp**	*1.03*	*1.17*	*0.88*	*1.06*	*1.52*	*0.70*	*0.79*	*0.84*	*0.93*
**Trak2**	*1.18*	*1.02*	*1.16*	*0.88*	*1.20*	*0.74*	*0.92*	*0.97*	*0.94*

**Trp53bp2**	*1.07*	*1.13*	*0.95*	*0.88*	*1.30*	*0.67*	*0.99*	*0.94*	*1.05*
**Ttc22**	*1.06*	*0.66*	*1.61*	*1.36*	*0.70*	*1.96*	*1.11*	*0.86*	*1.29*
**Vps37d**	*0.98*	*0.70*	*1.39*	*1.22*	*0.63*	*1.94*	*1.10*	*0.72*	*1.52*
**Wsb1**	*0.95*	*0.60*	*1.58*	*1.71*	*0.64*	*2.65*	*1.17*	*1.11*	*1.06*
**Yaf2**	*1.17*	*1.12*	*1.05*	*0.88*	*1.15*	*0.76*	*0.91*	*0.95*	*0.97*
**Zbtb7a**	*1.12*	*1.03*	*1.09*	*0.83*	*1.16*	*0.71*	*1.16*	*1.22*	*0.95*
**Zc3h6**	*0.98*	*0.97*	*1.01*	*0.84*	*1.08*	*0.78*	*0.83*	*0.83*	*1.00*
**Zfp612**	*0.86*	*0.88*	*0.98*	*0.85*	*1.14*	*0.74*	*1.00*	*1.07*	*0.93*
**Zhx3**	*1.31*	*1.22*	*1.08*	*0.89*	*1.52*	*0.59*	*1.11*	*1.26*	*0.88*

Supplementary Table 5: Pathway analysis of 320 diurnally regulated genes corrected and not corrected by remission induction				
**Corrected by remission induction**				
**Categories**	**Diseases or Functions Annotation**	**Molecules**	**p-value**	**# Molecules**
Behavior,Nervous System Development and Function	Circadian rhythm	ARNTL,CLOCK,CRY2,DBP,KLF10,NAMPT,NPAS2,NR1D1,NR1D2,PER1,PER2,PER3,SREBF1	6.52E-11	13
Cancer,Organismal Injury and Abnormalities	Cancer,Organismal Injury and Abnormalities	ACAT2,ACOT1,ACOX3,AIFM2,AK5,ALAS1,AMT,ANGPTL4,ANKRD12,AQP8,ARHGEF3,ARNTL,AZIN1,BLVRB,C11orf65,CA5B,CDC42EP4,CDKL1,CDO1,CHST1,CHST8,CITED4,CKB,CLCN2,CLDN10,CLOCK,CLPX,CLSTN3,COL27A1,COL4A4,COQ10B,CORO2A,CPEB3,CPT2,CREB3L1,CRY2,CSNK1G3,CTGF,CYGB,CYP27A1,CYP2A6 (includes others), P2E1,DBP,DCUN1D3,DGAT2,DHRS7,DLEU7,DNASE1,DUSP1,DUSP7,EEF2K,EFHD1,EIF4EBP3,EPHX1,ERRFI1,FABP3,FAM102A,FAM163A,FERMT1,FFAR3,FKBP5,FOXO1,GJA1,GLDC,GLUL,GNPDA1,GRASP,GSTT2/GSTT2B,GYPC,HERPUD1,HSPB6,IDH1,IER3,IFRD2,IGFBP4,IGFBP5,IL17F,IL17RB,IMMP2L,INSC,ITGA6,ITGB6,ITPRIP,KCNJ15,KCNK5,KIF27,KLF10,KLF13,KLF6,KLF9,KLHL21,LAMA1,LEO1,LONRF1,LPIN1,LRRC40,LYRM1,MAP3K6,MBD1,MKNK2,MPP4,MREG,MYO9A,NAMPT,NAT1,NCALD,NFKBIA,NID2,NPAS2,NR1D1,NR1D2,NR1H4,OSBPL9,PANK1,PAOX,PAQR7,PCK1,PDK1,PER1,PER2,PER3,PEX11A,PIK3IP1,PIM3,PLEK2,PLK3,PNPLA2,PNPLA7,PODN,PPM1H,PRKAG3,PTGIS,PTPRK,PXMP2,RAB43,RAB6B,RABEP2,RASL11A,RGS2,RIPK4,RORC,RTKN,SEMA7A,SERPINA7,SGK1,SLC13A2,SLC22A12,SLC25A20,SLC25A33,SLC25A34,SLC46A3,SMARCA2,SMPDL3B,SNRK,SPSB3,SREBF1,ST5,ST6GALNAC2,STC2,STK35,STX18,SUS	6.62E-08	185

Cancer,Organismal Injury and Abnormalities	Cancer,Organismal Injury and Abnormalities	ACAT2,ACOT1,ACOX3,AIFM2,AK5,ALAS1,AMT,ANGPTL4,ANKRD12,AQP8,ARHGEF3,ARNTL,AZIN1,BLVRB,C11orf65,CA5B,CDC42EP4,CDKL1,CDO1,CHST1,CHST8,CITED4,CKB,CLCN2,CLDN10,CLOCK,CLPX,CLSTN3,COL27A1,COL4A4,COQ10B,CORO2A,CPEB3,CPT2,CREB3L1,CRY2,CSNK1G3,CTGF,CYGB,CYP27A1,CYP2A6 (includes others), P2E1,DBP,DCUN1D3,DGAT2,DHRS7,DLEU7,DNASE1,DUSP1,DUSP7,EEF2K,EFHD1,EIF4EBP3,EPHX1,ERRFI1,FABP3,FAM102A,FAM163A,FERMT1,FFAR3,FKBP5,FOXO1,GJA1,GLDC,GLUL,GNPDA1,GRASP,GSTT2/GSTT2B,GYPC,HERPUD1,HSPB6,IDH1,IER3,IFRD2,IGFBP4,IGFBP5,IL17F,IL17RB,IMMP2L,INSC,ITGA6,ITGB6,ITPRIP,KCNJ15,KCNK5,KIF27,KLF10,KLF13,KLF6,KLF9,KLHL21,LAMA1,LEO1,LONRF1,LPIN1,LRRC40,LYRM1,MAP3K6,MBD1,MKNK2,MPP4,MREG,MYO9A,NAMPT,NAT1,NCALD,NFKBIA,NID2,NPAS2,NR1D1,NR1D2,NR1H4,OSBPL9,PANK1,PAOX,PAQR7,PCK1,PDK1,PER1,PER2,PER3,PEX11A,PIK3IP1,PIM3,PLEK2,PLK3,PNPLA2,PNPLA7,PODN,PPM1H,PRKAG3,PTGIS,PTPRK,PXMP2,RAB43,RAB6B,RABEP2,RASL11A,RGS2,RIPK4,RORC,RTKN,SEMA7A,SERPINA7,SGK1,SLC13A2,SLC22A12,SLC25A20,SLC25A33,SLC25A34,SLC46A3,SMARCA2,SMPDL3B,SNRK,SPSB3,SREBF1,ST5,ST6GALNAC2,STC2,STK35,STX18,SUSD3,SYT12,TAF1D,TCHHL1,TEF,TMEM108,TNFRSF21,TNXB,TOB2,TRIB1,TSC22D3,TSKU,TSPAN14,TSPAN4,TTN,TTPA,TUBA4A,TUT1,UNC5C,UPP2,USP2,VASN,WNT5B,XDH	6.62E-08	185
Carbohydrate Metabolism,Small Molecule Biochemistry	Synthesis of D-hexose	FOXO1,GNPDA1,IDH1,NR1H4,PCK1,RORC,SREBF1,USP2	4.97E-06	8

Carbohydrate Metabolism,Molecular Transport	Quantity of glycogen	DGAT2,DUSP1,FABP3,FOXO1,NR1H4,PCK1,PNPLA2,PRKAG3	9.15E-06	8
Carbohydrate Metabolism,Molecular Transport,Small Molecule Biochemistry	Concentration of D-glucose	ARNTL,CA5B,CDO1,CRY2,DGAT2,FABP3,FOXO1,IGFBP4,IGFBP5,LPIN1,NR1H4,PANK1,PCK1,PNPLA2,SREBF1	1.64E-05	15
Carbohydrate Metabolism,Cellular Function and Maintenance,Small Molecule Biochemistry	Homeostasis of D-glucose	ANGPTL4,ARNTL,CRY2,FFAR3,FOXO1,IGFBP4,IGFBP5,NR1H4,PCK1,PRKAG3,TSC22D3	4.82E-05	11
Carbohydrate Metabolism	Quantity of carbohydrate	ARNTL,CA5B,CDO1,CHST1,CRY2,CTGF,DGAT2,DUSP1,FABP3,FOXO1,IGFBP4,IGFBP5,LPIN1,NR1H4,PANK1,PCK1,PNPLA2,PRKAG3,SREBF1	6.66E-05	19
Cell Death and Survival	Apoptosis	AIFM2,ANGPTL4,AQP11,ARHGEF3,CLCN2,COL4A4,CTGF,CYGB,CYP2E1,DCUN1D3,DLEU7,DNASE1,DUSP1,EEF2K,EPHX1,ERRFI1,FFAR3,FKBP5,FOXO1,GJA1,HERPUD1,HSPB6,IER3,IGFBP4,IGFBP5,IL17RB,ITGA6,KLF10,KLF13,KLF6,KLF9,LAMA1,NAMPT,NFKBIA,NR1D1,NR1H4,NRG4,PDK1,PER1,PIK3IP1,PIM3,PLK3,PNPLA2,PTGIS,RORC,RTKN,SEMA7A,SGK1,SMARCA2,SNRK,SREBF1,STK35,TNFRSF21,TRIB1,TSC22D3,TTN,TTPA,UNC5C,USP2,VASN,XDH	2.86E-05	61
Cell Death and Survival,Organismal Injury and Abnormalities	Necrosis of epithelial tissue	CYGB,CYP2E1,EEF2K,FOXO1,IER3,IGFBP5,ITGA6,KLF10,NAMPT,NFKBIA,NR1H4,NRG4,PLK3,PTGIS,SEMA7A,SGK1,STK35,TNFRSF21,USP2,VASN,XDH	3.41E-05	21

Cell Death and Survival	Necrosis	AQP11,ARHGEF3,AVPI1,Clec2d (includes others),CLOCK,CREB3L1,CTGF,CYGB,CYP2E1,DLEU7,DNASE1,DUSP1,EEF2K,EPHX1,FABP3,FKBP5,FOXO1,GJA1,HERPUD1,HSPB6,IER3,IGFBP4,IGFBP5,IL17RB,ITGA6,ITPRIP,KLF10,KLF13,KLF6,KLF9,MBD1,NAMPT,NFKBIA,NR1D1,NR1H4,NRG4,PDK1,PER1,PEX11A,PIK3IP1,PIM3,PLK3,PNPLA2,PTGIS,RORC,RTKN,SEMA7A,SGK1,SNRK,SREBF1,STK35,TNFRSF21,TRIB1,TSC22D3,TTN,TTPA,UNC5C,USP2,VASN,XDH	7.05E-05	60
Lipid Metabolism,Molecular Transport,Small Molecule Biochemistry	Concentration of triacylglycerol	ACAT2,ANGPTL4,AQP8,ARNTL,CYP27A1,DGAT2,DUSP1,FFAR3,FOXO1,IDH1,LPIN1,NAMPT,NR1H4,PANK1,PCK1,PEX11A,PNPLA2,SNRK,SREBF1,TRIB1,XDH	6.42E-10	21
Lipid Metabolism,Molecular Transport,Small Molecule Biochemistry	Concentration of fatty acid	ANGPTL4,ARNTL,CYP27A1,CYP2E1,DGAT2,DUSP1,FABP3,FOXO1,IDH1,LPIN1,NR1H4,PANK1,PCK1,PEX11A,PNPLA2,PTGIS,SNRK,SREBF1,XDH	2.37E-09	19
Metabolic Disease,Organismal Injury and Abnormalities	Abnormal metabolism	ACAT2,ANGPTL4,ARNTL,CPT2,CTGF,FOXO1,IDH1,IGFBP4,IGFBP5,ITGB6,LPIN1,NR1H4,PANK1,PCK1,PNPLA2,SLC25A20,SREBF1,TNFRSF21,TRIB1	2.11E-07	19
Lipid Metabolism,Molecular Transport,Small Molecule Biochemistry	Concentration of lipid	ACAT2,ANGPTL4,AQP8,ARNTL,CHST8,CLOCK,CRY2,CYP27A1,CYP2E1,DGAT2,DUSP1,FABP3,FFAR3,FOXO1,IDH1,LPIN1,NAMPT,NR1H4,PANK1,PCK1,PEX11A,PNPLA2,PTGIS,SGK1,SNRK,SREBF1,TRIB1,TSC22D3,TTPA,XDH	4.85E-07	30
Lipid Metabolism,Small Molecule Biochemistry	Homeostasis of lipid	ANGPTL4,ARNTL,CYP27A1,DGAT2,FABP3,NR1D1,NR1D2,NR1H4,PNPLA2,SREBF1,TTPA,XDH	3.53E-06	12
Lipid Metabolism,Small Molecule Biochemistry	Metabolism of acylglycerol	CYP2E1,DGAT2,EPHX1,FABP3,FOXO1,LPIN1,PNPLA2,RGS2,SREBF1,TNXB	3.98E-06	10
Lipid Metabolism,Molecular Transport,Small Molecule	Accumulation of triacylglycerol	ACAT2,FOXO1,LPIN1,NR1H4,PNPLA2,PRKAG3,SREBF1	9.29E-05	7

Biochemistry				
Organ Morphology,Organismal Development	Mass of genitourinary system	AQP8,ARNTL,CHST8,CYP27A1,DUSP1,ERRFI1,FFAR3,IGFBP4,NR1H4,PANK1,PCK1,PNPLA2,TRIB1,TSC22D3	2.57E-05	14
Organismal Development	Development of genitourinary system	AQP11,AQP8,ARNTL,CDO1,CHST8,CLCN2,CLOCK,COL4A4,CTGF,CYP27A1,DUSP1,EEF2K,ERRFI1,FFAR3,GJA1,IGFBP4,IGFBP5,IMMP2L,ITGA6,KLF6,NR1H4,PANK1,PCK1,PNPLA2,SREBF1,TOB2,TRIB1,TSC22D3,TTPA,XDH	2.01E-05	30
Cardiovascular System Development and Function,Hematological System Development and Function	Blood pressure	ARNTL,DBP,DUSP1,FFAR3,IER3,KCNK5,NFKBIA,PTGIS,RGS2,SGK1,TEF,TTN	0.00029	12
Cardiovascular Disease,Organismal Injury and Abnormalities	Atherosclerosis	ACAT2,ANGPTL4,CA5B,CORO2A,CPEB3,CYP27A1,FOXO1,GJA1,KLF6,NFKBIA,NR1H4,SREBF1,TTPA,TUBA4A,XDH	0.0013	15
Cardiovascular Disease,Organismal Injury and Abnormalities	Occlusion of artery	ACAT2,ANGPTL4,CA5B,CORO2A,CPEB3,CYP27A1,FOXO1,GJA1,KLF6,NFKBIA,NR1H4,SREBF1,TTN,TTPA,TUBA4A,XDH	0.0017	16
**Not corrected by remission induction**				
**Categories**	**Diseases or Functions Annotation**	**Molecules**	**p-value**	**# Molecules**

Cancer,Organismal Injury and Abnormalities	Cancer,Organismal Injury and Abnormalities	AACS,ABCD3,ACOT11,ACOT12,ACSM5,ACVR2B,ALOX12,ASAH2,ATP1B1,AVPR1A,C19orf71,CALR,CD300LG,CDKN1A,CDR2,CHRNA4,CLBA1,CORIN,CSDC2,CTPS1,DDC,DLAT,DNAJC6,DTX4,ELFN1,ENOX1,ENTPD5,ESRRB,ETHE1,G0S2,GDPD2,GFPT2,IL15,KCNAB2,KIF13A,KLF1,LGMN,LHFPL2,LPIN2,LRIG3,MASP2,MIF4GD,MMP15,MPP5,MPV17L,MTSS1,NARF,NEU2,NFIB,NGEF,NOTCH4,NPHS2,NUDT19,OSR2,P2RY14,PCDH17,PCGF5,PCSK9,POLG2,POR,PPARGC1A,PPP1R9A,PPP2R5E,PPP3CC,PRMT2,PRNP,PTPN22,RSAD1,RXRG,SALL2,SDC3,SEMA3F,SLC12A6,SLC16A1,SLC16A9,SLC17A2,SLC1A3,SLC22A4,SLC22A5,SLC25A35,SLC25A42,SLC27A2,SLC2A13,SLC5A2,SLC5A3,SLC5A6,SLC5A8,SLC7A8,SLCO2A1,SLIT2,SPSB4,SRD5A1,STIM1,STK32B,STXBP4,SULT1C2,SUMO3,SYPL2,TBXAS1,TIMM8A,TMEM45B,TMEM53,TP53BP2,TPPP,TRAK2,TTC22,VPS37D,WSB1,ZBTB7A,ZC3H6,ZHX3,ZNF23	9.69E-11	112
Lipid Metabolism,Small Molecule Biochemistry	Fatty acid metabolism	AACS,ABCD3,ACOT11,ACOT12,ALOX12,ASAH2,AVPR1A,DLAT,IL15,NUDT19,POR,PPARGC1A,PRNP,SLC16A1,SLC1A3,SLC22A5,SLC27A2,SLCO2A1,STIM1,TBXAS1	7.77E-09	20
Energy Production,Lipid Metabolism,Small Molecule Biochemistry	Oxidation of lipid	ABCD3,ACOT11,ALOX12,G0S2,IL15,POR,PPARGC1A,SLC22A5,SLC27A2,SLCO2A1	1.47E-06	10
Lipid Metabolism,Molecular Transport,Small Molecule Biochemistry	Concentration of lipid	ACOT11,ALOX12,ASAH2,AVPR1A,CDKN1A,ENTPD5,G0S2,NEU2,PCSK9,POR,PPARGC1A,PRNP,RXRG,SLC5A3,SLCO2A1,SRD5A1	0.000451	16
Lipid Metabolism,Small Molecule Biochemistry	Synthesis of lipid	ABCD3,ALOX12,ASAH2,AVPR1A,DLAT,IL15,LPIN2,POR,PPARGC1A,PRNP,SLC1A3,SLC27A2,SRD5A1,STIM1,TBXAS1	0.00107	15
Molecular Transport	Transport of carboxylic acid	ABCD3,PRNP,SLC16A1,SLC1A3,SLC27A2,SLC5A6,SLC5A8,SLCO2A1	3.13E-07	8

Molecular Transport	Transport of molecule	ABCD3,ATP1B1,AVPR1A,CALR,CHRNA4,DDC,IL15,KIF13A,PPARGC1A,PRNP,PTPN22,SLC12A6,SLC16A1,SLC17A2,SLC1A3,SLC22A4,SLC22A5,SLC25A42,SLC27A2,SLC2A13,SLC5A2,SLC5A3,SLC5A6,SLC5A8,SLC7A8,SLCO2A1,STIM1,TRAK2	3.21E-05	28
Organismal Survival	Organismal death	ACVR2B,ALOX12,ATP1B1,CALR,CDKN1A,CHRNA4,Defb1,DNAJC6,ESRRB,ETHE1,IL15,KCNAB2,KLF1,MIF4GD,MMP15,MTSS1,NFIB,NOTCH4,NPHS2,POLG2,POR,PPARGC1A,PPP1R9A,PRNP,SALL2,SLC1A3,SLC22A4,SLC22A5,SLC5A3,SLCO2A1,SLIT2,STIM1,SUMO3,TP53BP2,ZBTB7A	0.000197	35
Cell Death and Survival	Apoptosis of tumor cell lines	ACSM5,ACVR2B,ALOX12,CALR,CDKN1A,ENTPD5,ETHE1,G0S2,IL15,NEU2,NFIB,NOTCH4,PPARGC1A,PRNP,PTPN22,SALL2,SEMA3F,SLC5A8,STIM1,SUMO3,TP53BP2,WSB1	0.000274	22
